# *De novo* Sequencing and Transcriptome Analysis Reveal Key Genes Regulating Steroid Metabolism in Leaves, Roots, Adventitious Roots and Calli of *Periploca sepium* Bunge

**DOI:** 10.3389/fpls.2017.00594

**Published:** 2017-04-21

**Authors:** Jian Zhang, Xinglin Li, Fuping Lu, Shanying Wang, Yunhe An, Xiaoxing Su, Xiankuan Li, Lin Ma, Guangjian Han

**Affiliations:** ^1^Key Lab of Industrial Fermentation Microbiology, Tianjin University of Science and Technology, Ministry of EducationTianjin, China; ^2^School of Traditional Chinese Materia Medica, Tianjin University of Traditional Chinese MedicineTianjin, China; ^3^College of Bioengineering, Tianjin University of Science and TechnologyTianjin, China; ^4^Beijing Center for Physical and Chemical AnalysisBeijing, China; ^5^Shachuan BiotechnologyTianjin, China

**Keywords:** bioactive steroid biosynthesis, Illumina sequencing, *Periploca sepium* Bunge, secondary metabolism, transcriptome comparison

## Abstract

*Periploca sepium* Bunge is a traditional medicinal plant, whose root bark is important for Chinese herbal medicine. Its major bioactive compounds are C21 steroids and periplocin, a kind of cardiac glycoside, which are derived from the steroid synthesis pathway. However, research on *P. sepium* genome or transcriptomes and their related genes has been lacking for a long time. In this study we estimated this species nuclear genome size at 170 Mb (using flow cytometry). Then, RNA sequencing of four different tissue samples of *P. sepium* (leaves, roots, adventitious roots, and calli) was done using the sequencing platform Illumina/Solexa Hiseq 2,500. After *de novo* assembly and quantitative assessment, 90,375 all-transcripts and 71,629 all-unigenes were finally generated. Annotation efforts that used a number of public databases resulted in detailed annotation information for the transcripts. In addition, differentially expressed genes (DEGs) were identified by using digital gene profiling based on the reads per kilobase of transcript per million reads mapped (RPKM) values. Compared with the leaf samples (L), up-regulated genes and down-regulated genes were eventually obtained. To deepen our understanding of these DEGs, we performed two enrichment analyses: gene ontology (GO) and Kyoto Encyclopedia of Genes and Genomes (KEGG). Here, the analysis focused upon the expression characteristics of those genes involved in the terpene metabolic pathway and the steroid biosynthesis pathway, to better elucidate the molecular mechanism of bioactive steroid synthesis in *P. sepium*. The bioinformatics analysis enabled us to find many genes that are involved in bioactive steroid biosynthesis. These genes encoded acetyl-CoA acetyltransferase (ACAT), HMG-CoA synthase (HMGS), HMG-CoA reductase (HMGR), mevalonate kinase (MK), phosphomevalonate kinase (PMK), mevalonate diphosphate decarboxylase (MDD), isopentenylpyrophosphate isomerase (IPPI), farnesyl pyrophosphate synthase (FPS), squalene synthase (SS), squalene epoxidase (SE), cycloartenol synthase (CAS), sterol C-24 methyltransferase (SMT1), sterol-4alpha-methyl oxidase 1 (SMO1), sterol 14alpha-demethylase (CYP51/14-SDM), delta(14)-sterol reductase (FK/14SR), C-8,7 sterol isomerase (HYD1), sterol-4alpha-methyl oxidase 2 (SMO2), delta(7)-sterol-C5(6)-desaturase (STE1/SC5DL), 7-dehydrocholesterol reductase (DWF5/DHCR7), delta (24)-sterol reductase (DWF1/DHCR24), sterol 22-desaturase (CYP710A), progesterone 5beta-reductase (5β-POR), 3-beta-hydroxysteroid dehydrogenase (3β-HSD). This research will be helpful to further understand the mechanism of bioactive steroid biosynthesis in *P. sepium*, namely C21 steroid and periplocin biosynthesis.

## Introduction

*Periploca sepium* Bunge is a traditional medicinal plant of the Asclepiadaceae family that is widely distributed in the northern temperate regions of China. The dried root bark of *P. sepium*, known as “Cortex Periplocae,” is used as a Chinese medicinal herb for the treatment of rheumatism, cancer, inflammation, and cardiac failure (Xu et al., 1990; Umehara et al., 1995; Yin et al., 2009; Ding et al., 2014). Both C21steroids and periplocin, a kind of cardiac glycoside, are the major bioactive constituents occurring in the root bark of *P. sepium*. To date, more than 30 bioactive compounds of C21 steroids and periplocin have been isolated from *P. sepium* (Yin et al., 2009; Ding et al., 2014), all of which belong to the class of steroid derivatives.

The main biosynthesis pathway of steroids has been researched extensively (Darnet and Rahier, 2004; Gunaherath and Gunatilaka, 2014). The upstream pathway of steroid biosynthesis has been confirmed primarily through the mevalonic acid (MVA) pathway, followed by the cycloartenol or lanosterol pathways, which can catalyze isoprene into various plant steroids (Benveniste, 2004; Li et al., 2013). However, the biosynthesis of steroid derivatives remains largely unknown, especially for secondary metabolites, such as C21 steroids and cardiac glycoside. The former are a class of steroid derivatives characterized by 21 carbon atoms. Presently, the known C21 steroids are based on the simple framework of a pregnane or its isomer. Further, research has showed that the pregnane derivatives are intermediates in cardiac glycoside biosynthesis for which cholesterol is a direct precursor (Sauer et al., 1969; Lindemann and Luckner, 1997). Presumably some genes are related to the biosynthesis of pregnane derivatives, such as the genes encoding cholesterol monooxygenase/side chain-cleaving enzyme (SCCE), 5βPOR, 3βHSD, delta 5-delta 4-ketosteroid isomerase (KSI), pregnane 14βhydroxylase (P14β), and others (Kreis and Müller-Ur, 2012; Zheng et al., 2014).

In the present study, we cultured *P. sepium* sterile seedlings and induced adventitious roots and calli in the root explants. First, we determined the genome size of *P. sepium* by using flow cytometry. Next, we extracted the RNA from the roots, leaves, adventitious roots, and calli to construct their complementary DNA (cDNA) libraries for sequencing their transcriptomes. Our objectives were to explore the transcriptome profiling of the non-medal plant, and to then study the biosynthesis of the bioactive steroids, namely periplocin or C21 steroids. Through a bioinformatics analysis, we were able to investigate the putative biosynthetic pathway of these bioactive steroids in *P. sepium*.

## Materials and methods

### Plant materials

The collected seeds of *P. sepium* were first washed under running tap water. Then, their surface was sterilized with 75% ethanol for 30 s followed by soaking in 5% NaOCl solution for 30 min, after which the seeds were rinsed with sterile water. These sterilized seeds were wiped up and transferred into a tissue culture vessel containing 50 ml of Murashige and Skoog (MS) basal medium supplemented with 0.7% (*w/v*) agar. These seeds were cultured at 25 ± 2°C under a 16-/8-h (day/night) photoperiod for 1 month so they could develop into seedlings.

**Figure 1:**
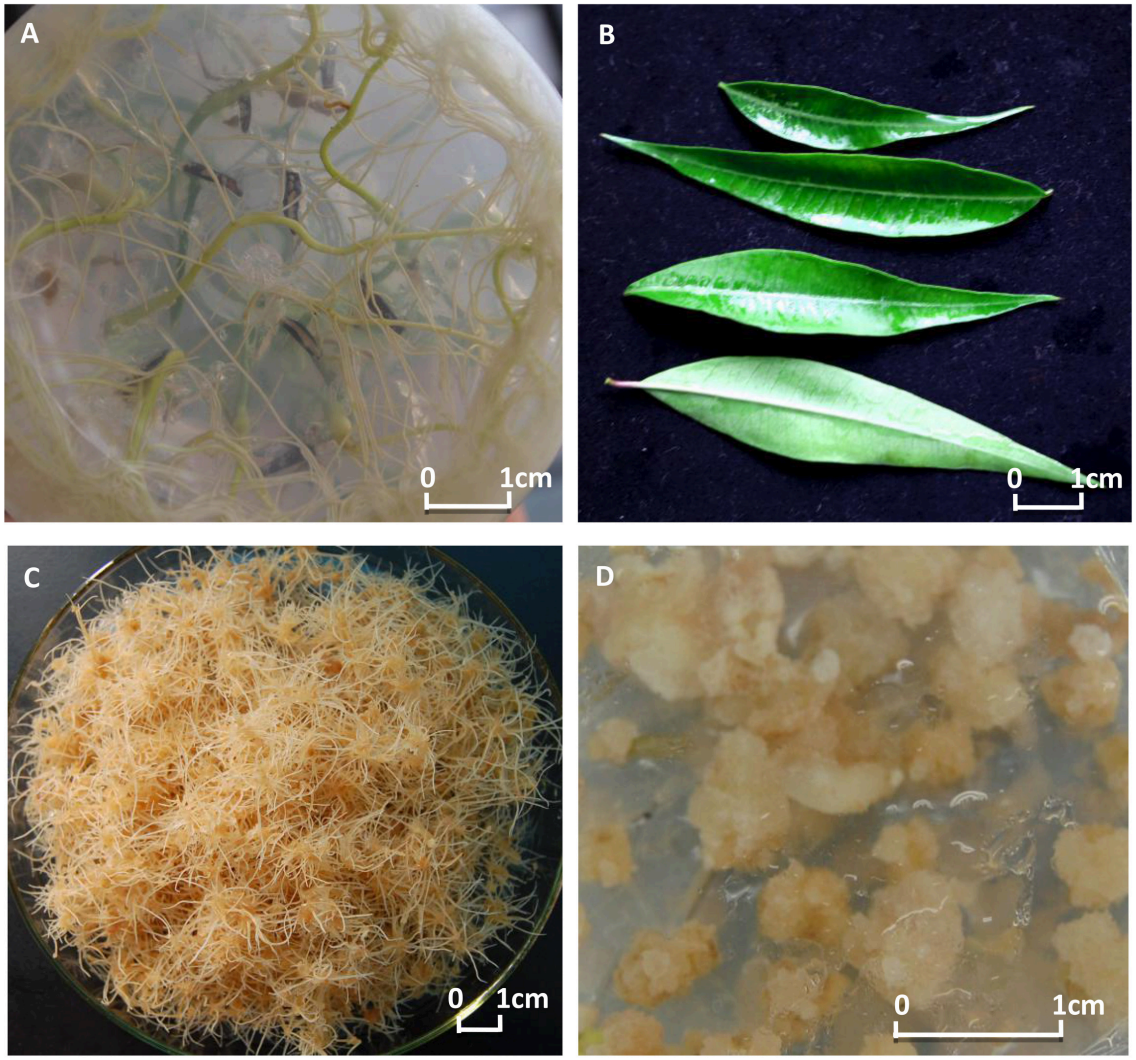
**The explants and the induced tissue cultures of *Periploca sepium***. Shown are the explants of roots **(A)** and leaves **(B)**, and the induced tissue cultures of adventitious roots **(C)** and calli **(D)**.

The roots and leaves were then obtained from the 1-month old seedlings. The selected roots were placed on two types of solid MS medium to induce adventitious roots and calli, respectively. For adventitious root induction, we used a 1/2 MS medium supplemented with 1 mg/l indole butyric acid (IBA), 3% (*w/v*) sucrose, and 0.7% (*w/v*) agar in Petri dishes (Zhang et al., 2011a, 2012)—for callus induction, we used an MS medium supplemented with 2 mg/l IBA, 1 mg/l 6-Benzylaminopurine (6-BA), 3% (*w/v*) sucrose, and 0.7% (*w/v*) agar in Petri dishes (Zhang et al., 2010b). Cultures were kept under darkness at 23 ± 2°C. Four weeks later, the induced adventitious roots and calli were obtained from the root explants (Figure 1). From these, actively growing samples were selected for extracting total RNA from roots for sequencing. Experiments for extracting RNA were conducted in triplicate, and the total mixed RNA was used for sequencing. The same method was followed to extract total RNA from leaves, adventitious roots, and calli.

### Nuclear DNA content determination

The young leaves from the seedlings of *Arabidopsis* (CK) and *P. sepium* were collected and homogenized with a sharp blade in a 2-ml homogenization buffer (45 mM MgCl_2_; 30 mM sodium citrate; 20 mM 3-morpholinopropanesulfonic acid (MOPS); 0.1% (*w/v*) TritonX-100; pH 7.0) and filtered through a 200-mesh nylon netting (Galbraith et al., 1983). Then, the nuclei portion was obtained by centrifuging the suspension at 1,500 rpm for 5 min. Next, the nuclei suspension was stained with PI (50 μg/ml), incubated at 4°C for 20 min, and then filtered once more through a 500-mesh nylon netting. The resulting nuclei suspensions were examined by flow cytometry. From the same tissue sample three sets of measurements were obtained.

### Total RNA extraction and cDNA library construction

The mRNA extraction, cDNA library construction and sequencing were performed by the Beijing Center for Physical and Chemical Analysis (BCPCA) (Beijing, China). Total RNA was extracted from each sample using the TRlzol reagent and digested with DNase I. Oligo (dT) magnetic beads were used to enrich mRNA from the total RNA and then broken into short fragments by a fragmentation buffer. To build the cDNA libraries based on the four samples (Solexa/Illumina 2,500 platform: 101 bp short insert-paired end), we followed the methods of Neeraja et al. (2012) and Tang et al. (2011).

### Sequencing, *de novo* assembly and sequence annotation

The libraries of the four samples were each sequenced by an Illumina Hiseq 2,500 (Illumina, CA, USA). The total sequence nucleotides of each sample exceeded or approached 1 Gb, a depth equivalent to 5 times (5-fold coverage) the size of the *P. sepium* genome size (Chow et al., 2014). Raw reads were processed to delete those reads that had adaptors or that included more than five unknown nucleotides (“N”). Then, the low quality reads containing >20% of bases with a quality score ≤10 were removed to leave only high-quality reads for subsequent analysis. We used the Trinity software (Grabherr et al., 2011) for the *de novo* assembly of clean reads and the cd-hit (Li and Godzik, 2006), a fast program for clustering and comparing large sets of protein or nucleotide sequences, to remove the redundant Trinity-generated contigs. The resulting transcripts underwent annotation through Basic Local Alignment Search Tool (BLAST) searches (Altschul et al., 1990) against the Kyoto Encyclopedia of Genes and Genomes (KEGG) (Kanehisa and Goto, 2000) and National Centre for Biotechnology Information (NCBI) databases of non-redundant protein database (Nr) and non-redundant nucleotide database (Nt)—a cut-off *E*-value of 10^−5^ was in this annotation.

### Differential gene analysis and function enrichment

Clean reads were aligned to unigenes using the bowtie2 program. The reads per kilobase of exon per million mapped reads (RPKM) values (Mortazavi et al., 2008) were calculated for the unigenes from the leaves (L), roots (R), adventitious roots (AR), and calli (C). The differential expression of the unigenes was calculated by using the DEGseq software package (Wang et al., 2009) in which a MA-plot-based method coupled to a Random Sampling model (MARS) method was mainly used. This approach was supplemented by other robust methods—the Likelihood Ratio Test (LRT) (Marioni et al., 2008), the Fisher's Exact Test (FET) (Grans and Shuster, 2008) and the Fold-Change threshold on MA-plot (FC) method—for which significance was set a priori to a *p* ≤ 0.05 and |log2 (fold change)| > 1.

Next, we used both GO and KEGG (Kanehisa et al., 2012) enrichments to analyze the differentially expressed genes (DEGs). The GO enrichment used the Blast2GO suite in procedure that had three main steps: (1) blast to find the homologous sequences; (2) mapping to collect the GO terms associated with the blast hits; and (3) annotation to assign trustworthy information to the query sequences (Conesa and Götz, 2008). The KEGG enrichment was performed by the KEGG Automatic Annotation Server (KAAS) to define the KEGG orthologs (KOs) (Moriya et al., 2007), after which the KOs were mapped to their related KEGG pathways.

### Real-time PCR analysis for validation of gene expression

To verify the reliability of the results from the transcriptome-wide expression analysis, we selected nine genes all identified as DEGs in the adventitious roots, as compared with leaves involved in the known isopentenyl diphosphate (IPP) biosynthesis pathways. RNA from the adventitious roots and leaves was isolated and reverse-transcribed to single-strand cDNA. Quantitative reactions were performed on a Real-Time PCR Detection System (Bio-rad IQ5) using the SYBR Premix Ex Taq™ II (Shachuan Biotechnology, China). The reaction system (20 μl) contained 10 μl 2 × NI-RealMasterMix, 10 μM each of the forward and reverse primers, and 2 μl of cDNA template. The reaction was performed under the following conditions: 95°C for 3 min, followed by 40 cycles of 95°C for 15 s, 60°C for 1 min, and 72°C for 2 min. Expression levels of the target genes were normalized to that of 18S rRNA. Related gene expression levels were calculated using the 2^−ΔΔCt^ method (Livak and Schmittgen, 2001). All primers of the selected genes were listed in Table S1. These experiments were repeated thrice.

## Results

### Genome-size estimation

The genome size of *P. sepium* was determined by flow cytometry using the leaf nucleus, with *Arabidopsis* [≈125 Mb (Arabidopsis Genome Initiative 2000)] as an internal standard. The genome size of *P. sepium* [2*n* = 22 (Ma and Liu, 1988)] was estimated at 173.95 ± 2.20 Mb (Table 1 and Figure S1). This is the first report on genome size for *P. sepium*, a key result for determining the sequencing depth.

**Table 1:** **Flow cytometry determination of the nuclear genome size of *Periploca sepium***.

***P. sepium* peak**	**Reference peak^a^**	**Peak ratio (*P. sepium*/reference)**	***P. sepium* genome size (Mb mean ± SD)**
801	585	1.3692	171.1538
790	567	1.3932	174.1623
853	604	1.4122	176.5314
			173.95 ± 2.20

a *Nuclei from Arabidopsis young leaves served as a size standard; this species genome size is 125 Mb*.

### Illumina sequencing and reads assembly

After sequencing the four cDNA libraries, we obtained approximately 10 million 101-base pair (bp) paired-end raw reads for the leaves, 11 million for the roots, 18 million for the adventitious roots, and 9 million for the calli. After the adapter sequences and low quality reads were filtered out, we ultimately generated over 10, 11, 18, and 9 million clean reads for leaves, roots, adventitious roots and calli, respectively. Clean reads of each sample were assembled using Trinity software. Finally, 43,596 (L), 43,623 (R), 40,755 (AR), and 38,293 (C) transcripts were obtained. For each tissue sample, the average transcript size exceeded 800 bp, with an N50 of 1,400–1,600 bp. The transcripts of the four samples were then connected to unigenes by using Cd-hit program. The number of unigenes was 42,074 (L), 42,154 (R), 39,373 (AR), and 37,058 (C). After performing a long-sequence clustering of the four samples, 90,375 all-transcripts and 71,629 all-unigenes were finally generated; these had a mean length of 1,364.30 and 1,157.96 bp, with an N50 of 2,213 and 2,006 bp, respectively. The length distributions of the transcripts and unigenes are shown in Figure S2. A summary of the sequencing and assembly results is given in Table 2.

**Table 2:** **Summary of the Illumina sequencing and reads assembly for *Periploca sepium***.

	**Leaves (L)**	**Roots (R)**	**Adventitious roots (AR)**	**Calli (C)**	**Four tissues together**
No. of raw reads	10,421,244	11,618,452	18,049,608	9,256,330	49,345,634
Length of raw reads (bp)	1,052,545,644	1,173,463,652	1,823,010,408	934,889,330	4,983,909,034
No. of clean reads	9,113,194	10,447,392	10,955,178	8,115,614	38,631,378
Length of clean reads (bp)	909,071,085	1,055,831,908	1,089,663,942	812,300,948	3,866,867,883
No. of transcripts	43,596	43,623	40,755	38,293	90,375
Length of transcripts (bp)	38493,848	38,755,231	37,094,942	35,432,785	123,298,561
Average length of transcripts (bp)	883	888	910	925	1,364
N50 of transcripts (bp)	1,496	1,488	1,502	1,531	2,213
N90 of transcripts (bp)	342	348	363	368	648
No. of unigenes	42,074	42,154	39,373	37,058	71,629
Length of unigenes (bp)	35,963,540	36260,124	34,786,363	33,281,079	82,943,330
Average length of unigenes (bp)	855	860	883	898	1,158
N50 of unigenes (bp)	1,451	1,443	1,465	1,491	2,006
N90 of unigenes (bp)	331	338	352	357	460

### Functional annotation

**Figure 2:**
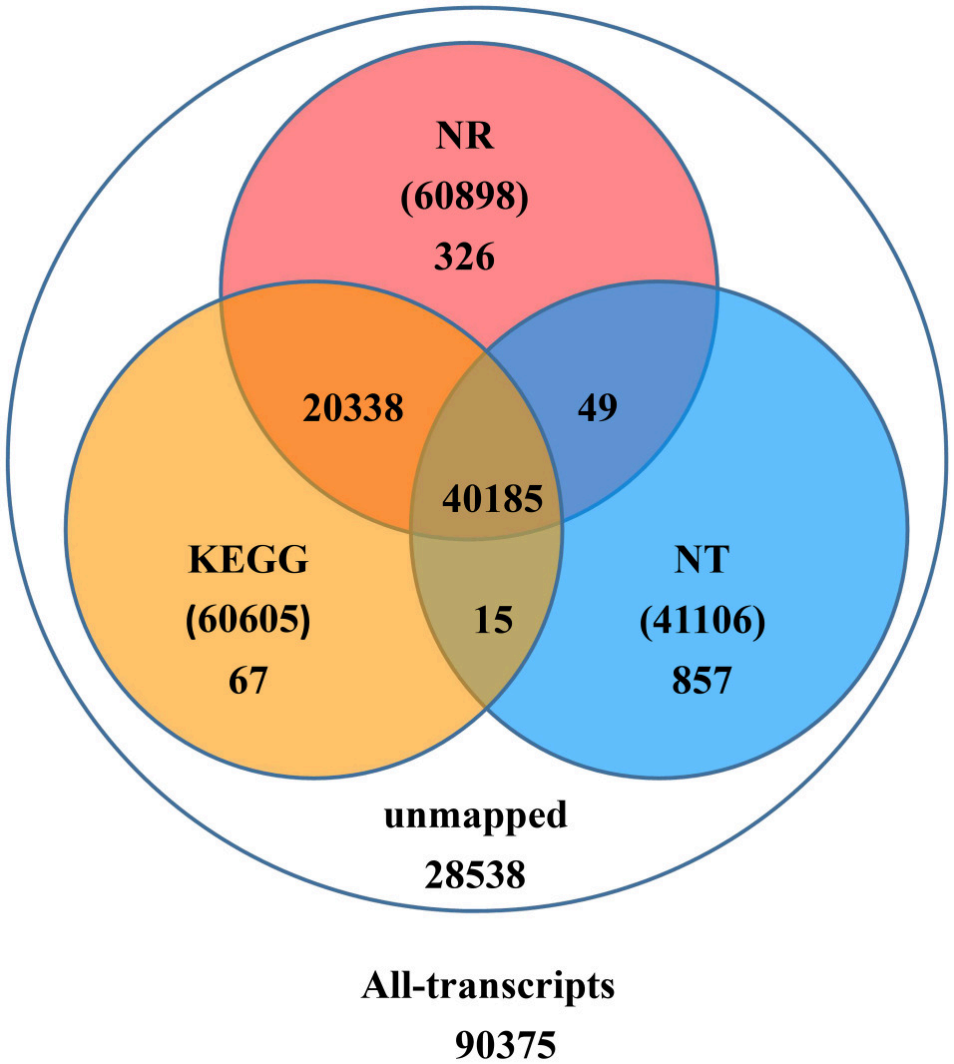
**Number of transcripts blasted to NR, NT, and KEGG (*E* < 0.00001)**.

Upon further investigation—performing the BLAST analysis of all-transcripts against KEGG and NCBI databases of Nr and Nt—there were 61,837 (68.4%) transcripts having homologous sequences in at least one of the above databases. Among these databases 41,106 (45.5%), 60,898 (67.4%), 60,605 (67.1%) transcripts were found in Nt, Nr, and KEGG, respectively. A total of 40,185 (44.5%) transcripts were found in all three databases, but 28,538 (31.6%) transcripts could not be identified (Figure 2).

**Figure 3:**
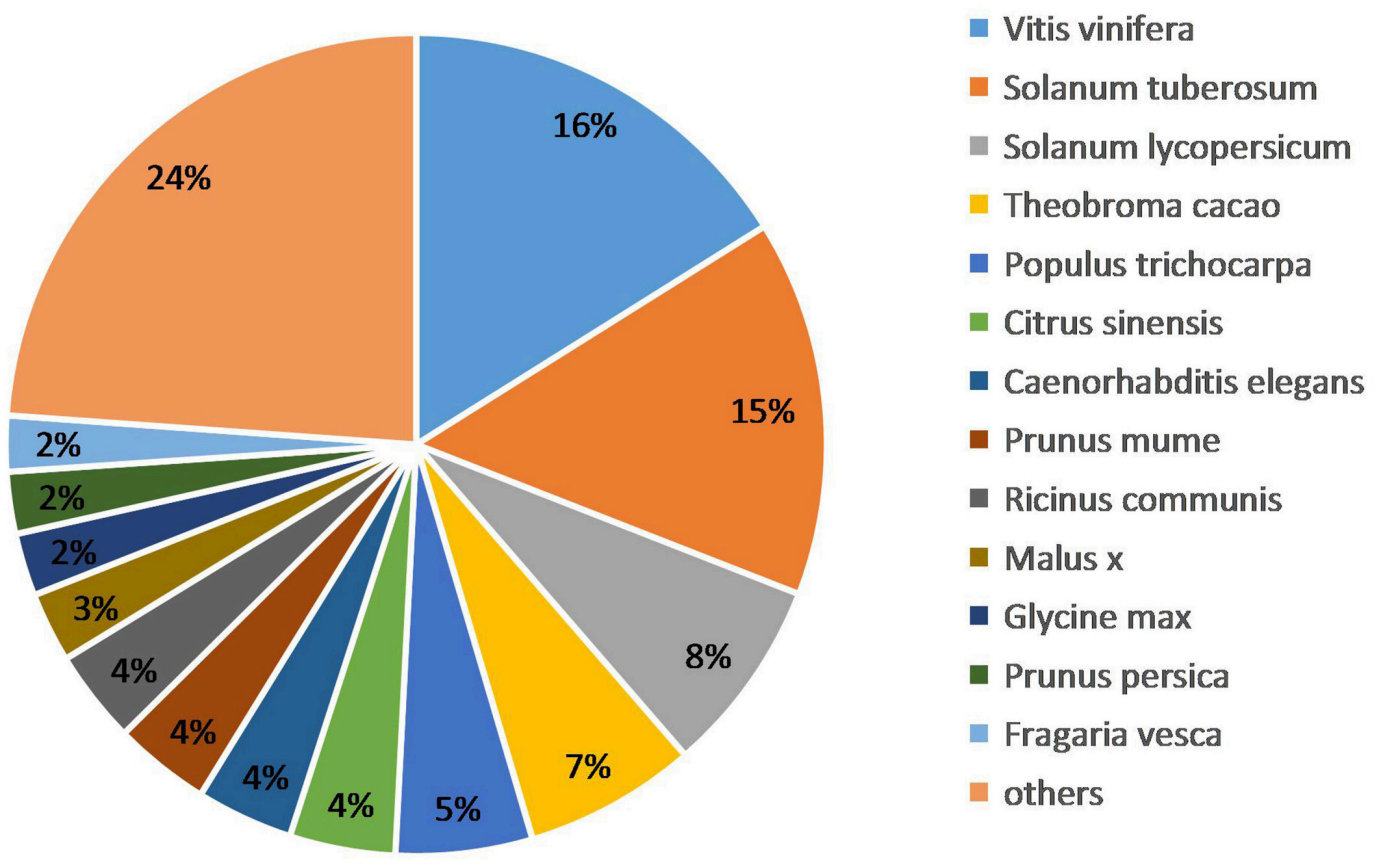
**Species homology analysis of the Nt annotation**.

We obtained the species homology distribution of *P. sepium* transcripts via the Nt annotation and consideration of 626 plant species that have homologous mRNA sequences as compared with *P. sepium*. The annotated transcripts yielded the best overlap with *Vitis vinifera* (16.11%), *Solanum tuberosum* (14.81%), and *Solanum lycopersicum* (7.71%) (Figure 3). The extent of overlap with different species approximately reflects the genetic relationships among them (Krishnan et al., 2011).

Moreover, we focused on those transcripts annotated to *Catharanthus roseus*, a plant closely related plant to *P. sepium*. Indeed, *C. roseus* belongs to Apocynaceae family, which has a closer genetic relationship with Asclepiadaceae family. The results showed that 366 transcripts were annotated to the Nt database that accounted for 0.89% of all transcripts. Furthermore, by blast in the latest Nt database, we got 1,369 genes of *C. roseus* registered in the Nt database. The results showed that the low proportion of *P. sepium* transcripts annotated to *C. roseus* reflected the too low number of *C. roseus* genes currently annotated to the Nt database. Hence, a mapping of the sequencing of the transcripts of *P. sepium* to the reference genome of *C. roseus* was performed (Kellner et al., 2015). Through blast analysis, about 43, 39, and 3% of the transcript sequences of *P. sepium* were found to have 60~80%, 80~95%, and 95~100% similarity with the genome of *C. roseus* (Data Sheet 1).

We then further studied these genes annotated to *C. roseus* in the Nt database. Interestingly, many annotated genes were related to the biosynthesis of terpene indole alkaloid, which occurs primarily in *C. roseus* (Verma et al., 2014; Zhu et al., 2014; Kellner et al., 2015). These genes included those located upstream that are involved in the terpene biosynthesis pathway, such as the genes encoding the enzymes of ACAT, HMGS, MK, PMK, MDD, FPS, DXP synthase (DXS), and geranyl diphosphate synthase (GPS), as well as downstream genes that are involved in trhe indole alkaloid biosynthesis pathway, such as the genes encoding the enzymes of 10-hydroxygeraniol oxidoreductase (10-HGO), desacetoxyvindoline 4-hydroxylase (D4H), and 16-hydroxytabersonine O-methyltransferase (16-OMT) (Table S2). Although an indole alkaloid has not yet been reported for *P. sepium*, the occurrence of these genes indicated that the terpene indole alkaloid biosynthesis pathway probably exists in *P. sepium*.

### Differential expression analysis

For transcript abundance, the expression levels of unigenes were calculated by RPKM. The result showed that, when compared with the leaves (L), a total of 2,132 (C), 1,723 (AR), and 1,508 (R) genes were up-regulated whereas 2,507 (C), 2,675 (AR), and 1,850 (R) genes were down-regulated (eventually) according to the MARS methodology, as supplemented by the methods of FET, LRT, and FC (Table S3). The distribution of gene changes is shown in Figure S4. Not surprisingly, we found that compared with leaves, the number of enriched DEGs in the tissue cultures (4,398 in adventitious roots and 4,639 in calli) were significantly higher than those in the roots (3,358). This phenomenon might be due to a difference in the culture environment. In this study, adventitious roots and calli were cultured under dark heterotrophic environment, and their growth and differentiation were induced by exogenous hormones, which were very different from the growth of the seedlings, resulting in related genes responding to stimulation of environmental changes. It needs further research.

Then we mapped the above DEGs to the GO database and calculated the gene numbers from each GO term. By using a hyper-geometric test, the significantly enriched GO terms in DEGs were identified as compared with the genomic background. The threshold for significance was *p* ≤ 0.05. The GO enrichment analysis results are shown in Tables S4–S6 and Figure S5. As shown in Figure S4, a total of 1,662 (AR vs. L), 2031 (C vs. L), and 1,127 (R vs. L) DEGs were successfully enriched and classified into three main categories: cellular component, molecular function, and biological process. These different unigenes were mainly focused on six GO terms consisting of cell part, cell, catalytic activity, binding, metabolic process, and cellular process, which together accounted for approximately 65–70% of the total differential unigenes.

By analyzing the top 20 terms of some important GO annotations (Tables S7–S9), we found that for all three samples compared with leaves, the terms from the biological process category enriched more DEGs compared with those from the cellular component and molecular function categories. Especially in calli compared with leaves, 11 of the top 12 terms were from the biological process category. Furthermore, over half of the terms of DEGs in calli compared with leaves were related to primary metabolism, thus matching the more vigorous growth of calli than other plant organs. This might represent the main difference between de-differentiated tissue cells and differentiated organ cells.

Based on the KEGG assignment, we followed up with a KEGG enrichment analysis for the sets of DEGs. The KEGG enrichment mapping assigned a total of 2,803 DEGs to 290 pathways (AR vs. L), 3,419 DEGs to 288 pathways (C vs. L), and 1,611 DEGs to 270 pathways (R vs. L) (Tables S10–S12). As shown in these tables, the terms for ribosome (ko03010), carbon metabolism (ko01200), biosynthesis of amino acids (ko01230), and plant hormone signal transduction (ko04075) occupied the top four terms of DEGs enrichment in adventitious roots or calli (as compared with leaves). Especially considering the ribosome and biosynthesis of amino acids, the number of these DEGs is much higher, at 3–5 times and more than 2 times, respectively, than that found in roots. Most of these DEGs expressed an up-regulated response (Figure S3). This indicated that the potential for cell protein synthesis under the tissue culture conditions was greatly enhanced to ensure the vigorous growth rate of the cultures. Additionally, several secondary metabolic pathways related to phenylpropanoid biosynthesis (ko00940), terpenoid backbone biosynthesis (ko00900), steroid biosynthesis (ko00100), and flavonoid biosynthesis (Ko00941) were also significantly enriched for DEGs (Table 3). The DEGs enriched in special secondary metabolic pathways are useful for analyzing several important secondary metabolites in *P. sepium*.

**Table 3:** **Secondary metabolic pathways and their related number of DEGs in the three samples as compared with leaves**.

**Secondary metabolic pathways**	**Pathway ID**	**Number of DEGs**		
		**R vs. L**	**AR vs. L**	**C vs. L**
Carotenoid biosynthesis	Ko00906	6	10	8
Cutin, suberine and wax biosynthesis	ko00073	4	2	0
Diterpenoid biosynthesis	ko00904	1	3	0
Flavonoid biosynthesis	ko00941	8	12	13
Isoquinoline alkaloid biosynthesis	ko00950	4	4	5
Limonene and pinene degradation	ko00903	3	5	5
Monoterpenoid biosynthesis	ko00902	0	2	0
Nicotinate and nicotinamide metabolism	ko00760	2	4	3
Sesquiterpenoid and triterpenoid biosynthesis	ko00909	5	8	10
Phenylpropanoid biosynthesis	ko00940	47	53	44
Steroid biosynthesis	ko00100	3	14	12
Stilbenoid, diarylheptanoid and gingerol biosynthesis	ko00945	8	9	11
Terpenoid backbone biosynthesis	ko00900	4	16	14
Tropane, piperidine and pyridine alkaloid biosynthesis	ko00960	6	5	7
Ubiquinone and other terpenoid-quinone biosynthesis	ko00130	8	10	8
Zeatin biosynthesis	ko00908	17	8	9
Degradation of aromatic compounds	ko01220	2	5	3
Brassinosteroid biosynthesis	ko00905	7	6	7
Chloroalkane and chloroalkene degradation	ko00625	3	7	5
Benzoate degradation	ko00362	0	3	4
Aminobenzoate degradation	ko00627	2	2	1

*AR, C, R, and L denote the adventitious roots, calli, roots, and leaves, respectively*.

### Expression characteristics of genes involved in the terpene metabolic pathway

**Figure 4:**
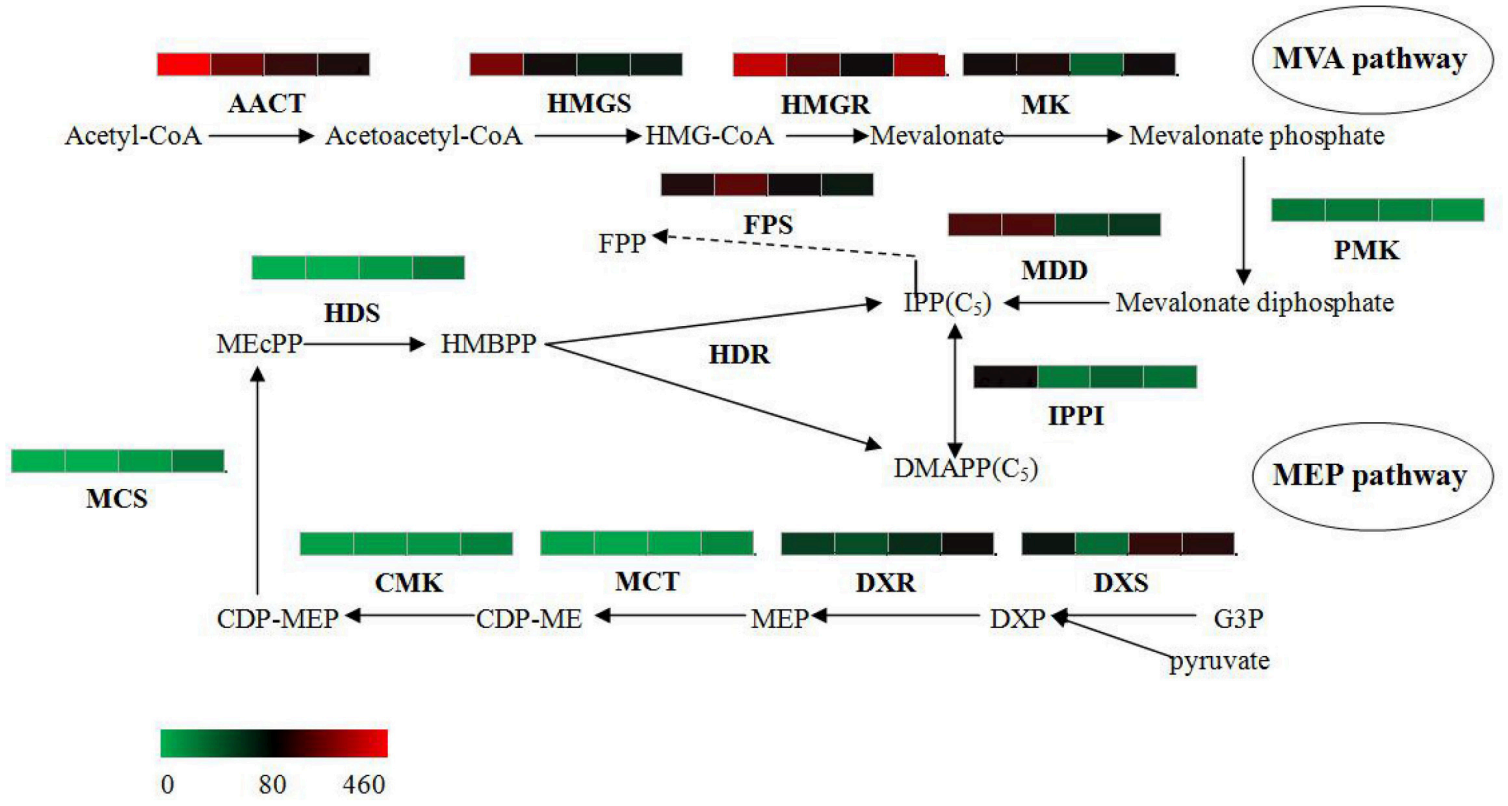
**Putative IPP and DMAPP biosynthesis in *Periploca sepium*.** Enzymes involved in IPP and DMAPP biosynthesis from the MVA and MEP pathways are shown. The expression of genes encoding these enzymes in AR, C, R, and L are shown by the heat-map. These genes were mapped using RPKM values, and they were color coded by increasing relative expression. IPP, isopentenyl diphosphate; DMAPP, dimethylallyl diphosphate; MVA, mevalonic acid; MEP, methylerythritol phosphate; ACAT, acetyl-CoA acetyltransferase; HMGS, HMG-CoA synthase; HMGR, HMG-CoA reductase; MK, mevalonate kinase; PMK, phosphomevalonate kinase; MDD, mevalonate diphosphate decarboxylase; FPS, farnesyl diphosphate synthase; IPPI, isopentenylpyrophosphate isomerase; DXS, DXP synthase; DXR, DXP reductoisomerase; MCT, MEP cytidylytransferase; CMK, CDP-ME kinase; MCS, MEC synthase; HDS, HMBPP synthase; HDR, HMBPP reductase.

**Figure 5:**
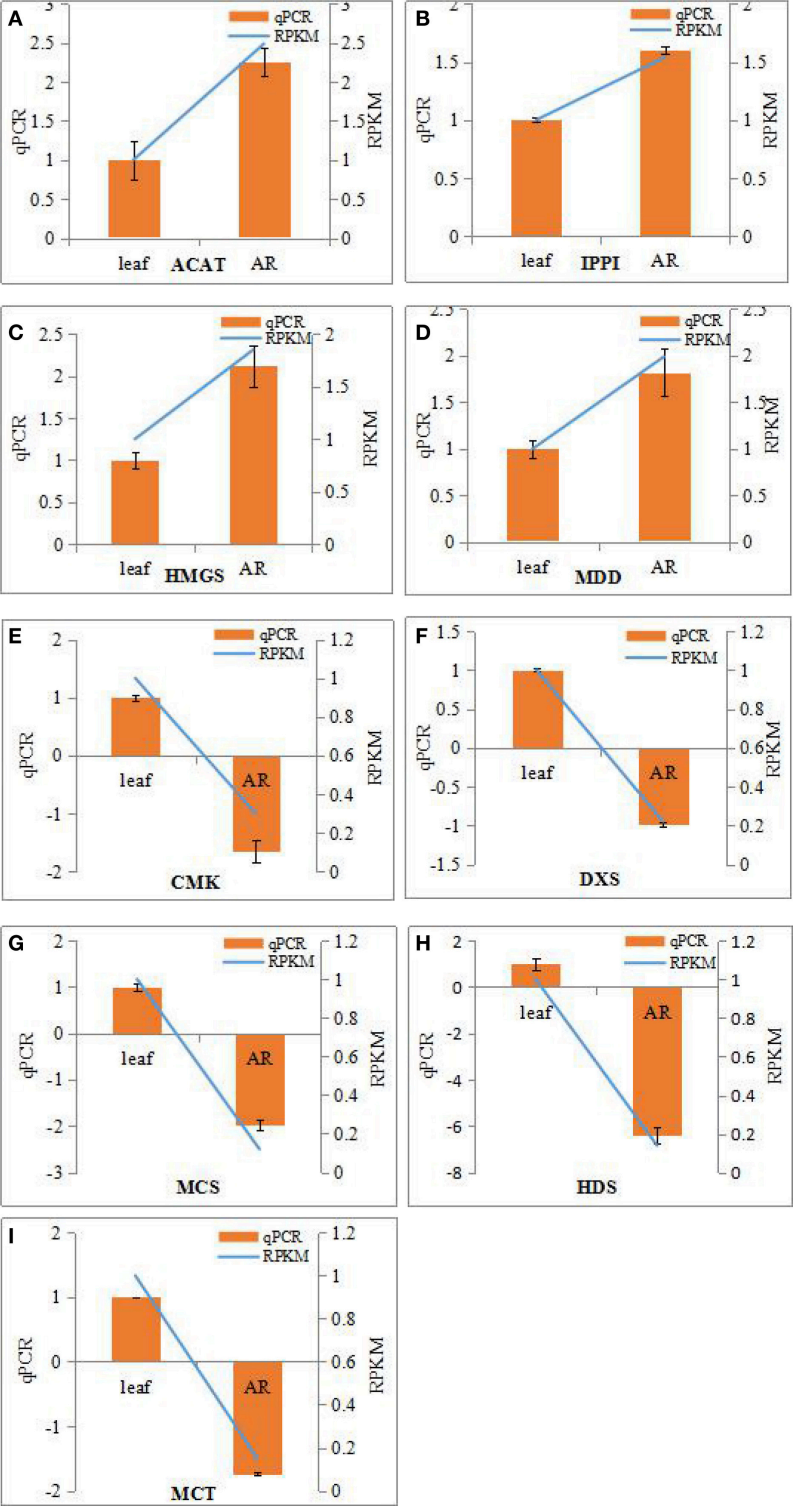
**Real-time PCR analysis of differentially expressed genes (DEGs) involved in IPP biosynthesis from MVA and MEP pathways in the adventitious roots and leaves of *Periploca sepium***. Real-time PCR analysis of ACAT **(A)**, IPPI **(B)**, HMGS **(C)**, MDD **(D)**, CMK **(E)**, DXS **(F)**, MCS **(G)**, HDS **(H)**, MCT **(I)**. The genes selected for validation were RNA-seq data. The left vertical axis showed relative expression levels as measured by qPCR values while the right vertical axis showed relative RPKM value. Experiments were conducted in triplicate. Error values for qPCR data at each sample time indicate standard deviation (SD).

The bioactive steroids were synthesized by a terpenoid backbone biosynthesis (ko00900) followed by a steroid biosynthesis (ko00100); their end products were steroid derivatives, such as cardiac glycosides and C21 steroids. We focused on changes in the expression of genes involved in this upstream biosynthesis pathway (Figure 4). As shown in Table S13, most genes in the adventitious roots or calli that were involved in the MVA pathway had higher RPKM values, but all genes in the methylerythritol phosphate (MEP) pathway had lower RPKM values. The DEGs analysis results showed that three genes relevant to the MVA pathway were up-regulated whereas five genes relevant to the MEP pathway were down-regulated in the adventitious roots. The up-regulated genes were ACAT, HMGS, and MDD; down-regulated genes were DXS, MEP cytidylytransferase (MCT), CDP-ME kinase (CMK), MEC synthase (MCS), and HMBPP synthase (HDS). The gene-encoding IPPI, an enzyme connecting the MVA and MEP pathways, also expressed an up-regulated response in the adventitious roots. Similar results were found in calli. Quantitative PCR (qPCR) analysis results showed that all nine selected DEGs in adventitious roots had the same expression pattern revealed by the differential analysis results from high-throughput sequencing (Figure 5).

However, the result for DEGs enriched in roots compared with leaves showed an unexpected outcome: no DEGs were enriched in the MEP pathway, yet some DEGs were unexpectedly and markedly down-regulated in the MVA pathway in roots (Table S13). An important gene in the MVA pathway encoding the key enzyme, HMGR, was included among these down-regulated genes.

### Expression characteristic of genes involved in the steroid biosynthesis pathway

**Figure 6:**
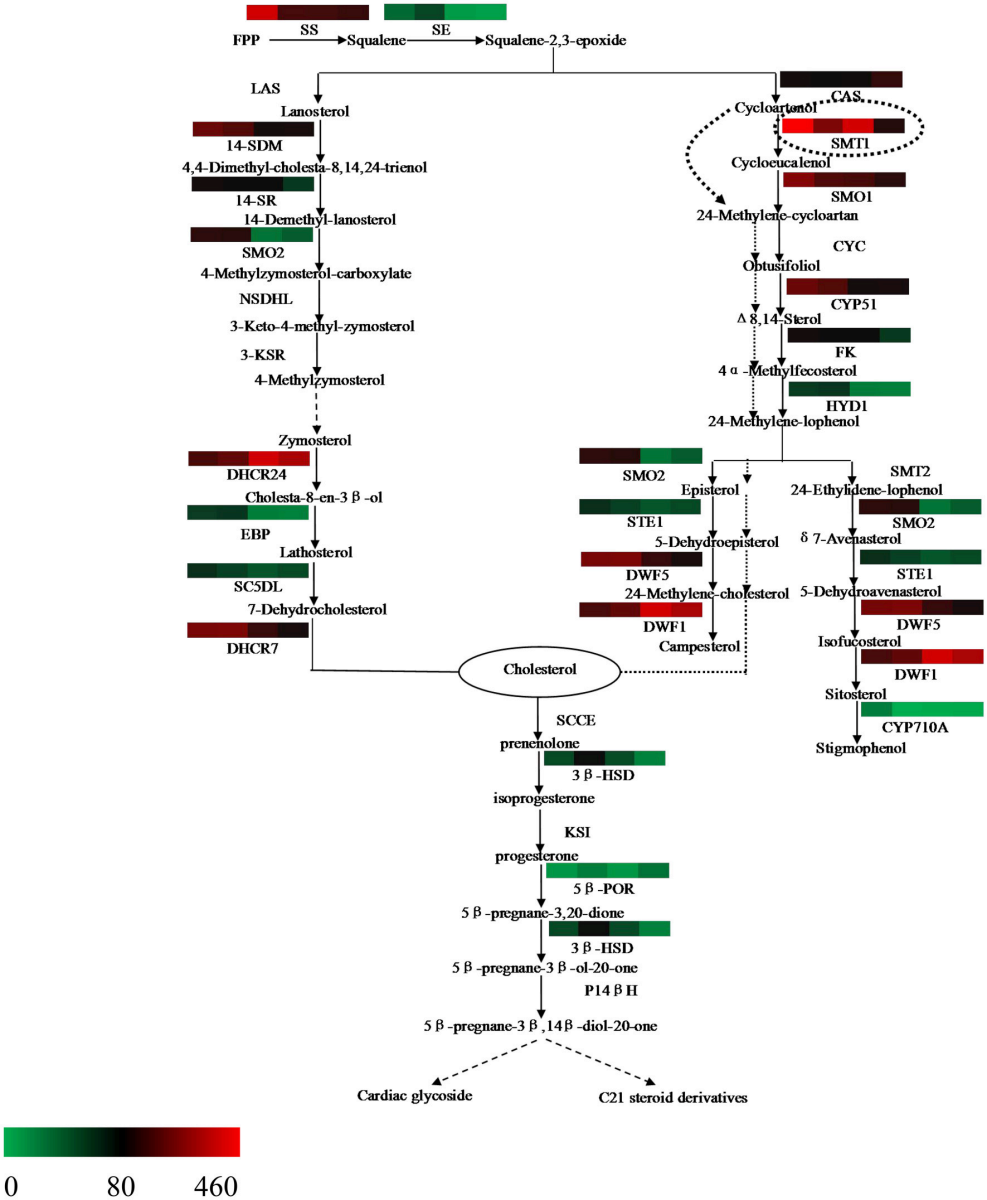
**Putative bioactive steroid biosynthesis in *Periploca sepium*.** Enzymes involved in bioactive steroid biosynthesis are shown. The expression of genes encoding these enzymes in AR, C, R, and L is shown by the heat-map. The genes were mapped using RPKM values and color coded by increasing relative expression. The broken arrow represents putative or unknown terminal biosynthesis steps for C21 steroids and cardiac glycoside. SS, squalene synthase; SE, squalene epoxidase; CAS, cycloartenol synthase; CYC, cycloeucalenol cycloisomerase; CYP51/14-SDM, sterol 14alpha-demethylase; CYP710A, sterol C-22 desaturase; DWF1/DHCR24, delta (24)-sterol reductase; DWF5/DHCR7, 7-dehydrocholesterol reductase; FK/14SR, delta(14)-sterol reductase; HYD1/EBP, C-8,7 sterol isomerase; LAS, lanosterol synthase; SMT, sterol C-24 methyltransferase; SMO, sterol-4alpha-methyl oxidase; STE1/SC5DL, delta(7)-sterol-C5(6)-desaturase; NSDHL, sterol-4alpha-carboxylate 3-dehydrogenase; 3-KSR,3-keto-steroid reductase; 3βHSD, 3-beta-hydroxysteroid dehydrogenase; 5βPOR, progesterone 5beta-reductase; SCCE, cholesterol monooxygenase, side chain-cleaving enzyme; KSI, delta 5-delta 4-ketosteroid isomerase; P14β, pregnane 14-beta hydroxylase.

The steroid biosynthesis pathway (ko00100) is the midstream synthetic pathway for plant bioactive steroids. In our study, many related genes that are involved in the steroid biosynthesis pathway were found (Table 4). The KEGG enrichment analysis showed that steroids could be biosynthesized via two pathways: the cycloartenol pathway and the lanosterol pathway (Figure 6). In the former, almost all of the participating genes were determined. In the lanosterol pathway some genes could not be identified, namely those encoding the enzymes of lanosterol synthase (LAS), sterol-4alpha-carboxylate 3-dehydrogenase (NSDHL), and 3-keto-steroid reductase (3-KSR).

**Table 4:** **Discovery of genes involved in the steroid biosynthesis pathway in *Periploca sepium***.

**Enzyme name**	**Abbreviation**	**EC number**	**Unique sequence**
**UPSTREAM GENES**			
acetyl-CoA acetyltransferase	ACAT	2.3.1.9	comp8784_c0_seq2
HMG-CoA synthase	HMGS	2.3.3.10	comp9104_c0_seq1
HMG-CoA reductase	HMGR	1.1.1.34	comp18428_c0_seq1
mevalonate kinase	MK	2.7.1.36	comp20801_c0_seq2
phosphomevalonate kinase	PMK	2.7.4.2	comp20246_c0_seq1
mevalonate diphosphate decarboxylase	MDD	4.1.1.33	comp16914_c0_seq1
isopentenylpyrophosphate isomerase	IPPI	5.3.3.2	comp21493_c0_seq1
farnesyl pyrophosphate synthase	FPS	2.5.1.10	comp14255_c0_seq1
**MIDSTREAM GENES**			
squalene synthase	SS	2.5.1.21	comp15171_c0_seq1
squalene epoxidase	SE	1.14.13.132	comp15489_c1_seq1
cycloartenol synthase	CAS	5.4.99.8	comp11904_c0_seq1
sterol C-24 methyltransferase	SMT1	2.1.1.41	comp12590_c0_seq1
sterol-4alpha-methyl oxidase 1	SMO1	1.14.13.72	comp15265_c0_seq1
cycloeucalenol cycloisomerase	CYC	5.5.1.9	comp13054_c0_seq1
sterol 14alpha-demethylase	CYP51/14-SDM	1.14.13.70	comp11403_c0_seq2
delta(14)-sterol reductase	FK/14SR	1.3.1.70	comp11605_c0_seq1
C-8,7 sterol isomerase	HYD1/EBP	5.3.3.5	comp7827_c0_seq1
sterol-4alpha-methyl oxidase 2	SMO2	1.14.13.72	comp14536_c0_seq1
delta(7)-sterol-C5(6)-desaturase	STE1/SC5DL	1.14.19.20	comp18083_c1_seq1
7-dehydrocholesterol reductase	DWF5/DHCR7	1.3.1.21	comp9124_c0_seq1
delta (24)-sterol reductase	DWF1/DHCR24	1.3.1.72	comp21254_c0_seq1
sterol 22-desaturase	CYP710A	1.14.19.41	comp10727_c0_seq1
**DOWNSTREAM GENES**			
progesterone 5beta-reductase	5βPOR	1.3.1.3	comp12025_c1_seq1
3-beta-hydroxysteroid dehydrogenase	3βHSD	1.1.1.145	comp9144_c0_seq1

Based on further analysis of these two metabolic pathways, it was apparent that most of the genes expressed higher RPKM values in the adventitious roots or calli compared with leaves. Many of these were significantly up-regulated genes, which included those encoding the enzymes of SS and SE, as well as those encoding the enzymes involved in the cycloartenol and lanosterol pathways, such as SMT1, SMO1, STE1/SC5DL, DHCR7/DWF5, CYP51/14-SDM, FK/14SR, HYD1, and others (Table S14). Further, we found that the gene encoding the enzyme of CAS expressed lower RPKM values in all three samples as compared with leaves, with a significantly down-regulated response in calli (Table S14).

Our analysis also revealed a general absence of up-regulated genes in roots; indeed, only a few genes were significantly up-regulated in roots (Table S14), a result similar to that for up-stream biosynthesis.

### Expression characteristics of genes involved in the C21 steroid and cardiac glycoside biosynthesis pathway

The terminal biosynthesis pathway of steroid metabolism includes the conversion of cholesterol into the C21 steroid and cardiac glycoside via a series of enzyme catalytic reactions (Figure 6). Until now, detailed knowledge of this pathway was rather limited. In the present study, two putative genes respectively encoding 3β-HSD and 5β-POR were found (Table 4). The differential gene analysis showed that the gene encoding 3βHSD expressed a significantly up-regulated response in all three samples compared with leaves. This result should contribute to a better understanding of the mechanism underpinning biosynthesis of C21 steroid and periplocin.

## Discussion

*Periploca sepium* Bunge is a well-known traditional medicinal plant. Until now, however, no studies have carried out the transcriptome sequencing of *P. sepium*. Even when considering the Asclepiadaceae family of plants as whole, reporting of their transcriptome or genome sequencing remains quite rare. This might change with the advent of Solexa, a next-generation sequencing technology, with the advantages of higher throughput, lower costs, and the fast generation of larger amounts of data (Morozova et al., 2009). For example, in the last 5 years, this technology has been successfully applied to the transcriptome sequencing of many plants, including *Populus euphratica* (Qiu et al., 2011), *Aegilops variabilis* (Xu et al., 2012), *Picrorhiza kurrooa* (Gahlan et al., 2012), *Panax notoginseng* (Luo et al., 2011), *Ammopiptanthus mongolicus* (Pang et al., 2013). To this list we may now add transcriptome sequencing of our four samples (L, R, AR, and C) of *P. sepium*, the first of it kind preformed using Solexa sequencing technology. Another advantage came from the Trinity software developed for *de novo* assembly: it can overcome the disadvantages associated with short reads, leading to the reliable assembly of transcriptome results lacking a reference genome (Grabherr et al., 2011).

Our prior studies and related research have shown that very little, if any, periplocin or C21 steroids occur in leaves of *P. sepium*, though many of the bioactive compounds exist both in the roots and in those tissue cultures induced by roots, such as adventitious roots and calli (Wei et al., 2009; Yin et al., 2009; Zhang et al., 2010a,b, 2011b; Wang et al., 2011). Hence, an analysis done of the different metabolic pathways related to bioactive steroid synthesis by comparing differences among the transcriptomes should help us better understand the workings of C21 steroid and periplocin biosynthesis and of related enzymes in *P. sepium*. The size of a transcriptome is influenced by both gene number and length (Pang et al., 2013). Our estimate of the nuclear genome size of *P. sepium* was approximately 170 Mb, while the total sequence nucleotides of the four sample reads was 4.64 Gb. This is the first quantification of the genome size of *P. sepium*.

### Analysis of the upstream biosynthesis pathway of bioactive steroids

Steroids are a class of hydrocarbons derived from the basic unit of isoprene. IPP and dimethylallyl diphosphate (DMAPP)—precursors of the isoprene compound—are biosynthesized by two separate pathways in plants: MVA and MEP (Zulak and Bohlmann, 2010). Prenyltransferase can catalyze IPP into geranyl diphosphate (GPP), while geranylgeranyl pyrophosphate (GGPP) and farnesyl pyrophosphate (FPP) can be further synthesized into various terpenoids and steroids. It is generally accepted that the MVA pathway provides the precursor of FPP, which can synthesize sesquiterpenoids, triterpenoids and steroids (Newman and Chappell, 1999), while the MEP pathway provides the precursor of GPP, which is related to monoterpenoid synthesis, and of GGPP, which is related to diterpenoid, phytol, gibberellin, and carotenoid synthesis (Lichtenthaler, 1999). Furthermore, previous studies have found that metabolism of the MVA pathway occurs primarily in the cytoplasm, whereas that of the MEP pathway happens mainly in the plastid (Wang et al., 2013). In the samples of leaves, adventitious roots, and calli, it is evident that the chloroplasts are common to leaves, but few are found in the adventitious roots or calli; this differentiation may explain why most of the genes in MEP pathway were up-regulated in leaves but some genes in MVA pathway were up-regulated in adventitious roots or calli. Nevertheless, this result may only partly explains why these active steroids mainly exist in the adventitious roots or calli induced by roots rather than in leaves. Such an explanation might shed light on the unexpected outcome of no up-regulated genes in MVA pathway and of no down-regulated genes in MEP pathway in the roots. In our experiment the sterile *P. sepium* seedlings were cultured in an MS medium without any activated carbon, allowing their roots to be stimulated under illumination that caused the taproots to become green during the growth process. This kind of root—one having chloroplasts—might change or adjust the synthetic route to get IPP, which could have lead to the result of IPP synthesis prevailing in the MEP instead of the MVA pathway.

In addition, we found that the gene encoding the rate-limiting enzyme HMGR failed to show any significantly up-regulated expression in the MVA pathway in either the adventitious roots or calli. This finding showed that the upstream metabolic pathway likely does not determine the synthesis ability of steroids in *P. sepium* seedlings. Instead, the obscure downstream synthesis pathway and its related key enzyme genes might have a more important influence on the biosynthesis of these bioactive steroids.

### Analysis of the downstream biosynthesis pathway of bioactive steroids

The steroid biosynthesis pathway begins with the formation of squalene catalyzed by SS, and only then can squalene be oxidized into squalene-2, 3-epoxide by SE. The catalytic reactions of SS and SE are the rate-limiting steps of the isoprene pathway. These are also the precursor synthesis pathways of steroids and triterpenoids. Much research shows that SS and SE, as important key enzymes, have a strong regulating function in the biosynthesis of steroids and triterpenoids (Choi et al., 2005; Seo et al., 2005; Han et al., 2010; Garaiová et al., 2014). Our present study revealed that these two enzyme genes in the adventitious roots and calli showed higher RPKM values (as compared with leaves). For both tissue samples, the genes encoding the enzymes of SS in adventitious roots and SE in adventitious roots and calli all expressed a remarkably up-regulated response.

Squalene-2,3-epoxide can be further cyclized to the cycloartenol or lanosterol precursors of plant steroids. The typical route of steroid biosynthesis pathway in plants follows the cycloartenol pathway for phytosterol biosynthesis, whereas the lanosterol pathway seems to be suitable principally for cholesterol biosynthesis in fungi and animals. However, it has been since demonstrated that both sterol pathways can operate in higher plants. Suzuki et al. (2006) identified the gene At3g45130 encoding the enzyme of LAS, lanosterol synthase, in *Arabidopsis thaliana*, followed by the gene found in *Panax ginseng* and *Lotus japonicus*, and others (Kolesnikova et al., 2006; Sawai et al., 2006). In the present study, the fact that both pathways of plant steroid biosynthesis were found in *P. sepium* lends further support to the view that higher plants do possess the lanosterol pathway (Figure 6). Nonetheless, some of the genes encoding the enzymes involved in the lanosterol pathway were not found in *P. sepium*, similar to results for *Marsdenia tenacissima* by Zheng et al. (2014). This discrepancy likely arose because the lanosterol pathway was discovered later in plants, hence some genes were only isolated and characterized in animals or fungus. In terms of plant steroid biosynthesis, one view is that the phytosterols, such as campesterol and sitosterol, are biosynthesized via the cycloartenol pathway, thus contributing to membrane sterol biosynthesis for primary metabolism. To this, the lanosterol pathway might contribute to the biosynthesis of bioactive steroids as secondary metabolites (Ohyama et al., 2009; Kim et al., 2013). Another view posits that the plants can synthesize cholesterol via the cycloartenol pathway (Diener et al., 2000; Holmberg et al., 2002). Considering their molecular structures, the difference between phytosterol and cholesterol is either a methyl or ethyl substitution at the carbon 24 (Diener et al., 2000). Diener et al. (2000) found that SMT1 played an important role in regulating plant steroid biosynthesis and then deduced the way of transformation from cycloartenol to cholesterol or phytosterol. In the present study, most of the genes encoding those enzymes involved in both the cycloartenol and lanosterol pathways expressed higher RPKM values, and over half of them were significantly up-regulated in adventitious roots or calli (Table S14). These up-regulated genes encode enzymes involved in several important biochemical reactions in steroid biosynthesis, such as demethylation at C-4, isomerization at C8-C7, and desaturation at C5, to name a few. Through a further analysis of the bioinformatics results, we found that while more genes related to the steroid biosynthesis pathway were up-regulated in the adventitious roots or calli, the gene encoding the enzyme of CAS expressed a lower RPKM value in all three samples. CAS is a isomerase that catalyzes the chemical reaction from squalene-2,3-epoxide to cycloartenol. Due to the down-regulation or lower RPKM value of this gene in the adventitious roots or calli, it was more likely that steroid metabolism would move toward the lanosterol pathway, which contributed to cholesterol synthesis.

Synthetic cholesterol can be further catalyzed to C21 steroids and cardiac glycoside. In plants the former are a class of steroid derivatives having 21 carbon atoms, but knowledge of these C21 steroids typically relies on the framework of pregnane or its isomer. The pregnane derivatives are intermediates in cardiac glycoside biosynthesis for which cholesterol is a direct precursor (Sauer et al., 1969; Lindemann and Luckner, 1997). Current studies of C21 steroid biosynthesis tend to focus on animal steroid hormone biosynthesis, but an understanding of the biosynthetic pathways for plant C21 steroids and cardiac glycoside is still very limited. Such a putative biosynthesis pathway in plants might involve several enzymes (Figure 6), including SCCE, 3βHSD, KSI, 5βPOR, P14βH, and possibly others too (Kreis and Müller-Ur, 2012; Zheng et al., 2014). The SCCE is commonly referred to as P450ssc, where “ssc” denotes a side-chain cleavage. P450scc is a mitochondrial enzyme that catalyzes conversion of cholesterol to pregnenolone: the first reaction in the process of C21 steroid biosynthesis (Hanukoglu, 1992). Pregnenolone can be further catalyzed to progesterone by 3βHSD and KSI. Following a series of biochemical reactions, progesterone is finally transformed into 5βPregnane-3β, 14βdiol-20-one, a possible precursor of the cardenolides (Finsterbusch et al., 1999; Lindemann, 2015). In the present study, we found two genes encoding the enzymes of 3βHSD and 5βPOR, respectively, but we did not find those encoding the enzymes of SCCE, KSI, and P14β because these are only isolated and characterized in animals or fungi (Zheng et al., 2014). Differential gene analysis showed that the gene encoding the enzyme of 3βHSD expressed a significantly up-regulated response in all three samples compared with leaves. The analysis of terminal metabolism from cholesterol to C21 steroid and cardiac glycoside should help to improve our understanding of the mechanism of C21 steroid and periplocin biosynthesis.

Finally, we also found very few up-regulated genes involved in the midstream and downstream steroid biosynthesis pathway in the roots compared with leaves, not unlike for up-stream biosynthesis. This finding further confirms that photosynthesis can change the secondary metabolism of steroids in the roots of *P. sepium* seedlings.

## Conclusions

In this study the genome size of *P. sepium* was determined. Leaf, root, adventitious root, and callus transcriptomes from *P. sepium* were sequenced for the first time. A large dataset of transcripts and unigenes provided abundant genetic information for discovering some important genes and secondary metabolic pathways. It was noteworthy that many genes involved in the upstream and midstream metabolic pathways of the C21 steroid and periplocin biosynthesis were identified. Moreover, some genes involved in an as-of-yet unclear downstream metabolic pathway were also found. These valuable gene candidates for the biosynthesis of the C21 steroid and periplocin could prove beneficial for producing larger quantities of such bioactive compounds for medical applications.

## Author contributions

XgL, FL, and JZ designed the research. JZ, XgL, FL, SW, YA, XS, XkL, LM, and GH performed the research. JZ and XgL wrote the paper.

## Accession numbers

Transcriptome sequence data described in the Short Read Archive database: SRR of calli, leaf, root, and adventitious root are 4835279, 4835278, 4835270, and 4835259, respectively.

### Conflict of interest statement

The authors declare that the research was conducted in the absence of any commercial or financial relationships that could be construed as a potential conflict of interest.
