# Supplementary material for: De novo Sequencing and Transcriptome Analysis Reveal Key Genes Regulating Steroid Metabolism in Leaves, Roots, Adventitious Roots and Calli of Periploca sepium Bunge
Source: Front Plant Sci. 2017 Apr 21;8:594. doi: 10.3389/fpls.2017.00594 (PMC5399629; doi:10.3389/fpls.2017.00594)
Supplement: Supplementary file 1 [file Table1.DOC]

**Table S1. Primers used in this study for real-time PCR.**

| **Enzyme** | **Transcript sequence** | **Forward primer sequence** | **Reverse primer sequence** |
| --- | --- | --- | --- |
| 18S | comp18442_c1_seq1 | CTTGTTGCTTGTCTCTCCT | GGCATTCCTTTAGCATAGTC |
| ACAT | comp8784_c0_seq2 | CGCAGCACAGACCATACA | AACCCATCTTTCATCAATCC |
| IPPI | comp21493_c0_seq1 | ACCTCTCATCATCGCAAAT | GTAATGGGTGTTGTAGTAGAAG |
| HMGS | comp9104_c0_seq1 | CAAGAGCAAGTCCATCAAG | ACACAACAAGACCATAACG |
| PMK | comp10276_c0_seq1 | CGTTCTTCCATCGCTCAA | CATCCTTGTATTCATCGTCATC |
| DXS | comp21095_c0_seq1 | CCACAGCCATTCCTAACC | GCCATCAGTCTTATCCTCAT |
| HDS | comp14296_c0_seq3 | ACGGAAACAGAAGGTGGG | ACTTTAGGGCGAAACCAA |
| MCS | comp16274_c0_seq1 | ATAGCATCCACAACACAGT | CTGCCATTACTCCGTCTAA |
| MCT | comp11638_c0_seq6 | GCATTTCCCAGAGTGTTT | TTAGCCGAGGATGTCAAG |
| MDD | comp16914_c0_seq1 | CCCAGACACCAACCAATA | TCCATTAAGCCACATACGA |
| SQS | comp15171_c0_seq1 | CGGTTGGATTGAATGAGATT | CTTCGGTTGCTCTAAGTCA |

*Note:* 18S rRNA was used as an internal control in real-time PCR system
