# Supplementary material for: De novo Sequencing and Transcriptome Analysis Reveal Key Genes Regulating Steroid Metabolism in Leaves, Roots, Adventitious Roots and Calli of Periploca sepium Bunge
Source: Front Plant Sci. 2017 Apr 21;8:594. doi: 10.3389/fpls.2017.00594 (PMC5399629; doi:10.3389/fpls.2017.00594)
Supplement: Supplementary file 2 [file Table2.DOC]

***Table S2. Discovery of genes annotated to Catharanthus roseus in the Nt database that are involved in the terpene indole alkaloid biosynthesis pathway in Periploca sepium.***

| **Enzymes name** | **Abbreviation** | **EC number** | **transcript** |
| --- | --- | --- | --- |
| **upstream genes** |  |  |  |
| acetyl-CoA acetyltransferase | ACAT | 2.3.1.9 | comp8784_c0_seq1  comp8784_c0_seq2 |
| HMG-CoA synthase | HMGS | 2.3.3.10 | comp9104_c0_seq1 |
| mevalonate kinase | MK | 2.7.1.36 | comp20801_c0_seq2 |
| phosphomevalonate kinase | PMK | 2.7.4.2 | comp20246_c0_seq1  comp20246_c0_seq2  comp20246_c0_seq3  comp20246_c0_seq4 |
| mevalonate diphosphate decarboxylase | MDD | 4.1.1.33 | comp16914_c0_seq1 |
| farnesyl pyrophosphate synthase | FPS | 2.5.1.10 | comp15141_c0_seq1  comp15141_c0_seq3  comp15141_c0_seq4  comp15141_c0_seq5 |
| DXP synthase | DXS | 2.2.1.7 | comp30603_c0_seq1  omp30714_c0_seq1  omp35727_c0_seq1  omp40044_c0_seq1 |
| geranyl diphosphate synthase | GPS | 2.5.1.1 | comp19172_c0_seq1  comp23208_c0_seq1  comp23285_c0_seq1  comp25366_c0_seq1  comp19379_c0_seq2 |
| **downstream genes** |  |  |  |
| 10-hydroxygeraniol oxidoreductase | 10-HGO | 1.14.14.1 | comp11599_c0_seq1  comp11599_c0_seq2  comp11599_c0_seq3  omp20784_c0_seq1  omp32548_c0_seq1 |
| desacetoxyvindoline 4-hydroxylase | D4H | 1.14.11.20 | comp11385_c0_seq1 |
| 16-hydroxytabersonine O-methyltransferase | 16-OMT | 2.1.1.94 | comp12899_c0_seq1 |
