# Supplementary material for: De novo Sequencing and Transcriptome Analysis Reveal Key Genes Regulating Steroid Metabolism in Leaves, Roots, Adventitious Roots and Calli of Periploca sepium Bunge
Source: Front Plant Sci. 2017 Apr 21;8:594. doi: 10.3389/fpls.2017.00594 (PMC5399629; doi:10.3389/fpls.2017.00594)
Supplement: Supplementary file 3 [file Table3.DOC]

**Table S3. Statistics of DEGs in AR, C, and R compared with L.**

| method | Up | | | Down | | | All | | |
| --- | --- | --- | --- | --- | --- | --- | --- | --- | --- |
|  | AR | C | R | AR | C | R | AR | C | R |
| MARS | 2604 | 2801 | 2325 | 4221 | 4253 | 3207 | 6825 | 7054 | 5532 |
| LRT | 1851 | 2302 | 1685 | 3094 | 2768 | 1685 | 4945 | 5070 | 3370 |
| FET | 1723 | 2134 | 1509 | 2676 | 2507 | 1850 | 4399 | 4641 | 3359 |
| FC | 5498 | 7457 | 6562 | 11291 | 8860 | 10023 | 16789 | 16317 | 16585 |
| intersection^1^ | 1723 | 2132 | 1508 | 2675 | 2507 | 1850 | 4398 | 4639 | 3358 |

^1^Differentially expressed genes were selected with the conditions of *p*-value ≤ 0.05 and |log2(fold change)| > 1
