# Supplementary material for: De novo Sequencing and Transcriptome Analysis Reveal Key Genes Regulating Steroid Metabolism in Leaves, Roots, Adventitious Roots and Calli of Periploca sepium Bunge
Source: Front Plant Sci. 2017 Apr 21;8:594. doi: 10.3389/fpls.2017.00594 (PMC5399629; doi:10.3389/fpls.2017.00594)
Supplement: Supplementary file 4 [file Table4.DOC]

**Table S4. Statistical analysis for the GO enrichment of AR vs L (*p* ≤ 0.05).**

| **GO-ID** | **Term** | **Category** | **Test** | **Ref** | **P-Value** |
| --- | --- | --- | --- | --- | --- |
| GO:0044710 | single-organism metabolic process | P | 139 | 879 | 9.88E-12 |
| GO:0016491 | oxidoreductase activity | F | 75 | 365 | 1.92E-10 |
| GO:0055114 | oxidation-reduction process | P | 72 | 351 | 5.27E-10 |
| GO:0020037 | heme binding | F | 22 | 60 | 4.54E-07 |
| GO:0046906 | tetrapyrrole binding | F | 24 | 75 | 1.03E-06 |
| GO:0016053 | organic acid biosynthetic process | P | 39 | 173 | 1.13E-06 |
| GO:0046394 | carboxylic acid biosynthetic process | P | 39 | 173 | 1.13E-06 |
| GO:0016209 | antioxidant activity | F | 15 | 29 | 1.18E-06 |
| GO:0009536 | plastid | C | 45 | 220 | 1.66E-06 |
| GO:0044283 | small molecule biosynthetic process | P | 42 | 200 | 2.13E-06 |
| GO:0044711 | single-organism biosynthetic process | P | 43 | 209 | 2.54E-06 |
| GO:0009507 | chloroplast | C | 43 | 209 | 2.54E-06 |
| GO:0016684 | oxidoreductase activity, acting on peroxide as acceptor | F | 13 | 24 | 4.23E-06 |
| GO:0004601 | peroxidase activity | F | 13 | 24 | 4.23E-06 |
| GO:0016705 | oxidoreductase activity, acting on paired donors, with incorporation or reduction of molecular oxygen | F | 20 | 61 | 6.25E-06 |
| GO:0005506 | iron ion binding | F | 20 | 68 | 2.37E-05 |
| GO:0004497 | monooxygenase activity | F | 13 | 30 | 2.73E-05 |
| GO:0009058 | biosynthetic process | P | 115 | 890 | 4.34E-05 |
| GO:0016702 | oxidoreductase activity, acting on single donors with incorporation of molecular oxygen, incorporation of two atoms of oxygen | F | 10 | 18 | 4.78E-05 |
| GO:0016701 | oxidoreductase activity, acting on single donors with incorporation of molecular oxygen | F | 10 | 18 | 4.78E-05 |
| GO:0019752 | carboxylic acid metabolic process | P | 46 | 268 | 7.09E-05 |
| GO:0071555 | cell wall organization | P | 9 | 15 | 7.42E-05 |
| GO:0045229 | external encapsulating structure organization | P | 9 | 15 | 7.42E-05 |
| GO:0043169 | cation binding | F | 78 | 551 | 8.69E-05 |
| GO:0006082 | organic acid metabolic process | P | 46 | 271 | 9.00E-05 |
| GO:0043436 | oxoacid metabolic process | P | 46 | 271 | 9.00E-05 |
| GO:0009664 | plant-type cell wall organization | P | 6 | 5 | 1.04E-04 |
| GO:0072330 | monocarboxylic acid biosynthetic process | P | 18 | 65 | 1.17E-04 |
| GO:0051213 | dioxygenase activity | F | 13 | 36 | 1.24E-04 |
| GO:0016765 | transferase activity, transferring alkyl or aryl (other than methyl) groups | F | 10 | 21 | 1.29E-04 |
| GO:0046872 | metal ion binding | F | 77 | 549 | 1.30E-04 |
| GO:0044249 | cellular biosynthetic process | P | 108 | 847 | 1.44E-04 |
| GO:0004478 | methionine adenosyltransferase activity | F | 6 | 6 | 1.93E-04 |
| GO:0006556 | S-adenosylmethionine biosynthetic process | P | 6 | 6 | 1.93E-04 |
| GO:0046500 | S-adenosylmethionine metabolic process | P | 6 | 6 | 1.93E-04 |
| GO:0008610 | lipid biosynthetic process | P | 25 | 116 | 1.95E-04 |
| GO:0008152 | metabolic process | P | 234 | 2208 | 2.08E-04 |
| GO:0003824 | catalytic activity | F | 195 | 1768 | 2.20E-04 |
| GO:0009055 | electron carrier activity | F | 18 | 71 | 2.98E-04 |
| GO:1901362 | organic cyclic compound biosynthetic process | P | 53 | 348 | 3.11E-04 |
| GO:1901576 | organic substance biosynthetic process | P | 108 | 865 | 3.16E-04 |
| GO:0019438 | aromatic compound biosynthetic process | P | 51 | 332 | 3.41E-04 |
| GO:0031408 | oxylipin biosynthetic process | P | 7 | 11 | 3.74E-04 |
| GO:0031407 | oxylipin metabolic process | P | 7 | 11 | 3.74E-04 |
| GO:0008652 | cellular amino acid biosynthetic process | P | 23 | 108 | 4.08E-04 |
| GO:0016165 | linoleate 13S-lipoxygenase activity | F | 7 | 12 | 5.50E-04 |
| GO:0006629 | lipid metabolic process | P | 31 | 175 | 7.30E-04 |
| GO:0006520 | cellular amino acid metabolic process | P | 30 | 168 | 7.92E-04 |
| GO:0071669 | plant-type cell wall organization or biogenesis | P | 6 | 9 | 8.42E-04 |
| GO:0043167 | ion binding | F | 122 | 1031 | 8.56E-04 |
| GO:0050662 | coenzyme binding | F | 14 | 53 | 9.91E-04 |
| GO:0008299 | isoprenoid biosynthetic process | P | 15 | 60 | 1.07E-03 |
| GO:0048037 | cofactor binding | F | 18 | 81 | 1.14E-03 |
| GO:0006979 | response to oxidative stress | P | 14 | 54 | 1.16E-03 |
| GO:0044255 | cellular lipid metabolic process | P | 26 | 141 | 1.18E-03 |
| GO:0016717 | oxidoreductase activity, acting on paired donors, with oxidation of a pair of donors resulting in the reduction of molecular oxygen to two molecules of water | F | 5 | 6 | 1.19E-03 |
| GO:0032787 | monocarboxylic acid metabolic process | P | 22 | 111 | 1.25E-03 |
| GO:0009733 | response to auxin stimulus | P | 6 | 10 | 1.25E-03 |
| GO:0006720 | isoprenoid metabolic process | P | 15 | 62 | 1.41E-03 |
| GO:0044272 | sulfur compound biosynthetic process | P | 15 | 62 | 1.41E-03 |
| GO:0006790 | sulfur compound metabolic process | P | 16 | 69 | 1.45E-03 |
| GO:0051188 | cofactor biosynthetic process | P | 14 | 56 | 1.55E-03 |
| GO:0071365 | cellular response to auxin stimulus | P | 5 | 7 | 1.90E-03 |
| GO:0009734 | auxin mediated signaling pathway | P | 5 | 7 | 1.90E-03 |
| GO:1901566 | organonitrogen compound biosynthetic process | P | 35 | 221 | 1.96E-03 |
| GO:0043289 | apocarotenoid biosynthetic process | P | 3 | 1 | 2.16E-03 |
| GO:0043288 | apocarotenoid metabolic process | P | 3 | 1 | 2.16E-03 |
| GO:0006714 | sesquiterpenoid metabolic process | P | 3 | 1 | 2.16E-03 |
| GO:0016106 | sesquiterpenoid biosynthetic process | P | 3 | 1 | 2.16E-03 |
| GO:0009688 | abscisic acid biosynthetic process | P | 3 | 1 | 2.16E-03 |
| GO:0009687 | abscisic acid metabolic process | P | 3 | 1 | 2.16E-03 |
| GO:0009072 | aromatic amino acid family metabolic process | P | 9 | 27 | 2.18E-03 |
| GO:0044281 | small molecule metabolic process | P | 70 | 541 | 2.22E-03 |
| GO:0071554 | cell wall organization or biogenesis | P | 10 | 33 | 2.25E-03 |
| GO:0009579 | thylakoid | C | 16 | 73 | 2.39E-03 |
| GO:0015977 | carbon fixation | P | 4 | 4 | 2.54E-03 |
| GO:0009765 | photosynthesis, light harvesting | P | 4 | 4 | 2.54E-03 |
| GO:0006732 | coenzyme metabolic process | P | 14 | 61 | 3.08E-03 |
| GO:0046914 | transition metal ion binding | F | 41 | 282 | 3.42E-03 |
| GO:0016829 | lyase activity | F | 15 | 70 | 3.90E-03 |
| GO:0051186 | cofactor metabolic process | P | 19 | 100 | 4.00E-03 |
| GO:0006730 | one-carbon metabolic process | P | 5 | 9 | 4.17E-03 |
| GO:0030145 | manganese ion binding | F | 4 | 5 | 4.27E-03 |
| GO:0034357 | photosynthetic membrane | C | 12 | 50 | 4.40E-03 |
| GO:0016831 | carboxy-lyase activity | F | 6 | 14 | 4.55E-03 |
| GO:0051537 | 2 iron, 2 sulfur cluster binding | F | 3 | 2 | 5.06E-03 |
| GO:0042398 | cellular modified amino acid biosynthetic process | P | 7 | 20 | 5.41E-03 |
| GO:0045300 | acyl-[acyl-carrier-protein] desaturase activity | F | 2 | 0 | 6.94E-03 |
| GO:0004351 | glutamate decarboxylase activity | F | 2 | 0 | 6.94E-03 |
| GO:0003849 | 3-deoxy-7-phosphoheptulonate synthase activity | F | 2 | 0 | 6.94E-03 |
| GO:0008519 | ammonium transmembrane transporter activity | F | 2 | 0 | 6.94E-03 |
| GO:0043650 | dicarboxylic acid biosynthetic process | P | 2 | 0 | 6.94E-03 |
| GO:0080161 | auxin transmembrane transporter activity | F | 2 | 0 | 6.94E-03 |
| GO:0015101 | organic cation transmembrane transporter activity | F | 2 | 0 | 6.94E-03 |
| GO:0072488 | ammonium transmembrane transport | P | 2 | 0 | 6.94E-03 |
| GO:0015979 | photosynthesis | P | 15 | 76 | 7.51E-03 |
| GO:0003899 | DNA-directed RNA polymerase activity | F | 5 | 11 | 7.91E-03 |
| GO:0006575 | cellular modified amino acid metabolic process | P | 8 | 28 | 8.18E-03 |
| GO:0009108 | coenzyme biosynthetic process | P | 8 | 28 | 8.18E-03 |
| GO:0044271 | cellular nitrogen compound biosynthetic process | P | 42 | 308 | 8.42E-03 |
| GO:0044436 | thylakoid part | C | 12 | 56 | 9.43E-03 |
| GO:0006536 | glutamate metabolic process | P | 3 | 3 | 9.50E-03 |
| GO:0051540 | metal cluster binding | F | 6 | 17 | 9.58E-03 |
| GO:0051536 | iron-sulfur cluster binding | F | 6 | 17 | 9.58E-03 |
| GO:0034062 | RNA polymerase activity | F | 5 | 12 | 1.05E-02 |
| GO:0043094 | cellular metabolic compound salvage | P | 5 | 12 | 1.05E-02 |
| GO:0018130 | heterocycle biosynthetic process | P | 42 | 316 | 1.23E-02 |
| GO:1901605 | alpha-amino acid metabolic process | P | 18 | 106 | 1.35E-02 |
| GO:0009074 | aromatic amino acid family catabolic process | P | 4 | 8 | 1.37E-02 |
| GO:0009853 | photorespiration | P | 4 | 8 | 1.37E-02 |
| GO:0009073 | aromatic amino acid family biosynthetic process | P | 5 | 14 | 1.71E-02 |
| GO:0009064 | glutamine family amino acid metabolic process | P | 5 | 14 | 1.71E-02 |
| GO:0043648 | dicarboxylic acid metabolic process | P | 5 | 14 | 1.71E-02 |
| GO:0042651 | thylakoid membrane | C | 10 | 47 | 1.78E-02 |
| GO:0004673 | protein histidine kinase activity | F | 4 | 9 | 1.85E-02 |
| GO:0000155 | phosphorelay sensor kinase activity | F | 4 | 9 | 1.85E-02 |
| GO:0023014 | signal transduction by phosphorylation | P | 4 | 9 | 1.85E-02 |
| GO:0019253 | reductive pentose-phosphate cycle | P | 2 | 1 | 1.97E-02 |
| GO:0050486 | intramolecular transferase activity, transferring hydroxy groups | F | 2 | 1 | 1.97E-02 |
| GO:0008909 | isochorismate synthase activity | F | 2 | 1 | 1.97E-02 |
| GO:0004506 | squalene monooxygenase activity | F | 2 | 1 | 1.97E-02 |
| GO:0045275 | respiratory chain complex III | C | 2 | 1 | 1.97E-02 |
| GO:0005750 | mitochondrial respiratory chain complex III | C | 2 | 1 | 1.97E-02 |
| GO:0019685 | photosynthesis, dark reaction | P | 2 | 1 | 1.97E-02 |
| GO:0045735 | nutrient reservoir activity | F | 2 | 1 | 1.97E-02 |
| GO:0009533 | chloroplast stromal thylakoid | C | 2 | 1 | 1.97E-02 |
| GO:0046039 | GTP metabolic process | P | 10 | 48 | 2.00E-02 |
| GO:0019684 | photosynthesis, light reaction | P | 11 | 56 | 2.12E-02 |
| GO:0000023 | maltose metabolic process | P | 3 | 5 | 2.35E-02 |
| GO:0055044 | symplast | C | 4 | 10 | 2.43E-02 |
| GO:0051287 | NAD binding | F | 4 | 10 | 2.43E-02 |
| GO:0009627 | systemic acquired resistance | P | 4 | 10 | 2.43E-02 |
| GO:0016775 | phosphotransferase activity, nitrogenous group as acceptor | F | 4 | 10 | 2.43E-02 |
| GO:0009506 | plasmodesma | C | 4 | 10 | 2.43E-02 |
| GO:0046148 | pigment biosynthetic process | P | 8 | 36 | 2.67E-02 |
| GO:1901659 | glycosyl compound biosynthetic process | P | 11 | 59 | 2.86E-02 |
| GO:1901564 | organonitrogen compound metabolic process | P | 56 | 473 | 3.01E-02 |
| GO:0006721 | terpenoid metabolic process | P | 7 | 30 | 3.06E-02 |
| GO:0016114 | terpenoid biosynthetic process | P | 7 | 30 | 3.06E-02 |
| GO:0009163 | nucleoside biosynthetic process | P | 10 | 52 | 3.07E-02 |
| GO:0042455 | ribonucleoside biosynthetic process | P | 10 | 52 | 3.07E-02 |
| GO:0042451 | purine nucleoside biosynthetic process | P | 10 | 52 | 3.07E-02 |
| GO:0046129 | purine ribonucleoside biosynthetic process | P | 10 | 52 | 3.07E-02 |
| GO:0005488 | binding | F | 184 | 1821 | 3.16E-02 |
| GO:0034654 | nucleobase-containing compound biosynthetic process | P | 33 | 254 | 3.29E-02 |
| GO:0006563 | L-serine metabolic process | P | 3 | 6 | 3.30E-02 |
| GO:0018298 | protein-chromophore linkage | P | 3 | 6 | 3.30E-02 |
| GO:1901069 | guanosine-containing compound catabolic process | P | 9 | 46 | 3.62E-02 |
| GO:0006184 | GTP catabolic process | P | 9 | 46 | 3.62E-02 |
| GO:0009535 | chloroplast thylakoid membrane | C | 9 | 46 | 3.62E-02 |
| GO:0017006 | protein-tetrapyrrole linkage | P | 2 | 2 | 3.72E-02 |
| GO:0004396 | hexokinase activity | F | 2 | 2 | 3.72E-02 |
| GO:0009881 | photoreceptor activity | F | 2 | 2 | 3.72E-02 |
| GO:0009584 | detection of visible light | P | 2 | 2 | 3.72E-02 |
| GO:0010207 | photosystem II assembly | P | 5 | 18 | 3.77E-02 |
| GO:0009069 | serine family amino acid metabolic process | P | 8 | 39 | 3.82E-02 |
| GO:0006633 | fatty acid biosynthetic process | P | 9 | 47 | 4.01E-02 |
| GO:0055035 | plastid thylakoid membrane | C | 9 | 47 | 4.01E-02 |
| GO:0032259 | methylation | P | 9 | 47 | 4.01E-02 |
| GO:0016741 | transferase activity, transferring one-carbon groups | F | 9 | 47 | 4.01E-02 |
| GO:0046983 | protein dimerization activity | F | 12 | 71 | 4.07E-02 |
| GO:0003924 | GTPase activity | F | 9 | 48 | 4.42E-02 |
| GO:0016709 | oxidoreductase activity, acting on paired donors, with incorporation or reduction of molecular oxygen, NAD(P)H as one donor, and incorporation of one atom of oxygen | F | 3 | 7 | 4.44E-02 |
| GO:0015995 | chlorophyll biosynthetic process | P | 5 | 19 | 4.44E-02 |
| GO:0009070 | serine family amino acid biosynthetic process | P | 7 | 33 | 4.47E-02 |
| GO:0043623 | cellular protein complex assembly | P | 14 | 89 | 4.51E-02 |
| GO:0009814 | defense response, incompatible interaction | P | 4 | 13 | 4.74E-02 |

*Note:* The abbreviation of P, F, and C represent biological process, molecular function, and cellular component, respectively.
