# Supplementary material for: De novo Sequencing and Transcriptome Analysis Reveal Key Genes Regulating Steroid Metabolism in Leaves, Roots, Adventitious Roots and Calli of Periploca sepium Bunge
Source: Front Plant Sci. 2017 Apr 21;8:594. doi: 10.3389/fpls.2017.00594 (PMC5399629; doi:10.3389/fpls.2017.00594)
Supplement: Supplementary file 5 [file Table5.DOC]

**Table S5. Statistical analysis for the GO enrichment of C vs L (*p* ≤ 0.05).**

| **GO ID** | **Term** | **Category** | **Test** | **Ref** | **P-Value** |
| --- | --- | --- | --- | --- | --- |
| GO:0020037 | heme binding | F | 24 | 58 | 1.00E-07 |
| GO:0005840 | ribosome | C | 40 | 151 | 1.92E-07 |
| GO:0003735 | structural constituent of ribosome | F | 38 | 139 | 2.02E-07 |
| GO:0009058 | biosynthetic process | P | 132 | 873 | 3.01E-07 |
| GO:0046906 | tetrapyrrole binding | F | 26 | 73 | 3.21E-07 |
| GO:0016209 | antioxidant activity | F | 16 | 28 | 5.60E-07 |
| GO:0016684 | oxidoreductase activity, acting on peroxide as acceptor | F | 14 | 23 | 1.67E-06 |
| GO:0004601 | peroxidase activity | F | 14 | 23 | 1.67E-06 |
| GO:0044249 | cellular biosynthetic process | P | 124 | 831 | 1.72E-06 |
| GO:0044710 | single-organism metabolic process | P | 130 | 888 | 2.15E-06 |
| GO:0008152 | metabolic process | P | 261 | 2181 | 2.45E-06 |
| GO:1901576 | organic substance biosynthetic process | P | 125 | 848 | 2.77E-06 |
| GO:0055114 | oxidation-reduction process | P | 65 | 358 | 5.51E-06 |
| GO:0009853 | photorespiration | P | 7 | 5 | 2.47E-05 |
| GO:0030529 | ribonucleoprotein complex | C | 42 | 208 | 3.83E-05 |
| GO:0043094 | cellular metabolic compound salvage | P | 8 | 9 | 4.82E-05 |
| GO:0006412 | translation | P | 45 | 234 | 5.66E-05 |
| GO:0016491 | oxidoreductase activity | F | 62 | 378 | 1.45E-04 |
| GO:0005618 | cell wall | C | 9 | 17 | 2.82E-04 |
| GO:0009059 | macromolecule biosynthetic process | P | 75 | 498 | 3.06E-04 |
| GO:0030312 | external encapsulating structure | C | 9 | 18 | 3.90E-04 |
| GO:0034645 | cellular macromolecule biosynthetic process | P | 74 | 496 | 4.38E-04 |
| GO:0010050 | vegetative phase change | P | 3 | 0 | 7.32E-04 |
| GO:0045275 | respiratory chain complex III | C | 3 | 0 | 7.32E-04 |
| GO:0005750 | mitochondrial respiratory chain complex III | C | 3 | 0 | 7.32E-04 |
| GO:0048437 | floral organ development | P | 6 | 8 | 8.37E-04 |
| GO:0006979 | response to oxidative stress | P | 15 | 53 | 8.37E-04 |
| GO:0030880 | RNA polymerase complex | C | 4 | 2 | 8.49E-04 |
| GO:0016591 | DNA-directed RNA polymerase II, holoenzyme | C | 4 | 2 | 8.49E-04 |
| GO:0055029 | nuclear DNA-directed RNA polymerase complex | C | 4 | 2 | 8.49E-04 |
| GO:0000428 | DNA-directed RNA polymerase complex | C | 4 | 2 | 8.49E-04 |
| GO:0048046 | apoplast | C | 9 | 21 | 9.33E-04 |
| GO:0032259 | methylation | P | 13 | 43 | 1.12E-03 |
| GO:0004497 | monooxygenase activity | F | 11 | 32 | 1.14E-03 |
| GO:0006414 | translational elongation | P | 11 | 33 | 1.40E-03 |
| GO:0009507 | chloroplast | C | 37 | 215 | 1.69E-03 |
| GO:0009733 | response to auxin stimulus | P | 6 | 10 | 1.91E-03 |
| GO:0016705 | oxidoreductase activity, acting on paired donors, with incorporation or reduction of molecular oxygen | F | 16 | 65 | 1.98E-03 |
| GO:0009055 | electron carrier activity | F | 17 | 72 | 2.12E-03 |
| GO:0043289 | apocarotenoid biosynthetic process | P | 3 | 1 | 2.73E-03 |
| GO:0043288 | apocarotenoid metabolic process | P | 3 | 1 | 2.73E-03 |
| GO:0006714 | sesquiterpenoid metabolic process | P | 3 | 1 | 2.73E-03 |
| GO:0016106 | sesquiterpenoid biosynthetic process | P | 3 | 1 | 2.73E-03 |
| GO:0005665 | DNA-directed RNA polymerase II, core complex | C | 3 | 1 | 2.73E-03 |
| GO:0048440 | carpel development | P | 3 | 1 | 2.73E-03 |
| GO:0009688 | abscisic acid biosynthetic process | P | 3 | 1 | 2.73E-03 |
| GO:0009687 | abscisic acid metabolic process | P | 3 | 1 | 2.73E-03 |
| GO:0010467 | gene expression | P | 71 | 509 | 3.06E-03 |
| GO:0009908 | flower development | P | 8 | 21 | 3.23E-03 |
| GO:0009765 | photosynthesis, light harvesting | P | 4 | 4 | 3.42E-03 |
| GO:0016741 | transferase activity, transferring one-carbon groups | F | 12 | 44 | 3.53E-03 |
| GO:0044451 | nucleoplasm part | C | 5 | 8 | 4.09E-03 |
| GO:0009536 | plastid | C | 37 | 228 | 4.14E-03 |
| GO:0071555 | cell wall organization | P | 7 | 17 | 4.14E-03 |
| GO:0045229 | external encapsulating structure organization | P | 7 | 17 | 4.14E-03 |
| GO:0008168 | methyltransferase activity | F | 11 | 40 | 4.88E-03 |
| GO:0044237 | cellular metabolic process | P | 192 | 1669 | 5.05E-03 |
| GO:0044283 | small molecule biosynthetic process | P | 34 | 208 | 5.36E-03 |
| GO:0044711 | single-organism biosynthetic process | P | 35 | 217 | 5.72E-03 |
| GO:0055044 | symplast | C | 5 | 9 | 5.90E-03 |
| GO:0009506 | plasmodesma | C | 5 | 9 | 5.90E-03 |
| GO:0051537 | 2 iron, 2 sulfur cluster binding | F | 3 | 2 | 6.37E-03 |
| GO:0080129 | proteasome core complex assembly | P | 3 | 2 | 6.37E-03 |
| GO:0048467 | gynoecium development | P | 3 | 2 | 6.37E-03 |
| GO:0005198 | structural molecule activity | F | 43 | 285 | 6.62E-03 |
| GO:0048037 | cofactor binding | F | 17 | 82 | 6.67E-03 |
| GO:0032991 | macromolecular complex | C | 73 | 546 | 6.98E-03 |
| GO:0016053 | organic acid biosynthetic process | P | 30 | 182 | 7.88E-03 |
| GO:0046394 | carboxylic acid biosynthetic process | P | 30 | 182 | 7.88E-03 |
| GO:0010022 | meristem determinacy | P | 2 | 0 | 8.14E-03 |
| GO:0004351 | glutamate decarboxylase activity | F | 2 | 0 | 8.14E-03 |
| GO:0003854 | 3-beta-hydroxy-delta5-steroid dehydrogenase activity | F | 2 | 0 | 8.14E-03 |
| GO:0003849 | 3-deoxy-7-phosphoheptulonate synthase activity | F | 2 | 0 | 8.14E-03 |
| GO:0010582 | floral meristem determinacy | P | 2 | 0 | 8.14E-03 |
| GO:0042026 | protein refolding | P | 2 | 0 | 8.14E-03 |
| GO:0080161 | auxin transmembrane transporter activity | F | 2 | 0 | 8.14E-03 |
| GO:0035999 | tetrahydrofolate interconversion | P | 2 | 0 | 8.14E-03 |
| GO:0070013 | intracellular organelle lumen | C | 13 | 57 | 8.76E-03 |
| GO:0051188 | cofactor biosynthetic process | P | 13 | 57 | 8.76E-03 |
| GO:0048438 | floral whorl development | P | 4 | 6 | 8.86E-03 |
| GO:1901362 | organic cyclic compound biosynthetic process | P | 50 | 351 | 9.34E-03 |
| GO:0019438 | aromatic compound biosynthetic process | P | 48 | 335 | 9.86E-03 |
| GO:0043232 | intracellular non-membrane-bounded organelle | C | 51 | 364 | 1.14E-02 |
| GO:0043228 | non-membrane-bounded organelle | C | 51 | 364 | 1.14E-02 |
| GO:0043248 | proteasome assembly | P | 3 | 3 | 1.19E-02 |
| GO:0006536 | glutamate metabolic process | P | 3 | 3 | 1.19E-02 |
| GO:0048466 | androecium development | P | 3 | 3 | 1.19E-02 |
| GO:0048443 | stamen development | P | 3 | 3 | 1.19E-02 |
| GO:0009579 | thylakoid | C | 15 | 74 | 1.25E-02 |
| GO:0071554 | cell wall organization or biogenesis | P | 9 | 34 | 1.28E-02 |
| GO:0006575 | cellular modified amino acid metabolic process | P | 8 | 28 | 1.30E-02 |
| GO:0022626 | cytosolic ribosome | C | 8 | 28 | 1.30E-02 |
| GO:0051540 | metal cluster binding | F | 6 | 17 | 1.40E-02 |
| GO:0051536 | iron-sulfur cluster binding | F | 6 | 17 | 1.40E-02 |
| GO:0005654 | nucleoplasm | C | 5 | 12 | 1.45E-02 |
| GO:0015979 | photosynthesis | P | 15 | 76 | 1.52E-02 |
| GO:0050662 | coenzyme binding | F | 12 | 55 | 1.53E-02 |
| GO:0006732 | coenzyme metabolic process | P | 13 | 62 | 1.55E-02 |
| GO:0031981 | nuclear lumen | C | 10 | 42 | 1.61E-02 |
| GO:1901617 | organic hydroxy compound biosynthetic process | P | 8 | 30 | 1.80E-02 |
| GO:0004478 | methionine adenosyltransferase activity | F | 4 | 8 | 1.81E-02 |
| GO:0006556 | S-adenosylmethionine biosynthetic process | P | 4 | 8 | 1.81E-02 |
| GO:0071365 | cellular response to auxin stimulus | P | 4 | 8 | 1.81E-02 |
| GO:0046500 | S-adenosylmethionine metabolic process | P | 4 | 8 | 1.81E-02 |
| GO:0009734 | auxin mediated signaling pathway | P | 4 | 8 | 1.81E-02 |
| GO:0016765 | transferase activity, transferring alkyl or aryl (other than methyl) groups | F | 7 | 24 | 1.81E-02 |
| GO:0005911 | cell-cell junction | C | 5 | 13 | 1.87E-02 |
| GO:0051186 | cofactor metabolic process | P | 18 | 101 | 1.93E-02 |
| GO:0016762 | xyloglucan:xyloglucosyl transferase activity | F | 3 | 4 | 1.94E-02 |
| GO:1901566 | organonitrogen compound biosynthetic process | P | 33 | 223 | 2.10E-02 |
| GO:0034357 | photosynthetic membrane | C | 11 | 51 | 2.12E-02 |
| GO:0019253 | reductive pentose-phosphate cycle | P | 2 | 1 | 2.30E-02 |
| GO:0035670 | plant-type ovary development | P | 2 | 1 | 2.30E-02 |
| GO:0004506 | squalene monooxygenase activity | F | 2 | 1 | 2.30E-02 |
| GO:0046653 | tetrahydrofolate metabolic process | P | 2 | 1 | 2.30E-02 |
| GO:0019685 | photosynthesis, dark reaction | P | 2 | 1 | 2.30E-02 |
| GO:0048481 | ovule development | P | 2 | 1 | 2.30E-02 |
| GO:0033764 | steroid dehydrogenase activity, acting on the CH-OH group of donors, NAD or NADP as acceptor | F | 2 | 1 | 2.30E-02 |
| GO:0019321 | pentose metabolic process | P | 2 | 1 | 2.30E-02 |
| GO:0043233 | organelle lumen | C | 13 | 66 | 2.32E-02 |
| GO:0009064 | glutamine family amino acid metabolic process | P | 5 | 14 | 2.35E-02 |
| GO:0008652 | cellular amino acid biosynthetic process | P | 19 | 112 | 2.48E-02 |
| GO:0031974 | membrane-enclosed lumen | C | 13 | 68 | 2.80E-02 |
| GO:0015977 | carbon fixation | P | 3 | 5 | 2.90E-02 |
| GO:0016831 | carboxy-lyase activity | F | 5 | 15 | 2.91E-02 |
| GO:0042398 | cellular modified amino acid biosynthetic process | P | 6 | 21 | 3.02E-02 |
| GO:0044271 | cellular nitrogen compound biosynthetic process | P | 42 | 308 | 3.03E-02 |
| GO:0006730 | one-carbon metabolic process | P | 4 | 10 | 3.16E-02 |
| GO:0046039 | GTP metabolic process | P | 10 | 48 | 3.28E-02 |
| GO:0048367 | shoot system development | P | 11 | 56 | 3.58E-02 |
| GO:0006091 | generation of precursor metabolites and energy | P | 23 | 151 | 3.85E-02 |
| GO:0009108 | coenzyme biosynthetic process | P | 7 | 29 | 3.90E-02 |
| GO:0044436 | thylakoid part | C | 11 | 57 | 3.94E-02 |
| GO:0006790 | sulfur compound metabolic process | P | 13 | 72 | 3.98E-02 |
| GO:0044445 | cytosolic part | C | 8 | 36 | 4.06E-02 |
| GO:0006563 | L-serine metabolic process | P | 3 | 6 | 4.07E-02 |
| GO:0044272 | sulfur compound biosynthetic process | P | 12 | 65 | 4.16E-02 |
| GO:0018130 | heterocycle biosynthetic process | P | 42 | 316 | 4.20E-02 |
| GO:0046165 | alcohol biosynthetic process | P | 5 | 17 | 4.26E-02 |
| GO:0045330 | aspartyl esterase activity | F | 2 | 2 | 4.32E-02 |
| GO:0004396 | hexokinase activity | F | 2 | 2 | 4.32E-02 |
| GO:0006555 | methionine metabolic process | P | 2 | 2 | 4.32E-02 |
| GO:0004372 | glycine hydroxymethyltransferase activity | F | 2 | 2 | 4.32E-02 |
| GO:0009086 | methionine biosynthetic process | P | 2 | 2 | 4.32E-02 |
| GO:0044267 | cellular protein metabolic process | P | 76 | 629 | 4.59E-02 |
| GO:0009163 | nucleoside biosynthetic process | P | 10 | 52 | 4.91E-02 |
| GO:0042455 | ribonucleoside biosynthetic process | P | 10 | 52 | 4.91E-02 |
| GO:0042451 | purine nucleoside biosynthetic process | P | 10 | 52 | 4.91E-02 |
| GO:1901615 | organic hydroxy compound metabolic process | P | 10 | 52 | 4.91E-02 |
| GO:0046129 | purine ribonucleoside biosynthetic process | P | 10 | 52 | 4.91E-02 |

*Note:* The abbreviation of P, F, and C represent biological process, molecular function, and cellular component, respectively.
