# Supplementary material for: De novo Sequencing and Transcriptome Analysis Reveal Key Genes Regulating Steroid Metabolism in Leaves, Roots, Adventitious Roots and Calli of Periploca sepium Bunge
Source: Front Plant Sci. 2017 Apr 21;8:594. doi: 10.3389/fpls.2017.00594 (PMC5399629; doi:10.3389/fpls.2017.00594)
Supplement: Supplementary file 6 [file Table6.DOC]

**Table S6. Statistical analysis for GO the enrichment of R vs L (*p* ≤ 0.05).**

| **GO ID** | **GO annotation** | **Category** | **Test Ref** | | **P-Value** |
| --- | --- | --- | --- | --- | --- |
| GO:0046906 | tetrapyrrole binding | F | 22 | 77 | 4.72E-09 |
| GO:0020037 | heme binding | F | 19 | 63 | 2.73E-08 |
| GO:0055114 | oxidation-reduction process | P | 49 | 374 | 4.81E-08 |
| GO:0044710 | single-organism metabolic process | P | 88 | 930 | 1.27E-07 |
| GO:0016491 | oxidoreductase activity | F | 48 | 392 | 4.52E-07 |
| GO:0009579 | thylakoid | C | 17 | 72 | 2.83E-06 |
| GO:0015979 | photosynthesis | P | 17 | 74 | 3.90E-06 |
| GO:0009507 | chloroplast | C | 31 | 221 | 6.13E-06 |
| GO:0009536 | plastid | C | 32 | 233 | 6.36E-06 |
| GO:0016684 | oxidoreductase activity, acting on peroxide as acceptor | F | 10 | 27 | 1.43E-05 |
| GO:0004601 | peroxidase activity | F | 10 | 27 | 1.43E-05 |
| GO:0034357 | photosynthetic membrane | C | 13 | 49 | 1.52E-05 |
| GO:0015977 | carbon fixation | P | 5 | 3 | 1.98E-05 |
| GO:0044436 | thylakoid part | C | 13 | 55 | 4.33E-05 |
| GO:0016209 | antioxidant activity | F | 10 | 34 | 7.31E-05 |
| GO:0019438 | aromatic compound biosynthetic process | P | 38 | 345 | 8.13E-05 |
| GO:1901362 | organic cyclic compound biosynthetic process | P | 39 | 362 | 1.01E-04 |
| GO:0019253 | reductive pentose-phosphate cycle | P | 3 | 0 | 1.48E-04 |
| GO:0019685 | photosynthesis, dark reaction | P | 3 | 0 | 1.48E-04 |
| GO:0019684 | photosynthesis, light reaction | P | 12 | 55 | 1.66E-04 |
| GO:0050662 | coenzyme binding | F | 12 | 55 | 1.66E-04 |
| GO:0009765 | photosynthesis, light harvesting | P | 4 | 4 | 4.58E-04 |
| GO:0042651 | thylakoid membrane | C | 10 | 47 | 6.93E-04 |
| GO:0009733 | response to auxin stimulus | P | 5 | 11 | 1.09E-03 |
| GO:0006569 | tryptophan catabolic process | P | 3 | 2 | 1.36E-03 |
| GO:0042436 | indole-containing compound catabolic process | P | 3 | 2 | 1.36E-03 |
| GO:0046218 | indolalkylamine catabolic process | P | 3 | 2 | 1.36E-03 |
| GO:0009684 | indoleacetic acid biosynthetic process | P | 3 | 2 | 1.36E-03 |
| GO:0009683 | indoleacetic acid metabolic process | P | 3 | 2 | 1.36E-03 |
| GO:0004497 | monooxygenase activity | F | 8 | 35 | 1.61E-03 |
| GO:0005618 | cell wall | C | 6 | 20 | 1.97E-03 |
| GO:0048037 | cofactor binding | F | 13 | 86 | 1.97E-03 |
| GO:0009535 | chloroplast thylakoid membrane | C | 9 | 46 | 2.13E-03 |
| GO:0009055 | electron carrier activity | F | 12 | 77 | 2.33E-03 |
| GO:0009072 | aromatic amino acid family metabolic process | P | 7 | 29 | 2.41E-03 |
| GO:0030312 | external encapsulating structure | C | 6 | 21 | 2.42E-03 |
| GO:0055035 | plastid thylakoid membrane | C | 9 | 47 | 2.42E-03 |
| GO:0009310 | amine catabolic process | P | 3 | 3 | 2.62E-03 |
| GO:0042402 | cellular biogenic amine catabolic process | P | 3 | 3 | 2.62E-03 |
| GO:0008171 | O-methyltransferase activity | F | 3 | 3 | 2.62E-03 |
| GO:0044271 | cellular nitrogen compound biosynthetic process | P | 31 | 319 | 2.69E-03 |
| GO:0009074 | aromatic amino acid family catabolic process | P | 4 | 8 | 2.73E-03 |
| GO:0042446 | hormone biosynthetic process | P | 4 | 8 | 2.73E-03 |
| GO:0071365 | cellular response to auxin stimulus | P | 4 | 8 | 2.73E-03 |
| GO:0009734 | auxin mediated signaling pathway | P | 4 | 8 | 2.73E-03 |
| GO:0006979 | response to oxidative stress | P | 10 | 58 | 2.79E-03 |
| GO:0019203 | carbohydrate phosphatase activity | F | 2 | 0 | 2.81E-03 |
| GO:0050308 | sugar-phosphatase activity | F | 2 | 0 | 2.81E-03 |
| GO:0008519 | ammonium transmembrane transporter activity | F | 2 | 0 | 2.81E-03 |
| GO:0042132 | fructose 1,6-bisphosphate 1-phosphatase activity | F | 2 | 0 | 2.81E-03 |
| GO:0042026 | protein refolding | P | 2 | 0 | 2.81E-03 |
| GO:0080161 | auxin transmembrane transporter activity | F | 2 | 0 | 2.81E-03 |
| GO:0015101 | organic cation transmembrane transporter activity | F | 2 | 0 | 2.81E-03 |
| GO:0072488 | ammonium transmembrane transport | P | 2 | 0 | 2.81E-03 |
| GO:0016702 | oxidoreductase activity, acting on single donors with incorporation of molecular oxygen, incorporation of two atoms of oxygen | F | 6 | 22 | 2.94E-03 |
| GO:0016701 | oxidoreductase activity, acting on single donors with incorporation of molecular oxygen | F | 6 | 22 | 2.94E-03 |
| GO:0016705 | oxidoreductase activity, acting on paired donors, with incorporation or reduction of molecular oxygen | F | 11 | 70 | 3.32E-03 |
| GO:0019748 | secondary metabolic process | P | 6 | 23 | 3.55E-03 |
| GO:0009626 | plant-type hypersensitive response | P | 4 | 9 | 3.78E-03 |
| GO:0051213 | dioxygenase activity | F | 8 | 41 | 3.79E-03 |
| GO:0018130 | heterocycle biosynthetic process | P | 31 | 327 | 3.82E-03 |
| GO:0009521 | photosystem | C | 6 | 24 | 4.25E-03 |
| GO:0042435 | indole-containing compound biosynthetic process | P | 3 | 4 | 4.41E-03 |
| GO:0034754 | cellular hormone metabolic process | P | 3 | 4 | 4.41E-03 |
| GO:0006091 | generation of precursor metabolites and energy | P | 18 | 156 | 4.51E-03 |
| GO:0008152 | metabolic process | P | 148 | 2294 | 4.80E-03 |
| GO:0034050 | host programmed cell death induced by symbiont | P | 4 | 10 | 5.08E-03 |
| GO:0003824 | catalytic activity | F | 123 | 1840 | 5.16E-03 |
| GO:0008544 | epidermis development | P | 7 | 35 | 5.94E-03 |
| GO:0051540 | metal cluster binding | F | 5 | 18 | 6.18E-03 |
| GO:0051536 | iron-sulfur cluster binding | F | 5 | 18 | 6.18E-03 |
| GO:0010207 | photosystem II assembly | P | 5 | 18 | 6.18E-03 |
| GO:0005506 | iron ion binding | F | 11 | 77 | 6.32E-03 |
| GO:0042445 | hormone metabolic process | P | 4 | 11 | 6.64E-03 |
| GO:0044711 | single-organism biosynthetic process | P | 23 | 229 | 6.74E-03 |
| GO:0006586 | indolalkylamine metabolic process | P | 3 | 5 | 6.78E-03 |
| GO:0006568 | tryptophan metabolic process | P | 3 | 5 | 6.78E-03 |
| GO:0042430 | indole-containing compound metabolic process | P | 3 | 5 | 6.78E-03 |
| GO:0044434 | chloroplast part | C | 16 | 138 | 6.99E-03 |
| GO:0015995 | chlorophyll biosynthetic process | P | 5 | 19 | 7.48E-03 |
| GO:0010817 | regulation of hormone levels | P | 5 | 19 | 7.48E-03 |
| GO:0043588 | skin development | P | 7 | 37 | 7.71E-03 |
| GO:0046148 | pigment biosynthetic process | P | 7 | 37 | 7.71E-03 |
| GO:0019321 | pentose metabolic process | P | 2 | 1 | 8.13E-03 |
| GO:0009533 | chloroplast stromal thylakoid | C | 2 | 1 | 8.13E-03 |
| GO:0003899 | DNA-directed RNA polymerase activity | F | 4 | 12 | 8.50E-03 |
| GO:0045088 | regulation of innate immune response | P | 4 | 12 | 8.50E-03 |
| GO:0044435 | plastid part | C | 16 | 142 | 8.91E-03 |
| GO:0009755 | hormone-mediated signaling pathway | P | 6 | 29 | 9.30E-03 |
| GO:0051186 | cofactor metabolic process | P | 13 | 106 | 9.71E-03 |
| GO:0030145 | manganese ion binding | F | 3 | 6 | 9.78E-03 |
| GO:0009851 | auxin biosynthetic process | P | 3 | 6 | 9.78E-03 |
| GO:0032774 | RNA biosynthetic process | P | 19 | 184 | 1.04E-02 |
| GO:0006351 | transcription, DNA-dependent | P | 19 | 184 | 1.04E-02 |
| GO:0034062 | RNA polymerase activity | F | 4 | 13 | 1.07E-02 |
| GO:0006779 | porphyrin-containing compound biosynthetic process | P | 6 | 30 | 1.07E-02 |
| GO:0033014 | tetrapyrrole biosynthetic process | P | 6 | 30 | 1.07E-02 |
| GO:0009069 | serine family amino acid metabolic process | P | 7 | 40 | 1.11E-02 |
| GO:0072330 | monocarboxylic acid biosynthetic process | P | 10 | 73 | 1.17E-02 |
| GO:0031976 | plastid thylakoid | C | 9 | 62 | 1.20E-02 |
| GO:0009534 | chloroplast thylakoid | C | 9 | 62 | 1.20E-02 |
| GO:0031984 | organelle subcompartment | C | 9 | 63 | 1.31E-02 |
| GO:0042537 | benzene-containing compound metabolic process | P | 4 | 14 | 1.31E-02 |
| GO:0009522 | photosystem I | C | 4 | 14 | 1.31E-02 |
| GO:0009850 | auxin metabolic process | P | 3 | 7 | 1.34E-02 |
| GO:1901566 | organonitrogen compound biosynthetic process | P | 22 | 234 | 1.57E-02 |
| GO:0032870 | cellular response to hormone stimulus | P | 6 | 33 | 1.57E-02 |
| GO:0005665 | DNA-directed RNA polymerase II, core complex | C | 2 | 2 | 1.57E-02 |
| GO:0048440 | carpel development | P | 2 | 2 | 1.57E-02 |
| GO:0042440 | pigment metabolic process | P | 8 | 54 | 1.58E-02 |
| GO:0050776 | regulation of immune response | P | 4 | 15 | 1.60E-02 |
| GO:0016053 | organic acid biosynthetic process | P | 19 | 193 | 1.61E-02 |
| GO:0046394 | carboxylic acid biosynthetic process | P | 19 | 193 | 1.61E-02 |
| GO:0044283 | small molecule biosynthetic process | P | 21 | 221 | 1.63E-02 |
| GO:0019288 | isopentenyl diphosphate biosynthetic process, methylerythritol 4-phosphate pathway | P | 5 | 24 | 1.68E-02 |
| GO:0009240 | isopentenyl diphosphate biosynthetic process | P | 5 | 24 | 1.68E-02 |
| GO:0046490 | isopentenyl diphosphate metabolic process | P | 5 | 24 | 1.68E-02 |
| GO:0019682 | glyceraldehyde-3-phosphate metabolic process | P | 5 | 24 | 1.68E-02 |
| GO:0009070 | serine family amino acid biosynthetic process | P | 6 | 34 | 1.76E-02 |
| GO:0010363 | regulation of plant-type hypersensitive response | P | 3 | 8 | 1.78E-02 |
| GO:0050661 | NADP binding | F | 3 | 8 | 1.78E-02 |
| GO:0009058 | biosynthetic process | P | 67 | 938 | 1.82E-02 |
| GO:0044550 | secondary metabolite biosynthetic process | P | 4 | 16 | 1.91E-02 |
| GO:0048046 | apoplast | C | 5 | 25 | 1.94E-02 |
| GO:0005976 | polysaccharide metabolic process | P | 8 | 57 | 2.05E-02 |
| GO:0000097 | sulfur amino acid biosynthetic process | P | 6 | 36 | 2.21E-02 |
| GO:0008654 | phospholipid biosynthetic process | P | 6 | 36 | 2.21E-02 |
| GO:0006725 | cellular aromatic compound metabolic process | P | 57 | 784 | 2.26E-02 |
| GO:0006576 | cellular biogenic amine metabolic process | P | 4 | 17 | 2.27E-02 |
| GO:0044106 | cellular amine metabolic process | P | 4 | 17 | 2.27E-02 |
| GO:0010374 | stomatal complex development | P | 4 | 17 | 2.27E-02 |
| GO:0010103 | stomatal complex morphogenesis | P | 4 | 17 | 2.27E-02 |
| GO:0000096 | sulfur amino acid metabolic process | P | 6 | 37 | 2.46E-02 |
| GO:0051537 | 2 iron, 2 sulfur cluster binding | F | 2 | 3 | 2.52E-02 |
| GO:0009926 | auxin polar transport | P | 2 | 3 | 2.52E-02 |
| GO:0048467 | gynoecium development | P | 2 | 3 | 2.52E-02 |
| GO:0043481 | anthocyanin accumulation in tissues in response to UV light | P | 2 | 3 | 2.52E-02 |
| GO:0043480 | pigment accumulation in tissues | P | 2 | 3 | 2.52E-02 |
| GO:0043479 | pigment accumulation in tissues in response to UV light | P | 2 | 3 | 2.52E-02 |
| GO:0043478 | pigment accumulation in response to UV light | P | 2 | 3 | 2.52E-02 |
| GO:0043476 | pigment accumulation | P | 2 | 3 | 2.52E-02 |
| GO:0043473 | pigmentation | P | 2 | 3 | 2.52E-02 |
| GO:1901363 | heterocyclic compound binding | F | 76 | 1105 | 2.58E-02 |
| GO:0009308 | amine metabolic process | P | 4 | 18 | 2.66E-02 |
| GO:1901360 | organic cyclic compound metabolic process | P | 58 | 808 | 2.68E-02 |
| GO:0097159 | organic cyclic compound binding | F | 76 | 1109 | 2.79E-02 |
| GO:0006090 | pyruvate metabolic process | P | 5 | 28 | 2.84E-02 |
| GO:0009699 | phenylpropanoid biosynthetic process | P | 3 | 10 | 2.85E-02 |
| GO:0009697 | salicylic acid biosynthetic process | P | 3 | 10 | 2.85E-02 |
| GO:0009696 | salicylic acid metabolic process | P | 3 | 10 | 2.85E-02 |
| GO:0009523 | photosystem II | C | 3 | 10 | 2.85E-02 |
| GO:0009657 | plastid organization | P | 7 | 50 | 2.98E-02 |
| GO:1901605 | alpha-amino acid metabolic process | P | 12 | 112 | 3.05E-02 |
| GO:0051188 | cofactor biosynthetic process | P | 8 | 62 | 3.07E-02 |
| GO:0019904 | protein domain specific binding | F | 4 | 19 | 3.09E-02 |
| GO:0046039 | GTP metabolic process | P | 7 | 51 | 3.24E-02 |
| GO:0016070 | RNA metabolic process | P | 26 | 312 | 3.26E-02 |
| GO:0055044 | symplast | C | 3 | 11 | 3.48E-02 |
| GO:0048765 | root hair cell differentiation | P | 3 | 11 | 3.48E-02 |
| GO:0048764 | trichoblast maturation | P | 3 | 11 | 3.48E-02 |
| GO:0051287 | NAD binding | F | 3 | 11 | 3.48E-02 |
| GO:0009627 | systemic acquired resistance | P | 3 | 11 | 3.48E-02 |
| GO:0009506 | plasmodesma | C | 3 | 11 | 3.48E-02 |
| GO:0008610 | lipid biosynthetic process | P | 13 | 128 | 3.52E-02 |
| GO:0071555 | cell wall organization | P | 4 | 20 | 3.56E-02 |
| GO:0045229 | external encapsulating structure organization | P | 4 | 20 | 3.56E-02 |
| GO:0015994 | chlorophyll metabolic process | P | 5 | 30 | 3.56E-02 |
| GO:0030880 | RNA polymerase complex | C | 2 | 4 | 3.66E-02 |
| GO:0016591 | DNA-directed RNA polymerase II, holoenzyme | C | 2 | 4 | 3.66E-02 |
| GO:0048038 | quinone binding | F | 2 | 4 | 3.66E-02 |
| GO:0055029 | nuclear DNA-directed RNA polymerase complex | C | 2 | 4 | 3.66E-02 |
| GO:0060918 | auxin transport | P | 2 | 4 | 3.66E-02 |
| GO:0000428 | DNA-directed RNA polymerase complex | C | 2 | 4 | 3.66E-02 |
| GO:0016051 | carbohydrate biosynthetic process | P | 9 | 77 | 3.73E-02 |
| GO:1901607 | alpha-amino acid biosynthetic process | P | 9 | 78 | 3.97E-02 |
| GO:0006778 | porphyrin-containing compound metabolic process | P | 6 | 42 | 3.98E-02 |
| GO:0033013 | tetrapyrrole metabolic process | P | 6 | 42 | 3.98E-02 |
| GO:1901576 | organic substance biosynthetic process | P | 63 | 910 | 3.99E-02 |
| GO:0006952 | defense response | P | 10 | 91 | 4.03E-02 |
| GO:0009725 | response to hormone stimulus | P | 8 | 66 | 4.09E-02 |
| GO:0006364 | rRNA processing | P | 7 | 54 | 4.11E-02 |
| GO:0016072 | rRNA metabolic process | P | 7 | 54 | 4.11E-02 |
| GO:0019439 | aromatic compound catabolic process | P | 14 | 145 | 4.13E-02 |
| GO:0043169 | cation binding | F | 43 | 586 | 4.24E-02 |
| GO:0006732 | coenzyme metabolic process | P | 8 | 67 | 4.38E-02 |
| GO:0008299 | isoprenoid biosynthetic process | P | 8 | 67 | 4.38E-02 |
| GO:1901564 | organonitrogen compound metabolic process | P | 37 | 492 | 4.39E-02 |
| GO:0071705 | nitrogen compound transport | P | 5 | 32 | 4.40E-02 |
| GO:0043623 | cellular protein complex assembly | P | 10 | 93 | 4.52E-02 |
| GO:0031347 | regulation of defense response | P | 4 | 22 | 4.61E-02 |
| GO:0009063 | cellular amino acid catabolic process | P | 4 | 22 | 4.61E-02 |
| GO:1901606 | alpha-amino acid catabolic process | P | 4 | 22 | 4.61E-02 |
| GO:1901361 | organic cyclic compound catabolic process | P | 14 | 148 | 4.71E-02 |
| GO:0006644 | phospholipid metabolic process | P | 6 | 44 | 4.72E-02 |
| GO:0003677 | DNA binding | F | 17 | 191 | 4.80E-02 |
| GO:0032787 | monocarboxylic acid metabolic process | P | 12 | 121 | 4.84E-02 |
| GO:0009886 | post-embryonic morphogenesis | P | 5 | 33 | 4.85E-02 |
| GO:1901617 | organic hydroxy compound biosynthetic process | P | 5 | 33 | 4.85E-02 |
| GO:0019344 | cysteine biosynthetic process | P | 5 | 33 | 4.85E-02 |
| GO:0034654 | nucleobase-containing compound biosynthetic process | P | 22 | 265 | 4.91E-02 |
| GO:0015696 | ammonium transport | P | 2 | 5 | 4.94E-02 |
| GO:0030095 | chloroplast photosystem II | C | 2 | 5 | 4.94E-02 |
| GO:0016762 | xyloglucan:xyloglucosyl transferase activity | F | 2 | 5 | 4.94E-02 |
| GO:0010181 | FMN binding | F | 2 | 5 | 4.94E-02 |
| GO:0010054 | trichoblast differentiation | P | 3 | 13 | 4.96E-02 |
| GO:0010053 | root epidermal cell differentiation | P | 3 | 13 | 4.96E-02 |
| GO:0009698 | phenylpropanoid metabolic process | P | 3 | 13 | 4.96E-02 |
| GO:0006720 | isoprenoid metabolic process | P | 8 | 69 | 4.99E-02 |

*Note*:The abbreviation of P, F, and C represent biological process, molecular function, and cellular component, respectively.
