# Supplementary material for: De novo Sequencing and Transcriptome Analysis Reveal Key Genes Regulating Steroid Metabolism in Leaves, Roots, Adventitious Roots and Calli of Periploca sepium Bunge
Source: Front Plant Sci. 2017 Apr 21;8:594. doi: 10.3389/fpls.2017.00594 (PMC5399629; doi:10.3389/fpls.2017.00594)
Supplement: Supplementary file 7 [file Table7.DOC]

**Table S7. Statistical analysis of the top 20 terms for GO enrichment in AR vs L (*p* ≤ 0.05).**

| GO-ID | Term | Category | Genes with annotation | | *p-*value |
| --- | --- | --- | --- | --- | --- |
|  |  |  | DEGs All genes | |  |
| 0008152 | metabolic process | P | 234 | 2208 | 2.08E-04 |
| 0003824 | catalytic activity | F | 195 | 1768 | 2.20E-04 |
| 0005488 | binding | F | 184 | 1821 | 3.16E-02 |
| 0044710 | single-organism metabolic process | P | 139 | 879 | 9.88E-12 |
| 0043167 | ion binding | F | 122 | 1031 | 8.56E-04 |
| 0009058 | biosynthetic process | P | 115 | 890 | 4.34E-05 |
| 0044249 | cellular biosynthetic process | P | 108 | 847 | 1.44E-04 |
| 1901576 | organic substance biosynthetic process | P | 108 | 865 | 3.16E-04 |
| 0043169 | cation binding | F | 78 | 551 | 8.69E-05 |
| 0046872 | metal ion binding | F | 77 | 549 | 1.30E-04 |
| 0016491 | oxidoreductase activity | F | 75 | 365 | 1.92E-10 |
| 0055114 | oxidation-reduction process | P | 72 | 351 | 5.27E-10 |
| 0044281 | small molecule metabolic process | P | 70 | 541 | 2.22E-03 |
| 1901564 | organonitrogen compound metabolic process | P | 56 | 473 | 3.01E-02 |
| 1901362 | organic cyclic compound biosynthetic process | P | 53 | 348 | 3.11E-04 |
| 0019438 | aromatic compound biosynthetic process | P | 51 | 332 | 3.41E-04 |
| 0019752 | carboxylic acid metabolic process | P | 46 | 268 | 7.09E-05 |
| 0006082 | organic acid metabolic process | P | 46 | 271 | 9.00E-05 |
| 0043436 | oxoacid metabolic process | P | 46 | 271 | 9.00E-05 |
| 0009536 | plastid | C | 45 | 220 | 1.66E-06 |

*Note*: The abbreviation of P, F, and C represent biological process, molecular function, and cellular component, respectively.
