# Supplementary material for: De novo Sequencing and Transcriptome Analysis Reveal Key Genes Regulating Steroid Metabolism in Leaves, Roots, Adventitious Roots and Calli of Periploca sepium Bunge
Source: Front Plant Sci. 2017 Apr 21;8:594. doi: 10.3389/fpls.2017.00594 (PMC5399629; doi:10.3389/fpls.2017.00594)
Supplement: Supplementary file 8 [file Table8.DOC]

**Table S8. Statistical analysis of the top 20 terms for GO enrichment in C vs L (*p* ≤ 0.05).**

| GO-ID | Term | Category | Genes with annotation | | *p-*value |
| --- | --- | --- | --- | --- | --- |
|  |  |  | DEGs All genes | |  |
| 0008152 | metabolic process | P | 261 | 2181 | 2.45E-06 |
| 0044237 | cellular metabolic process | P | 192 | 1669 | 5.05E-03 |
| 0009058 | biosynthetic process | P | 132 | 873 | 3.01E-07 |
| 0044710 | single-organism metabolic process | P | 130 | 888 | 2.15E-06 |
| 1901576 | organic substance biosynthetic process | P | 125 | 848 | 2.77E-06 |
| 0044249 | cellular biosynthetic process | P | 124 | 831 | 1.72E-06 |
| 0044267 | cellular protein metabolic process | P | 76 | 629 | 4.59E-02 |
| 0009059 | macromolecule biosynthetic process | P | 75 | 498 | 3.06E-04 |
| 0034645 | cellular macromolecule biosynthetic process | P | 74 | 496 | 4.38E-04 |
| 0032991 | macromolecular complex | C | 73 | 546 | 6.98E-03 |
| 0010467 | gene expression | P | 71 | 509 | 3.06E-03 |
| 0055114 | oxidation-reduction process | P | 65 | 358 | 5.51E-06 |
| 0016491 | oxidoreductase activity | F | 62 | 378 | 1.45E-04 |
| 0043232 | intracellular non-membrane-bounded organelle | C | 51 | 364 | 1.14E-02 |
| 0043228 | non-membrane-bounded organelle | C | 51 | 364 | 1.14E-02 |
| 1901362 | organic cyclic compound biosynthetic process | P | 50 | 351 | 9.34E-03 |
| 0019438 | aromatic compound biosynthetic process | P | 48 | 335 | 9.86E-03 |
| 0006412 | translation | P | 45 | 234 | 5.66E-05 |
| 0005198 | structural molecule activity | F | 43 | 285 | 6.62E-03 |
| 0030529 | ribonucleoprotein complex | C | 42 | 208 | 3.83E-05 |

*Note*: The abbreviation of P, F, and C represent biological process, molecular function, and cellular component, respectively.
