# Supplementary material for: De novo Sequencing and Transcriptome Analysis Reveal Key Genes Regulating Steroid Metabolism in Leaves, Roots, Adventitious Roots and Calli of Periploca sepium Bunge
Source: Front Plant Sci. 2017 Apr 21;8:594. doi: 10.3389/fpls.2017.00594 (PMC5399629; doi:10.3389/fpls.2017.00594)
Supplement: Supplementary file 9 [file Table9.DOC]

**Table S9. Statistical analysis of the top 20 terms for GO enrichment in R vs L (*p* ≤ 0.05).**

| GO-ID | Term | Category | Genes with annotation | | *p-*value |
| --- | --- | --- | --- | --- | --- |
|  |  |  | DEGs All genes | |  |
| 0008152 | metabolic process | P | 148 | 2294 | 4.80E-03 |
| 0003824 | catalytic activity | F | 123 | 1840 | 5.16E-03 |
| 0044710 | single-organism metabolic process | P | 88 | 930 | 1.27E-07 |
| 1901363 | heterocyclic compound binding | F | 76 | 1105 | 2.58E-02 |
| 0097159 | organic cyclic compound binding | F | 76 | 1109 | 2.79E-02 |
| 0009058 | biosynthetic process | P | 67 | 938 | 1.82E-02 |
| 1901576 | organic substance biosynthetic process | P | 63 | 910 | 3.99E-02 |
| 1901360 | organic cyclic compound metabolic process | P | 58 | 808 | 2.68E-02 |
| 0006725 | cellular aromatic compound metabolic process | P | 57 | 784 | 2.26E-02 |
| 0055114 | oxidation-reduction process | P | 49 | 374 | 4.81E-08 |
| 0016491 | oxidoreductase activity | F | 48 | 392 | 4.52E-07 |
| 0043169 | cation binding | F | 43 | 586 | 4.24E-02 |
| 1901362 | organic cyclic compound biosynthetic process | P | 39 | 362 | 1.01E-04 |
| 0019438 | aromatic compound biosynthetic process | P | 38 | 345 | 8.13E-05 |
| 1901564 | organonitrogen compound metabolic process | P | 37 | 492 | 4.39E-02 |
| 0009536 | plastid | C | 32 | 233 | 6.36E-06 |
| 0009507 | chloroplast | C | 31 | 221 | 6.13E-06 |
| 0044271 | cellular nitrogen compound biosynthetic process | P | 31 | 319 | 2.69E-03 |
| 0018130 | heterocycle biosynthetic process | P | 31 | 327 | 3.82E-03 |
| 0016070 | RNA metabolic process | P | 26 | 312 | 3.26E-02 |

*Note*: The abbreviation of P, F, and C represent biological process, molecular function, and cellular component, respectively.
