# Supplementary material for: De novo Sequencing and Transcriptome Analysis Reveal Key Genes Regulating Steroid Metabolism in Leaves, Roots, Adventitious Roots and Calli of Periploca sepium Bunge
Source: Front Plant Sci. 2017 Apr 21;8:594. doi: 10.3389/fpls.2017.00594 (PMC5399629; doi:10.3389/fpls.2017.00594)
Supplement: Supplementary file 10 [file Table10.DOC]

**Table S10. Statistical analysis for the KEGG enrichment of AR vs L (*p* ≤ 0.05).**

| **ID** | **Term** | **Input number** | **Background number** | **P-Value** |
| --- | --- | --- | --- | --- |
| ko03010 | Ribosome | 103 | 478 | 1.14E-18 |
| ko00195 | Photosynthesis | 34 | 88 | 1.34E-12 |
| ko00940 | Phenylpropanoid biosynthesis | 53 | 236 | 7.20E-11 |
| ko00710 | Carbon fixation in photosynthetic organisms | 34 | 127 | 4.39E-09 |
| ko04075 | Plant hormone signal transduction | 57 | 307 | 6.81E-09 |
| ko01200 | Carbon metabolism | 79 | 522 | 3.49E-08 |
| ko00360 | Phenylalanine metabolism | 35 | 149 | 4.40E-08 |
| ko00270 | Cysteine and methionine metabolism | 29 | 142 | 6.69E-06 |
| ko00630 | Glyoxylate and dicarboxylate metabolism | 25 | 114 | 9.87E-06 |
| ko00010 | Glycolysis / Gluconeogenesis | 41 | 249 | 1.13E-05 |
| ko01230 | Biosynthesis of amino acids | 67 | 522 | 4.79E-05 |
| ko00941 | Flavonoid biosynthesis | 12 | 34 | 5.04E-05 |
| ko00592 | alpha-Linolenic acid metabolism | 16 | 60 | 5.40E-05 |
| ko00480 | Glutathione metabolism | 23 | 117 | 9.51E-05 |
| ko00100 | Steroid biosynthesis | 14 | 50 | 0.000100428 |
| ko00620 | Pyruvate metabolism | 27 | 153 | 0.000123094 |
| ko00030 | Pentose phosphate pathway | 18 | 80 | 0.000125066 |
| ko01040 | Biosynthesis of unsaturated fatty acids | 15 | 59 | 0.000143611 |
| ko00945 | Stilbenoid, diarylheptanoid and gingerol biosynthesis | 9 | 22 | 0.00018013 |
| ko00591 | Linoleic acid metabolism | 12 | 43 | 0.000317519 |
| ko00750 | Vitamin B6 metabolism | 6 | 11 | 0.000697942 |
| ko00860 | Porphyrin and chlorophyll metabolism | 20 | 111 | 0.000699442 |
| ko00909 | Sesquiterpenoid and triterpenoid biosynthesis | 8 | 22 | 0.000772146 |
| ko00196 | Photosynthesis - antenna proteins | 10 | 39 | 0.001693563 |
| ko00071 | Fatty acid degradation | 16 | 86 | 0.001719198 |
| ko00920 | Sulfur metabolism | 12 | 56 | 0.002287297 |
| ko00980 | Metabolism of xenobiotics by cytochrome P450 | 13 | 66 | 0.002920292 |
| ko00903 | Limonene and pinene degradation | 5 | 11 | 0.003635529 |
| ko00625 | Chloroalkane and chloroalkene degradation | 7 | 23 | 0.003750161 |
| ko00260 | Glycine, serine and threonine metabolism | 18 | 114 | 0.004461468 |
| ko00905 | Brassinosteroid biosynthesis | 6 | 20 | 0.007583007 |
| ko00910 | Nitrogen metabolism | 10 | 50 | 0.007782455 |
| ko00350 | Tyrosine metabolism | 12 | 67 | 0.007982425 |
| ko05204 | Chemical carcinogenesis | 11 | 61 | 0.010379974 |
| ko00982 | Drug metabolism - cytochrome P450 | 12 | 70 | 0.010678321 |
| ko00680 | Methane metabolism | 19 | 137 | 0.011791627 |
| ko00521 | Streptomycin biosynthesis | 4 | 10 | 0.013101306 |
| ko01220 | Degradation of aromatic compounds | 5 | 17 | 0.015493466 |
| ko00040 | Pentose and glucuronate interconversions | 16 | 112 | 0.015697178 |
| ko00900 | Terpenoid backbone biosynthesis | 16 | 114 | 0.017968801 |
| ko00430 | Taurine and hypotaurine metabolism | 5 | 19 | 0.022298729 |
| ko04918 | Thyroid hormone synthesis | 8 | 43 | 0.023144714 |
| ko02020 | Two-component system | 7 | 35 | 0.024125399 |
| ko04740 | Olfactory transduction | 4 | 13 | 0.026390252 |
| ko00450 | Selenocompound metabolism | 6 | 28 | 0.027652906 |
| ko01212 | Fatty acid metabolism | 18 | 142 | 0.028843256 |
| ko04146 | Peroxisome | 21 | 174 | 0.029780421 |
| ko00590 | Arachidonic acid metabolism | 5 | 21 | 0.030788555 |
| ko04744 | Phototransduction | 4 | 14 | 0.032116206 |
| ko00190 | Oxidative phosphorylation | 32 | 297 | 0.033477611 |
| ko04626 | Plant-pathogen interaction | 28 | 255 | 0.036877159 |
| ko00250 | Alanine, aspartate and glutamate metabolism | 14 | 106 | 0.0382652 |
| ko00020 | Citrate cycle (TCA cycle) | 13 | 98 | 0.04354389 |
| ko04612 | Antigen processing and presentation | 12 | 94 | 0.063041428 |
| ko00650 | Butanoate metabolism | 6 | 36 | 0.067811724 |
| ko00053 | Ascorbate and aldarate metabolism | 11 | 86 | 0.072213487 |
| ko00380 | Tryptophan metabolism | 10 | 76 | 0.073442189 |
| ko00830 | Retinol metabolism | 4 | 20 | 0.080486959 |
| ko00130 | Ubiquinone and other terpenoid-quinone biosynthesis | 10 | 79 | 0.087906768 |
| ko05016 | Huntington's disease | 26 | 259 | 0.092417439 |
| ko00720 | Carbon fixation pathways in prokaryotes | 8 | 60 | 0.09711636 |
| ko04112 | Cell cycle - Caulobacter | 5 | 31 | 0.100561917 |
| ko04971 | Gastric acid secretion | 4 | 22 | 0.101715122 |
| ko04932 | Non-alcoholic fatty liver disease (NAFLD) | 18 | 171 | 0.106182841 |
| ko00052 | Galactose metabolism | 14 | 126 | 0.106239555 |
| ko00906 | Carotenoid biosynthesis | 10 | 83 | 0.109675282 |
| ko04745 | Phototransduction - fly | 5 | 32 | 0.110004334 |
| ko00400 | Phenylalanine, tyrosine and tryptophan biosynthesis | 16 | 151 | 0.11759291 |
| ko00626 | Naphthalene degradation | 3 | 15 | 0.124506112 |
| ko00460 | Cyanoamino acid metabolism | 11 | 98 | 0.134051037 |
| ko00051 | Fructose and mannose metabolism | 11 | 99 | 0.140204762 |
| ko04978 | Mineral absorption | 4 | 26 | 0.150763615 |
| ko04970 | Salivary secretion | 4 | 26 | 0.150763615 |
| ko00520 | Amino sugar and nucleotide sugar metabolism | 24 | 253 | 0.152859446 |
| ko00362 | Benzoate degradation | 3 | 18 | 0.174750198 |
| ko03320 | PPAR signaling pathway | 7 | 60 | 0.180574447 |
| ko05130 | Pathogenic Escherichia coli infection | 10 | 95 | 0.191100264 |
| ko00760 | Nicotinate and nicotinamide metabolism | 4 | 29 | 0.192329709 |
| ko00950 | Isoquinoline alkaloid biosynthesis | 4 | 29 | 0.192329709 |
| ko04145 | Phagosome | 19 | 202 | 0.195035784 |
| ko05144 | Malaria | 2 | 10 | 0.200034513 |
| ko00471 | D-Glutamine and D-glutamate metabolism | 2 | 10 | 0.200034513 |
| ko04976 | Bile secretion | 8 | 74 | 0.206800223 |
| ko00904 | Diterpenoid biosynthesis | 3 | 20 | 0.210866695 |
| ko00230 | Purine metabolism | 25 | 284 | 0.237947536 |
| ko00960 | Tropane, piperidine and pyridine alkaloid biosynthesis | 5 | 44 | 0.250450245 |
| ko00061 | Fatty acid biosynthesis | 7 | 68 | 0.262217488 |
| ko03060 | Protein export | 10 | 104 | 0.26543328 |
| ko04915 | Estrogen signaling pathway | 16 | 179 | 0.277334315 |
| ko00902 | Monoterpenoid biosynthesis | 2 | 13 | 0.278962606 |
| ko04144 | Endocytosis | 19 | 217 | 0.281379273 |
| ko03020 | RNA polymerase | 8 | 83 | 0.294890064 |
| ko04727 | GABAergic synapse | 7 | 71 | 0.295210183 |
| ko04066 | HIF-1 signaling pathway | 16 | 183 | 0.304805742 |
| ko00565 | Ether lipid metabolism | 8 | 85 | 0.315626007 |
| ko04064 | NF-kappa B signaling pathway | 8 | 85 | 0.315626007 |
| ko05012 | Parkinson's disease | 18 | 210 | 0.317030399 |
| ko00072 | Synthesis and degradation of ketone bodies | 2 | 15 | 0.331473576 |
| ko05322 | Systemic lupus erythematosus | 5 | 50 | 0.332574686 |
| ko04080 | Neuroactive ligand-receptor interaction | 1 | 5 | 0.349966384 |
| ko00524 | Butirosin and neomycin biosynthesis | 1 | 5 | 0.349966384 |
| ko05143 | African trypanosomiasis | 2 | 16 | 0.357344509 |
| ko00330 | Arginine and proline metabolism | 15 | 178 | 0.360341788 |
| ko00730 | Thiamine metabolism | 3 | 28 | 0.364475941 |
| ko04014 | Ras signaling pathway | 15 | 179 | 0.367861965 |
| ko00908 | Zeatin biosynthesis | 8 | 90 | 0.368503937 |
| ko04750 | Inflammatory mediator regulation of TRP channels | 4 | 41 | 0.379845035 |
| ko04912 | GnRH signaling pathway | 13 | 155 | 0.380934466 |
| ko00660 | C5-Branched dibasic acid metabolism | 1 | 6 | 0.394999608 |
| ko00627 | Aminobenzoate degradation | 2 | 18 | 0.407888881 |
| ko04011 | MAPK signaling pathway - yeast | 2 | 18 | 0.407888881 |
| ko00073 | Cutin, suberine and wax biosynthesis | 2 | 18 | 0.407888881 |
| ko04972 | Pancreatic secretion | 5 | 57 | 0.430559052 |
| ko00240 | Pyrimidine metabolism | 19 | 240 | 0.431894007 |
| ko04975 | Fat digestion and absorption | 2 | 19 | 0.432429795 |
| ko02010 | ABC transporters | 9 | 109 | 0.432520928 |
| ko00401 | Novobiocin biosynthesis | 1 | 7 | 0.436914042 |
| ko04910 | Insulin signaling pathway | 20 | 256 | 0.450510884 |
| ko04973 | Carbohydrate digestion and absorption | 2 | 20 | 0.456413206 |
| ko05133 | Pertussis | 14 | 178 | 0.461055646 |
| ko00670 | One carbon pool by folate | 5 | 60 | 0.471830658 |
| ko04540 | Gap junction | 12 | 153 | 0.473730986 |
| ko04712 | Circadian rhythm - plant | 10 | 127 | 0.479206481 |
| ko04722 | Neurotrophin signaling pathway | 18 | 234 | 0.481423344 |
| ko04070 | Phosphatidylinositol signaling system | 10 | 129 | 0.497822807 |
| ko00340 | Histidine metabolism | 4 | 49 | 0.504963266 |
| ko00062 | Fatty acid elongation | 4 | 49 | 0.504963266 |
| ko04711 | Circadian rhythm - fly | 1 | 9 | 0.512235283 |
| ko00780 | Biotin metabolism | 3 | 36 | 0.512978211 |
| ko05206 | MicroRNAs in cancer | 15 | 199 | 0.519435557 |
| ko00500 | Starch and sucrose metabolism | 37 | 497 | 0.52390558 |
| ko05164 | Influenza A | 19 | 254 | 0.525585612 |
| ko00561 | Glycerolipid metabolism | 10 | 135 | 0.552462881 |
| ko05231 | Choline metabolism in cancer | 14 | 191 | 0.561419255 |
| ko04961 | Endocrine and other factor-regulated calcium reabsorption | 3 | 39 | 0.563824408 |
| ko04330 | Notch signaling pathway | 2 | 25 | 0.566819885 |
| ko05162 | Measles | 11 | 151 | 0.571693182 |
| ko04930 | Type II diabetes mellitus | 8 | 110 | 0.577004598 |
| ko00140 | Steroid hormone biosynthesis | 1 | 11 | 0.577484282 |
| ko04916 | Melanogenesis | 10 | 138 | 0.578907023 |
| ko04015 | Rap1 signaling pathway | 12 | 167 | 0.589089996 |
| ko05142 | Chagas disease (American trypanosomiasis) | 13 | 182 | 0.597515935 |
| ko05020 | Prion diseases | 9 | 127 | 0.605007416 |
| ko05230 | Central carbon metabolism in cancer | 14 | 197 | 0.605396308 |
| ko04964 | Proximal tubule bicarbonate reclamation | 2 | 27 | 0.606214066 |
| ko04623 | Cytosolic DNA-sensing pathway | 3 | 42 | 0.611289873 |
| ko05010 | Alzheimer's disease | 29 | 407 | 0.617273016 |
| ko04260 | Cardiac muscle contraction | 4 | 58 | 0.63044308 |
| ko04911 | Insulin secretion | 1 | 13 | 0.634007521 |
| ko04630 | Jak-STAT signaling pathway | 1 | 13 | 0.634007521 |
| ko05214 | Glioma | 9 | 132 | 0.648185443 |
| ko04152 | AMPK signaling pathway | 11 | 161 | 0.651481874 |
| ko05215 | Prostate cancer | 11 | 161 | 0.651481874 |
| ko04024 | cAMP signaling pathway | 15 | 218 | 0.652958623 |
| ko04920 | Adipocytokine signaling pathway | 4 | 61 | 0.66741774 |
| ko05416 | Viral myocarditis | 3 | 46 | 0.668963347 |
| ko04141 | Protein processing in endoplasmic reticulum | 31 | 447 | 0.674706374 |
| ko00310 | Lysine degradation | 5 | 77 | 0.679024894 |
| ko03070 | Bacterial secretion system | 3 | 47 | 0.682359523 |
| ko04713 | Circadian entrainment | 7 | 107 | 0.682717872 |
| ko04621 | NOD-like receptor signaling pathway | 7 | 107 | 0.682717872 |
| ko00290 | Valine, leucine and isoleucine biosynthesis | 1 | 15 | 0.682971537 |
| ko05217 | Basal cell carcinoma | 1 | 15 | 0.682971537 |
| ko01210 | 2-Oxocarboxylic acid metabolism | 6 | 93 | 0.689908504 |
| ko04919 | Thyroid hormone signaling pathway | 13 | 196 | 0.695568753 |
| ko04620 | Toll-like receptor signaling pathway | 12 | 183 | 0.705202286 |
| ko05145 | Toxoplasmosis | 14 | 212 | 0.705407479 |
| ko00562 | Inositol phosphate metabolism | 11 | 174 | 0.74169489 |
| ko00983 | Drug metabolism - other enzymes | 2 | 36 | 0.749924784 |
| ko04962 | Vasopressin-regulated water reabsorption | 3 | 54 | 0.764876604 |
| ko05140 | Leishmaniasis | 10 | 163 | 0.766102031 |
| ko00790 | Folate biosynthesis | 3 | 55 | 0.775112793 |
| ko00300 | Lysine biosynthesis | 1 | 20 | 0.778614332 |
| ko04270 | Vascular smooth muscle contraction | 9 | 150 | 0.779295554 |
| ko04728 | Dopaminergic synapse | 5 | 89 | 0.787883964 |
| ko04974 | Protein digestion and absorption | 2 | 40 | 0.797786485 |
| ko04670 | Leukocyte transendothelial migration | 2 | 40 | 0.797786485 |
| ko04391 | Hippo signaling pathway - fly | 3 | 58 | 0.803637523 |
| ko05034 | Alcoholism | 13 | 216 | 0.807819556 |
| ko04340 | Hedgehog signaling pathway | 1 | 22 | 0.808237182 |
| ko00740 | Riboflavin metabolism | 1 | 22 | 0.808237182 |
| ko04666 | Fc gamma R-mediated phagocytosis | 12 | 201 | 0.808593925 |
| ko04122 | Sulfur relay system | 2 | 42 | 0.818529817 |
| ko00410 | beta-Alanine metabolism | 8 | 142 | 0.823024172 |
| ko00564 | Glycerophospholipid metabolism | 13 | 221 | 0.830496677 |
| ko05211 | Renal cell carcinoma | 7 | 131 | 0.849406203 |
| ko05110 | Vibrio cholerae infection | 5 | 100 | 0.86021296 |
| ko04111 | Cell cycle - yeast | 9 | 168 | 0.870905637 |
| ko05203 | Viral carcinogenesis | 16 | 278 | 0.871337088 |
| ko05132 | Salmonella infection | 8 | 154 | 0.879911382 |
| ko05412 | Arrhythmogenic right ventricular cardiomyopathy (ARVC) | 1 | 29 | 0.884017793 |
| ko04012 | ErbB signaling pathway | 6 | 122 | 0.885115628 |
| ko05410 | Hypertrophic cardiomyopathy (HCM) | 2 | 52 | 0.896132357 |
| ko05414 | Dilated cardiomyopathy | 1 | 31 | 0.899540301 |
| ko00603 | Glycosphingolipid biosynthesis - globo series | 1 | 32 | 0.906504453 |
| ko04062 | Chemokine signaling pathway | 6 | 129 | 0.912291413 |
| ko05221 | Acute myeloid leukemia | 5 | 112 | 0.91430888 |
| ko04390 | Hippo signaling pathway | 4 | 94 | 0.914555799 |
| ko05146 | Amoebiasis | 1 | 34 | 0.919018348 |
| ko04724 | Glutamatergic synapse | 13 | 248 | 0.919514137 |
| ko04068 | FoxO signaling pathway | 11 | 216 | 0.920848135 |
| ko04917 | Prolactin signaling pathway | 5 | 115 | 0.924553497 |
| ko05222 | Small cell lung cancer | 1 | 35 | 0.924632644 |
| ko04710 | Circadian rhythm | 1 | 36 | 0.92985784 |
| ko04550 | Signaling pathways regulating pluripotency of stem cells | 5 | 117 | 0.930764494 |
| ko05161 | Hepatitis B | 7 | 153 | 0.932123704 |
| ko05014 | Amyotrophic lateral sclerosis (ALS) | 7 | 153 | 0.932123704 |
| ko04664 | Fc epsilon RI signaling pathway | 5 | 118 | 0.933695435 |
| ko00970 | Aminoacyl-tRNA biosynthesis | 9 | 188 | 0.933737105 |
| ko05200 | Pathways in cancer | 14 | 271 | 0.93418962 |
| ko05202 | Transcriptional misregulation in cancer | 3 | 81 | 0.936290538 |
| ko04151 | PI3K-Akt signaling pathway | 14 | 277 | 0.945025135 |
| ko04013 | MAPK signaling pathway - fly | 4 | 105 | 0.948938173 |
| ko04914 | Progesterone-mediated oocyte maturation | 8 | 178 | 0.949047569 |
| ko00280 | Valine, leucine and isoleucine degradation | 9 | 196 | 0.950186586 |
| ko04150 | mTOR signaling pathway | 6 | 143 | 0.950341049 |
| ko00770 | Pantothenate and CoA biosynthesis | 1 | 41 | 0.951028164 |
| ko05213 | Endometrial cancer | 5 | 125 | 0.95127932 |
| ko04320 | Dorso-ventral axis formation | 4 | 107 | 0.953619783 |
| ko04520 | Adherens junction | 6 | 145 | 0.954349598 |
| ko03050 | Proteasome | 3 | 88 | 0.955836439 |
| ko04810 | Regulation of actin cytoskeleton | 11 | 234 | 0.956266701 |
| ko05100 | Bacterial invasion of epithelial cells | 2 | 67 | 0.956797458 |
| ko04725 | Cholinergic synapse | 4 | 109 | 0.957902912 |
| ko05216 | Thyroid cancer | 4 | 110 | 0.959904501 |
| ko05212 | Pancreatic cancer | 5 | 131 | 0.962851933 |
| ko03030 | DNA replication | 4 | 112 | 0.963645674 |
| ko04668 | TNF signaling pathway | 4 | 112 | 0.963645674 |
| ko04210 | Apoptosis | 8 | 187 | 0.963958014 |
| ko04071 | Sphingolipid signaling pathway | 10 | 223 | 0.964886605 |
| ko04726 | Serotonergic synapse | 4 | 115 | 0.968653002 |
| ko05218 | Melanoma | 4 | 116 | 0.970173674 |
| ko05223 | Non-small cell lung cancer | 4 | 116 | 0.970173674 |
| ko05219 | Bladder cancer | 4 | 116 | 0.970173674 |
| ko05131 | Shigellosis | 7 | 174 | 0.970758106 |
| ko04730 | Long-term depression | 4 | 117 | 0.971625137 |
| ko05210 | Colorectal cancer | 7 | 175 | 0.971955858 |
| ko05220 | Chronic myeloid leukemia | 4 | 118 | 0.973010243 |
| ko04261 | Adrenergic signaling in cardiomyocytes | 7 | 177 | 0.97421745 |
| ko04960 | Aldosterone-regulated sodium reabsorption | 3 | 98 | 0.974220473 |
| ko04110 | Cell cycle | 9 | 214 | 0.974677363 |
| ko04940 | Type I diabetes mellitus | 3 | 99 | 0.975592637 |
| ko05323 | Rheumatoid arthritis | 1 | 51 | 0.976131734 |
| ko05134 | Legionellosis | 7 | 181 | 0.978245443 |
| ko05169 | Epstein-Barr virus infection | 11 | 261 | 0.983432918 |
| ko04350 | TGF-beta signaling pathway | 4 | 128 | 0.983772115 |
| ko04723 | Retrograde endocannabinoid signaling | 3 | 107 | 0.984322457 |
| ko04020 | Calcium signaling pathway | 6 | 170 | 0.984883348 |
| ko05120 | Epithelial cell signaling in Helicobacter pylori infection | 1 | 58 | 0.985569368 |
| ko05160 | Hepatitis C | 4 | 131 | 0.986107388 |
| ko03008 | Ribosome biogenesis in eukaryotes | 6 | 175 | 0.988009239 |
| ko04142 | Lysosome | 6 | 180 | 0.990518731 |
| ko00640 | Propanoate metabolism | 4 | 140 | 0.991343739 |
| ko05152 | Tuberculosis | 21 | 459 | 0.992669356 |
| ko03440 | Homologous recombination | 2 | 97 | 0.993186502 |
| ko05205 | Proteoglycans in cancer | 8 | 227 | 0.993233203 |
| ko04921 | Oxytocin signaling pathway | 13 | 321 | 0.993286835 |
| ko04611 | Platelet activation | 4 | 146 | 0.993719102 |
| ko05031 | Amphetamine addiction | 4 | 154 | 0.995929798 |
| ko05166 | HTLV-I infection | 10 | 281 | 0.996500435 |
| ko04140 | Regulation of autophagy | 1 | 79 | 0.996812551 |
| ko04720 | Long-term potentiation | 9 | 264 | 0.996866634 |
| ko03013 | RNA transport | 11 | 305 | 0.997138102 |
| ko04510 | Focal adhesion | 7 | 227 | 0.997324705 |
| ko04530 | Tight junction | 1 | 83 | 0.997609544 |
| ko00513 | Various types of N-glycan biosynthesis | 2 | 118 | 0.998215729 |
| ko04010 | MAPK signaling pathway | 8 | 264 | 0.998769545 |
| ko03018 | RNA degradation | 12 | 345 | 0.998807848 |
| ko05168 | Herpes simplex infection | 5 | 200 | 0.998842113 |
| ko04721 | Synaptic vesicle cycle | 1 | 94 | 0.998916652 |
| ko04115 | p53 signaling pathway | 3 | 153 | 0.998926335 |
| ko04380 | Osteoclast differentiation | 6 | 228 | 0.999131568 |
| ko00510 | N-Glycan biosynthesis | 3 | 159 | 0.999253022 |
| ko03410 | Base excision repair | 2 | 133 | 0.999325873 |
| ko04662 | B cell receptor signaling pathway | 6 | 235 | 0.999397773 |
| ko04114 | Oocyte meiosis | 12 | 363 | 0.999453741 |
| ko04130 | SNARE interactions in vesicular transport | 1 | 111 | 0.999681298 |
| ko04022 | cGMP-PKG signaling pathway | 8 | 293 | 0.999702217 |
| ko04650 | Natural killer cell mediated cytotoxicity | 5 | 228 | 0.999751424 |
| ko03022 | Basal transcription factors | 1 | 116 | 0.999777643 |
| ko04660 | T cell receptor signaling pathway | 5 | 230 | 0.999777769 |
| ko04360 | Axon guidance | 5 | 230 | 0.999777769 |
| ko04370 | VEGF signaling pathway | 5 | 237 | 0.999850161 |
| ko04310 | Wnt signaling pathway | 3 | 188 | 0.999874599 |
| ko04120 | Ubiquitin mediated proteolysis | 7 | 289 | 0.999884019 |
| ko03420 | Nucleotide excision repair | 2 | 171 | 0.999945194 |
| ko03015 | mRNA surveillance pathway | 3 | 215 | 0.999977075 |
| ko04113 | Meiosis - yeast | 6 | 301 | 0.999983773 |
| ko03460 | Fanconi anemia pathway | 1 | 153 | 0.999984528 |
| ko03040 | Spliceosome | 14 | 496 | 0.999991946 |
