# Supplementary material for: De novo Sequencing and Transcriptome Analysis Reveal Key Genes Regulating Steroid Metabolism in Leaves, Roots, Adventitious Roots and Calli of Periploca sepium Bunge
Source: Front Plant Sci. 2017 Apr 21;8:594. doi: 10.3389/fpls.2017.00594 (PMC5399629; doi:10.3389/fpls.2017.00594)
Supplement: Supplementary file 11 [file Table11.DOC]

**Table S11. Statistical analysis for the KEGG enrichment of C vs L (*p* ≤ 0.05).**

| **ID** | **Term** | **Input number** | **Background number** | **P-Value** |
| --- | --- | --- | --- | --- |
| ko03010 | Ribosome | 174 | 478 | 1.02E-44 |
| ko00195 | Photosynthesis | 30 | 88 | 2.32E-08 |
| ko00710 | Carbon fixation in photosynthetic organisms | 31 | 127 | 5.98E-06 |
| ko00270 | Cysteine and methionine metabolism | 32 | 142 | 1.72E-05 |
| ko00360 | Phenylalanine metabolism | 33 | 149 | 1.73E-05 |
| ko00480 | Glutathione metabolism | 28 | 117 | 2.24E-05 |
| ko05016 | Huntington's disease | 47 | 259 | 3.37E-05 |
| ko00940 | Phenylpropanoid biosynthesis | 44 | 236 | 3.38E-05 |
| ko04075 | Plant hormone signal transduction | 53 | 307 | 3.63E-05 |
| ko00945 | Stilbenoid, diarylheptanoid and gingerol biosynthesis | 11 | 22 | 4.49E-05 |
| ko05322 | Systemic lupus erythematosus | 16 | 50 | 7.82E-05 |
| ko00941 | Flavonoid biosynthesis | 13 | 34 | 8.48E-05 |
| ko00909 | Sesquiterpenoid and triterpenoid biosynthesis | 10 | 22 | 0.000183282 |
| ko01200 | Carbon metabolism | 75 | 522 | 0.000238888 |
| ko00630 | Glyoxylate and dicarboxylate metabolism | 24 | 114 | 0.000434953 |
| ko00592 | alpha-Linolenic acid metabolism | 16 | 60 | 0.000446625 |
| ko04932 | Non-alcoholic fatty liver disease (NAFLD) | 31 | 171 | 0.000682236 |
| ko03050 | Proteasome | 19 | 88 | 0.001271232 |
| ko00030 | Pentose phosphate pathway | 17 | 80 | 0.002576176 |
| ko00010 | Glycolysis / Gluconeogenesis | 38 | 249 | 0.003044612 |
| ko00100 | Steroid biosynthesis | 12 | 50 | 0.004621148 |
| ko00905 | Brassinosteroid biosynthesis | 7 | 20 | 0.005506214 |
| ko00860 | Porphyrin and chlorophyll metabolism | 20 | 111 | 0.005942855 |
| ko01040 | Biosynthesis of unsaturated fatty acids | 13 | 59 | 0.006058749 |
| ko05012 | Parkinson's disease | 32 | 210 | 0.006257356 |
| ko00196 | Photosynthesis - antenna proteins | 10 | 39 | 0.00635714 |
| ko00040 | Pentose and glucuronate interconversions | 20 | 112 | 0.006469386 |
| ko00620 | Pyruvate metabolism | 25 | 153 | 0.006908242 |
| ko00903 | Limonene and pinene degradation | 5 | 11 | 0.007968508 |
| ko00750 | Vitamin B6 metabolism | 5 | 11 | 0.007968508 |
| ko00920 | Sulfur metabolism | 12 | 56 | 0.009819675 |
| ko04626 | Plant-pathogen interaction | 36 | 255 | 0.011060298 |
| ko00910 | Nitrogen metabolism | 11 | 50 | 0.011193566 |
| ko00980 | Metabolism of xenobiotics by cytochrome P450 | 13 | 66 | 0.013136797 |
| ko04740 | Olfactory transduction | 5 | 13 | 0.013593155 |
| ko00190 | Oxidative phosphorylation | 40 | 297 | 0.014848912 |
| ko01230 | Biosynthesis of amino acids | 64 | 522 | 0.015807519 |
| ko05204 | Chemical carcinogenesis | 12 | 61 | 0.016910453 |
| ko04744 | Phototransduction | 5 | 14 | 0.017208364 |
| ko00982 | Drug metabolism - cytochrome P450 | 13 | 70 | 0.019362324 |
| ko02020 | Two-component system | 8 | 35 | 0.02386249 |
| ko00521 | Streptomycin biosynthesis | 4 | 10 | 0.024327786 |
| ko00260 | Glycine, serine and threonine metabolism | 18 | 114 | 0.02576253 |
| ko00720 | Carbon fixation pathways in prokaryotes | 11 | 60 | 0.032158455 |
| ko04962 | Vasopressin-regulated water reabsorption | 10 | 54 | 0.037936379 |
| ko00230 | Purine metabolism | 36 | 284 | 0.04016657 |
| ko04978 | Mineral absorption | 6 | 26 | 0.045901414 |
| ko01212 | Fatty acid metabolism | 20 | 142 | 0.049078996 |
| ko00062 | Fatty acid elongation | 9 | 49 | 0.049292087 |
| ko03020 | RNA polymerase | 13 | 83 | 0.054858866 |
| ko00590 | Arachidonic acid metabolism | 5 | 21 | 0.060150571 |
| ko00071 | Fatty acid degradation | 13 | 86 | 0.067019961 |
| ko00053 | Ascorbate and aldarate metabolism | 13 | 86 | 0.067019961 |
| ko04971 | Gastric acid secretion | 5 | 22 | 0.068970163 |
| ko04722 | Neurotrophin signaling pathway | 29 | 234 | 0.073510543 |
| ko05416 | Viral myocarditis | 8 | 46 | 0.077000899 |
| ko00625 | Chloroalkane and chloroalkene degradation | 5 | 23 | 0.078462639 |
| ko00020 | Citrate cycle (TCA cycle) | 14 | 98 | 0.081715555 |
| ko04112 | Cell cycle - Caulobacter | 6 | 31 | 0.083073655 |
| ko00240 | Pyrimidine metabolism | 29 | 240 | 0.09130788 |
| ko04745 | Phototransduction - fly | 6 | 32 | 0.092050055 |
| ko04972 | Pancreatic secretion | 9 | 57 | 0.095575242 |
| ko00362 | Benzoate degradation | 4 | 18 | 0.105096244 |
| ko00350 | Tyrosine metabolism | 10 | 67 | 0.105276584 |
| ko04970 | Salivary secretion | 5 | 26 | 0.110838782 |
| ko03320 | PPAR signaling pathway | 9 | 60 | 0.117808558 |
| ko00430 | Taurine and hypotaurine metabolism | 4 | 19 | 0.119269011 |
| ko00960 | Tropane, piperidine and pyridine alkaloid biosynthesis | 7 | 44 | 0.128466709 |
| ko00680 | Methane metabolism | 17 | 137 | 0.141551266 |
| ko00950 | Isoquinoline alkaloid biosynthesis | 5 | 29 | 0.14861043 |
| ko05034 | Alcoholism | 25 | 216 | 0.14954237 |
| ko03030 | DNA replication | 14 | 112 | 0.163936377 |
| ko04961 | Endocrine and other factor-regulated calcium reabsorption | 6 | 39 | 0.168143051 |
| ko00900 | Terpenoid backbone biosynthesis | 14 | 114 | 0.178299839 |
| ko04670 | Leukocyte transendothelial migration | 6 | 40 | 0.180704671 |
| ko04391 | Hippo signaling pathway - fly | 8 | 58 | 0.181179115 |
| ko00520 | Amino sugar and nucleotide sugar metabolism | 28 | 253 | 0.184478203 |
| ko04146 | Peroxisome | 20 | 174 | 0.18848804 |
| ko00061 | Fatty acid biosynthesis | 9 | 68 | 0.1892072 |
| ko00250 | Alanine, aspartate and glutamate metabolism | 13 | 106 | 0.190453564 |
| ko04750 | Inflammatory mediator regulation of TRP channels | 6 | 41 | 0.193626026 |
| ko00253 | Tetracycline biosynthesis | 2 | 8 | 0.199853492 |
| ko05203 | Viral carcinogenesis | 30 | 278 | 0.208602522 |
| ko00051 | Fructose and mannose metabolism | 12 | 99 | 0.212397295 |
| ko04727 | GABAergic synapse | 9 | 71 | 0.219958923 |
| ko05169 | Epstein-Barr virus infection | 28 | 261 | 0.226461188 |
| ko01220 | Degradation of aromatic compounds | 3 | 17 | 0.229296221 |
| ko04145 | Phagosome | 22 | 202 | 0.238640014 |
| ko00650 | Butanoate metabolism | 5 | 36 | 0.253025136 |
| ko04064 | NF-kappa B signaling pathway | 10 | 85 | 0.265617089 |
| ko00730 | Thiamine metabolism | 4 | 28 | 0.274326894 |
| ko00450 | Selenocompound metabolism | 4 | 28 | 0.274326894 |
| ko05010 | Alzheimer's disease | 41 | 407 | 0.284609241 |
| ko05100 | Bacterial invasion of epithelial cells | 8 | 67 | 0.284916744 |
| ko04014 | Ras signaling pathway | 19 | 179 | 0.292151246 |
| ko04260 | Cardiac muscle contraction | 7 | 58 | 0.295539612 |
| ko04973 | Carbohydrate digestion and absorption | 3 | 20 | 0.298878955 |
| ko00340 | Histidine metabolism | 6 | 49 | 0.30691937 |
| ko00400 | Phenylalanine, tyrosine and tryptophan biosynthesis | 16 | 151 | 0.315516576 |
| ko04110 | Cell cycle | 22 | 214 | 0.320815729 |
| ko04066 | HIF-1 signaling pathway | 19 | 183 | 0.322886424 |
| ko04623 | Cytosolic DNA-sensing pathway | 5 | 42 | 0.352969748 |
| ko00561 | Glycerolipid metabolism | 14 | 135 | 0.358242578 |
| ko03060 | Protein export | 11 | 104 | 0.36088679 |
| ko04015 | Rap1 signaling pathway | 17 | 167 | 0.363013852 |
| ko04612 | Antigen processing and presentation | 10 | 94 | 0.365111026 |
| ko04721 | Synaptic vesicle cycle | 10 | 94 | 0.365111026 |
| ko05133 | Pertussis | 18 | 178 | 0.366888634 |
| ko04918 | Thyroid hormone synthesis | 5 | 43 | 0.369956581 |
| ko00591 | Linoleic acid metabolism | 5 | 43 | 0.369956581 |
| ko00565 | Ether lipid metabolism | 9 | 85 | 0.381539412 |
| ko04910 | Insulin signaling pathway | 25 | 256 | 0.39384608 |
| ko00380 | Tryptophan metabolism | 8 | 76 | 0.399745637 |
| ko00524 | Butirosin and neomycin biosynthesis | 1 | 5 | 0.406301987 |
| ko04620 | Toll-like receptor signaling pathway | 18 | 183 | 0.408495653 |
| ko00130 | Ubiquinone and other terpenoid-quinone biosynthesis | 8 | 79 | 0.43858808 |
| ko00908 | Zeatin biosynthesis | 9 | 90 | 0.44221547 |
| ko05120 | Epithelial cell signaling in Helicobacter pylori infection | 6 | 58 | 0.44354868 |
| ko00514 | Other types of O-glycan biosynthesis | 1 | 6 | 0.455719006 |
| ko04111 | Cell cycle - yeast | 16 | 168 | 0.465368176 |
| ko04920 | Adipocytokine signaling pathway | 6 | 61 | 0.488378415 |
| ko00906 | Carotenoid biosynthesis | 8 | 83 | 0.489783601 |
| ko00401 | Novobiocin biosynthesis | 1 | 7 | 0.501023856 |
| ko05130 | Pathogenic Escherichia coli infection | 9 | 95 | 0.502117044 |
| ko00760 | Nicotinate and nicotinamide metabolism | 3 | 29 | 0.50449316 |
| ko04011 | MAPK signaling pathway - yeast | 2 | 18 | 0.504786284 |
| ko04024 | cAMP signaling pathway | 20 | 218 | 0.515895261 |
| ko04976 | Bile secretion | 7 | 74 | 0.516121648 |
| ko05206 | MicroRNAs in cancer | 18 | 199 | 0.541495486 |
| ko04666 | Fc gamma R-mediated phagocytosis | 18 | 201 | 0.557618614 |
| ko05231 | Choline metabolism in cancer | 17 | 191 | 0.567589364 |
| ko05134 | Legionellosis | 16 | 181 | 0.578128471 |
| ko04144 | Endocytosis | 19 | 217 | 0.594434152 |
| ko05164 | Influenza A | 22 | 254 | 0.61562147 |
| ko04390 | Hippo signaling pathway | 8 | 94 | 0.62163639 |
| ko03070 | Bacterial secretion system | 4 | 47 | 0.622822801 |
| ko05222 | Small cell lung cancer | 3 | 35 | 0.623034905 |
| ko04070 | Phosphatidylinositol signaling system | 11 | 129 | 0.623498797 |
| ko00780 | Biotin metabolism | 3 | 36 | 0.640791745 |
| ko05211 | Renal cell carcinoma | 11 | 131 | 0.642310026 |
| ko00140 | Steroid hormone biosynthesis | 1 | 11 | 0.647550715 |
| ko04912 | GnRH signaling pathway | 13 | 155 | 0.648692903 |
| ko04915 | Estrogen signaling pathway | 15 | 179 | 0.65491972 |
| ko04330 | Notch signaling pathway | 2 | 25 | 0.669680466 |
| ko04919 | Thyroid hormone signaling pathway | 16 | 196 | 0.694697182 |
| ko00052 | Galactose metabolism | 10 | 126 | 0.703743699 |
| ko04630 | Jak-STAT signaling pathway | 1 | 13 | 0.703790284 |
| ko05162 | Measles | 12 | 151 | 0.712399157 |
| ko03013 | RNA transport | 25 | 305 | 0.717361178 |
| ko05145 | Toxoplasmosis | 17 | 212 | 0.723326068 |
| ko00790 | Folate biosynthesis | 4 | 55 | 0.73438268 |
| ko00330 | Arginine and proline metabolism | 14 | 178 | 0.734962428 |
| ko05132 | Salmonella infection | 12 | 154 | 0.735318163 |
| ko01210 | 2-Oxocarboxylic acid metabolism | 7 | 93 | 0.736036614 |
| ko04664 | Fc epsilon RI signaling pathway | 9 | 118 | 0.739024152 |
| ko05212 | Pancreatic cancer | 10 | 131 | 0.745171686 |
| ko00072 | Synthesis and degradation of ketone bodies | 1 | 15 | 0.75105803 |
| ko00626 | Naphthalene degradation | 1 | 15 | 0.75105803 |
| ko05217 | Basal cell carcinoma | 1 | 15 | 0.75105803 |
| ko05214 | Glioma | 10 | 132 | 0.752964236 |
| ko04151 | PI3K-Akt signaling pathway | 22 | 277 | 0.757019388 |
| ko05142 | Chagas disease (American trypanosomiasis) | 14 | 182 | 0.76176781 |
| ko02010 | ABC transporters | 8 | 109 | 0.767092406 |
| ko03440 | Homologous recombination | 7 | 97 | 0.772040032 |
| ko04930 | Type II diabetes mellitus | 8 | 110 | 0.775158646 |
| ko05230 | Central carbon metabolism in cancer | 15 | 197 | 0.77950458 |
| ko00460 | Cyanoamino acid metabolism | 7 | 98 | 0.780451643 |
| ko04130 | SNARE interactions in vesicular transport | 8 | 111 | 0.783019549 |
| ko04810 | Regulation of actin cytoskeleton | 18 | 234 | 0.783167738 |
| ko04152 | AMPK signaling pathway | 12 | 161 | 0.783998349 |
| ko00670 | One carbon pool by folate | 4 | 60 | 0.790333998 |
| ko05033 | Nicotine addiction | 1 | 17 | 0.790784862 |
| ko04916 | Melanogenesis | 10 | 138 | 0.796250598 |
| ko05110 | Vibrio cholerae infection | 7 | 100 | 0.79657759 |
| ko00627 | Aminobenzoate degradation | 1 | 18 | 0.808204173 |
| ko00440 | Phosphonate and phosphinate metabolism | 1 | 18 | 0.808204173 |
| ko05014 | Amyotrophic lateral sclerosis (ALS) | 11 | 153 | 0.811903195 |
| ko04728 | Dopaminergic synapse | 6 | 89 | 0.812222571 |
| ko05146 | Amoebiasis | 2 | 34 | 0.813184816 |
| ko04062 | Chemokine signaling pathway | 9 | 129 | 0.820301741 |
| ko00310 | Lysine degradation | 5 | 77 | 0.822576903 |
| ko04975 | Fat digestion and absorption | 1 | 19 | 0.824173535 |
| ko00983 | Drug metabolism - other enzymes | 2 | 36 | 0.836324852 |
| ko04710 | Circadian rhythm | 2 | 36 | 0.836324852 |
| ko00830 | Retinol metabolism | 1 | 20 | 0.838813608 |
| ko00564 | Glycerophospholipid metabolism | 16 | 221 | 0.840738346 |
| ko05200 | Pathways in cancer | 20 | 271 | 0.842902533 |
| ko04713 | Circadian entrainment | 7 | 107 | 0.845952153 |
| ko05410 | Hypertrophic cardiomyopathy (HCM) | 3 | 52 | 0.846973325 |
| ko04071 | Sphingolipid signaling pathway | 16 | 223 | 0.849671829 |
| ko05032 | Morphine addiction | 1 | 21 | 0.852235012 |
| ko00562 | Inositol phosphate metabolism | 12 | 174 | 0.856777348 |
| ko03008 | Ribosome biogenesis in eukaryotes | 12 | 175 | 0.861471246 |
| ko00740 | Riboflavin metabolism | 1 | 22 | 0.864539165 |
| ko04340 | Hedgehog signaling pathway | 1 | 22 | 0.864539165 |
| ko04270 | Vascular smooth muscle contraction | 10 | 150 | 0.865595209 |
| ko05140 | Leishmaniasis | 11 | 163 | 0.865612825 |
| ko04068 | FoxO signaling pathway | 15 | 216 | 0.872971108 |
| ko04914 | Progesterone-mediated oocyte maturation | 12 | 178 | 0.87482699 |
| ko04974 | Protein digestion and absorption | 2 | 40 | 0.874974862 |
| ko04940 | Type I diabetes mellitus | 6 | 99 | 0.878644612 |
| ko04540 | Gap junction | 10 | 153 | 0.879601241 |
| ko00770 | Pantothenate and CoA biosynthesis | 2 | 41 | 0.883225819 |
| ko03018 | RNA degradation | 25 | 345 | 0.885464443 |
| ko00511 | Other glycan degradation | 3 | 57 | 0.885705955 |
| ko04122 | Sulfur relay system | 2 | 42 | 0.890969877 |
| ko04520 | Adherens junction | 9 | 145 | 0.902002353 |
| ko05131 | Shigellosis | 11 | 174 | 0.909772967 |
| ko05205 | Proteoglycans in cancer | 15 | 227 | 0.910770596 |
| ko05210 | Colorectal cancer | 11 | 175 | 0.91310412 |
| ko04141 | Protein processing in endoplasmic reticulum | 32 | 447 | 0.921636198 |
| ko00600 | Sphingolipid metabolism | 4 | 79 | 0.922520688 |
| ko05412 | Arrhythmogenic right ventricular cardiomyopathy (ARVC) | 1 | 29 | 0.926297486 |
| ko04142 | Lysosome | 11 | 180 | 0.928244304 |
| ko05161 | Hepatitis B | 9 | 153 | 0.929439075 |
| ko05202 | Transcriptional misregulation in cancer | 4 | 81 | 0.930745265 |
| ko05221 | Acute myeloid leukemia | 6 | 112 | 0.934435247 |
| ko05414 | Dilated cardiomyopathy | 1 | 31 | 0.93806297 |
| ko00410 | beta-Alanine metabolism | 8 | 142 | 0.938088407 |
| ko05323 | Rheumatoid arthritis | 2 | 51 | 0.942003582 |
| ko00603 | Glycosphingolipid biosynthesis - globo series | 1 | 32 | 0.943221623 |
| ko04210 | Apoptosis | 11 | 187 | 0.945610536 |
| ko00970 | Aminoacyl-tRNA biosynthesis | 11 | 188 | 0.947765188 |
| ko05215 | Prostate cancer | 9 | 161 | 0.949963265 |
| ko00531 | Glycosaminoglycan degradation | 1 | 36 | 0.95990375 |
| ko04012 | ErbB signaling pathway | 6 | 122 | 0.960451315 |
| ko04621 | NOD-like receptor signaling pathway | 5 | 107 | 0.961326473 |
| ko05020 | Prion diseases | 6 | 127 | 0.969565382 |
| ko04150 | mTOR signaling pathway | 7 | 143 | 0.970618517 |
| ko04668 | TNF signaling pathway | 5 | 112 | 0.970732171 |
| ko00500 | Starch and sucrose metabolism | 33 | 497 | 0.97105505 |
| ko04140 | Regulation of autophagy | 3 | 79 | 0.971296032 |
| ko03450 | Non-homologous end-joining | 1 | 40 | 0.971685483 |
| ko05152 | Tuberculosis | 29 | 459 | 0.981050876 |
| ko04966 | Collecting duct acid secretion | 1 | 45 | 0.981671824 |
| ko04013 | MAPK signaling pathway - fly | 4 | 105 | 0.983427103 |
| ko04020 | Calcium signaling pathway | 8 | 170 | 0.983749879 |
| ko00640 | Propanoate metabolism | 6 | 140 | 0.984986663 |
| ko04320 | Dorso-ventral axis formation | 4 | 107 | 0.985375839 |
| ko04725 | Cholinergic synapse | 4 | 109 | 0.987105501 |
| ko04114 | Oocyte meiosis | 21 | 363 | 0.987316325 |
| ko04712 | Circadian rhythm - plant | 5 | 127 | 0.987726627 |
| ko05216 | Thyroid cancer | 4 | 110 | 0.987895487 |
| ko04261 | Adrenergic signaling in cardiomyocytes | 8 | 177 | 0.988647639 |
| ko04510 | Focal adhesion | 11 | 227 | 0.990661899 |
| ko04726 | Serotonergic synapse | 4 | 115 | 0.99120025 |
| ko04917 | Prolactin signaling pathway | 4 | 115 | 0.99120025 |
| ko05218 | Melanoma | 4 | 116 | 0.991748274 |
| ko03022 | Basal transcription factors | 4 | 116 | 0.991748274 |
| ko05223 | Non-small cell lung cancer | 4 | 116 | 0.991748274 |
| ko05219 | Bladder cancer | 4 | 116 | 0.991748274 |
| ko05168 | Herpes simplex infection | 9 | 200 | 0.992221732 |
| ko04550 | Signaling pathways regulating pluripotency of stem cells | 4 | 117 | 0.99226349 |
| ko04730 | Long-term depression | 4 | 117 | 0.99226349 |
| ko05220 | Chronic myeloid leukemia | 4 | 118 | 0.992747758 |
| ko03460 | Fanconi anemia pathway | 6 | 153 | 0.992832358 |
| ko05031 | Amphetamine addiction | 6 | 154 | 0.993236907 |
| ko03430 | Mismatch repair | 4 | 122 | 0.994409247 |
| ko05213 | Endometrial cancer | 4 | 125 | 0.995408022 |
| ko04723 | Retrograde endocannabinoid signaling | 3 | 107 | 0.995770237 |
| ko04380 | Osteoclast differentiation | 10 | 228 | 0.99585335 |
| ko00280 | Valine, leucine and isoleucine degradation | 8 | 196 | 0.995885428 |
| ko04350 | TGF-beta signaling pathway | 4 | 128 | 0.996233479 |
| ko05166 | HTLV-I infection | 13 | 281 | 0.996968924 |
| ko04115 | p53 signaling pathway | 5 | 153 | 0.99752912 |
| ko04650 | Natural killer cell mediated cytotoxicity | 9 | 228 | 0.998247962 |
| ko04960 | Aldosterone-regulated sodium reabsorption | 2 | 98 | 0.998317428 |
| ko04360 | Axon guidance | 9 | 230 | 0.998431328 |
| ko04010 | MAPK signaling pathway | 11 | 264 | 0.998534262 |
| ko04720 | Long-term potentiation | 11 | 264 | 0.998534262 |
| ko04724 | Glutamatergic synapse | 10 | 248 | 0.998553991 |
| ko04662 | B cell receptor signaling pathway | 9 | 235 | 0.998812708 |
| ko04611 | Platelet activation | 4 | 146 | 0.998882036 |
| ko04370 | VEGF signaling pathway | 9 | 237 | 0.998938799 |
| ko04120 | Ubiquitin mediated proteolysis | 12 | 289 | 0.999078408 |
| ko04310 | Wnt signaling pathway | 6 | 188 | 0.999140878 |
| ko03420 | Nucleotide excision repair | 5 | 171 | 0.999230315 |
| ko05160 | Hepatitis C | 3 | 131 | 0.999250231 |
| ko04530 | Tight junction | 1 | 83 | 0.999328927 |
| ko03410 | Base excision repair | 3 | 133 | 0.999352701 |
| ko03015 | mRNA surveillance pathway | 7 | 215 | 0.999502848 |
| ko04113 | Meiosis - yeast | 12 | 301 | 0.999510919 |
| ko04921 | Oxytocin signaling pathway | 13 | 321 | 0.999597053 |
| ko04022 | cGMP-PKG signaling pathway | 11 | 293 | 0.999694616 |
| ko00513 | Various types of N-glycan biosynthesis | 1 | 118 | 0.999968188 |
| ko00510 | N-Glycan biosynthesis | 2 | 159 | 0.999987198 |
| ko03040 | Spliceosome | 18 | 496 | 0.999997135 |
| ko04660 | T cell receptor signaling pathway | 4 | 230 | 0.99999742 |
