# Supplementary material for: De novo Sequencing and Transcriptome Analysis Reveal Key Genes Regulating Steroid Metabolism in Leaves, Roots, Adventitious Roots and Calli of Periploca sepium Bunge
Source: Front Plant Sci. 2017 Apr 21;8:594. doi: 10.3389/fpls.2017.00594 (PMC5399629; doi:10.3389/fpls.2017.00594)
Supplement: Supplementary file 12 [file Table12.DOC]

**Table S12. Statistical analysis for the KEGG enrichment of R vs L ( *p* ≤ 0.05).**

| **ID** | **Term** | **Input number** | **Background number** | **P-Value** |
| --- | --- | --- | --- | --- |
| ko00195 | Photosynthesis | 35 | 88 | 3.80E-20 |
| ko00940 | Phenylpropanoid biosynthesis | 47 | 236 | 3.22E-16 |
| ko00360 | Phenylalanine metabolism | 31 | 149 | 1.31E-11 |
| ko04075 | Plant hormone signal transduction | 41 | 307 | 1.67E-09 |
| ko00710 | Carbon fixation in photosynthetic organisms | 25 | 127 | 3.28E-09 |
| ko00908 | Zeatin biosynthesis | 17 | 90 | 1.70E-06 |
| ko00030 | Pentose phosphate pathway | 16 | 80 | 1.78E-06 |
| ko00945 | Stilbenoid, diarylheptanoid and gingerol biosynthesis | 8 | 22 | 2.05E-05 |
| ko01200 | Carbon metabolism | 45 | 522 | 2.28E-05 |
| ko00196 | Photosynthesis - antenna proteins | 10 | 39 | 2.50E-05 |
| ko00905 | Brassinosteroid biosynthesis | 7 | 20 | 8.31E-05 |
| ko00630 | Glyoxylate and dicarboxylate metabolism | 16 | 114 | 8.84E-05 |
| ko00040 | Pentose and glucuronate interconversions | 15 | 112 | 0.000229942 |
| ko00500 | Starch and sucrose metabolism | 40 | 497 | 0.000239689 |
| ko00941 | Flavonoid biosynthesis | 8 | 34 | 0.00026747 |
| ko00860 | Porphyrin and chlorophyll metabolism | 14 | 111 | 0.000630912 |
| ko00480 | Glutathione metabolism | 14 | 117 | 0.001005019 |
| ko00592 | alpha-Linolenic acid metabolism | 9 | 60 | 0.001986557 |
| ko05204 | Chemical carcinogenesis | 9 | 61 | 0.002198139 |
| ko00980 | Metabolism of xenobiotics by cytochrome P450 | 9 | 66 | 0.003539583 |
| ko00350 | Tyrosine metabolism | 9 | 67 | 0.003871948 |
| ko00909 | Sesquiterpenoid and triterpenoid biosynthesis | 5 | 22 | 0.004382859 |
| ko00520 | Amino sugar and nucleotide sugar metabolism | 21 | 253 | 0.004774237 |
| ko00982 | Drug metabolism - cytochrome P450 | 9 | 70 | 0.005017132 |
| ko00260 | Glycine, serine and threonine metabolism | 12 | 114 | 0.00574594 |
| ko00010 | Glycolysis / Gluconeogenesis | 20 | 249 | 0.0080014 |
| ko04978 | Mineral absorption | 5 | 26 | 0.008058382 |
| ko04626 | Plant-pathogen interaction | 20 | 255 | 0.010059858 |
| ko05130 | Pathogenic Escherichia coli infection | 10 | 95 | 0.011138622 |
| ko00073 | Cutin, suberine and wax biosynthesis | 4 | 18 | 0.011420112 |
| ko00051 | Fructose and mannose metabolism | 10 | 99 | 0.014237339 |
| ko00960 | Tropane, piperidine and pyridine alkaloid biosynthesis | 6 | 44 | 0.016062846 |
| ko04745 | Phototransduction - fly | 5 | 32 | 0.016900876 |
| ko04391 | Hippo signaling pathway - fly | 7 | 58 | 0.016925051 |
| ko00903 | Limonene and pinene degradation | 3 | 11 | 0.017860995 |
| ko01040 | Biosynthesis of unsaturated fatty acids | 7 | 59 | 0.018283388 |
| ko03320 | PPAR signaling pathway | 7 | 60 | 0.019716433 |
| ko00062 | Fatty acid elongation | 6 | 49 | 0.024694653 |
| ko00270 | Cysteine and methionine metabolism | 12 | 142 | 0.02519788 |
| ko04740 | Olfactory transduction | 3 | 13 | 0.025857497 |
| ko00130 | Ubiquinone and other terpenoid-quinone biosynthesis | 8 | 79 | 0.026419373 |
| ko04744 | Phototransduction | 3 | 14 | 0.030461492 |
| ko03010 | Ribosome | 30 | 478 | 0.030568835 |
| ko01230 | Biosynthesis of amino acids | 32 | 522 | 0.034082758 |
| ko00290 | Valine, leucine and isoleucine biosynthesis | 3 | 15 | 0.035465624 |
| ko00053 | Ascorbate and aldarate metabolism | 8 | 86 | 0.039349969 |
| ko00620 | Pyruvate metabolism | 12 | 153 | 0.039786333 |
| ko00920 | Sulfur metabolism | 6 | 56 | 0.041274619 |
| ko00680 | Methane metabolism | 11 | 137 | 0.041789599 |
| ko00950 | Isoquinoline alkaloid biosynthesis | 4 | 29 | 0.04487267 |
| ko00052 | Galactose metabolism | 10 | 126 | 0.053766347 |
| ko04145 | Phagosome | 14 | 202 | 0.062425695 |
| ko00460 | Cyanoamino acid metabolism | 8 | 98 | 0.070150302 |
| ko00590 | Arachidonic acid metabolism | 3 | 21 | 0.073493617 |
| ko00910 | Nitrogen metabolism | 5 | 50 | 0.074406981 |
| ko05322 | Systemic lupus erythematosus | 5 | 50 | 0.074406981 |
| ko04971 | Gastric acid secretion | 3 | 22 | 0.081071364 |
| ko00061 | Fatty acid biosynthesis | 6 | 68 | 0.083075311 |
| ko00521 | Streptomycin biosynthesis | 2 | 10 | 0.084515595 |
| ko00625 | Chloroalkane and chloroalkene degradation | 3 | 23 | 0.088970595 |
| ko01212 | Fatty acid metabolism | 10 | 142 | 0.096170552 |
| ko04976 | Bile secretion | 6 | 74 | 0.110378192 |
| ko00770 | Pantothenate and CoA biosynthesis | 4 | 41 | 0.112018952 |
| ko04970 | Salivary secretion | 3 | 26 | 0.114456899 |
| ko00380 | Tryptophan metabolism | 6 | 76 | 0.120382957 |
| ko04390 | Hippo signaling pathway | 7 | 94 | 0.121452091 |
| ko04918 | Thyroid hormone synthesis | 4 | 43 | 0.126030331 |
| ko04722 | Neurotrophin signaling pathway | 14 | 234 | 0.143199334 |
| ko03020 | RNA polymerase | 6 | 83 | 0.158693169 |
| ko00906 | Carotenoid biosynthesis | 6 | 83 | 0.158693169 |
| ko04112 | Cell cycle - Caulobacter | 3 | 31 | 0.162010375 |
| ko04152 | AMPK signaling pathway | 10 | 161 | 0.166232345 |
| ko00071 | Fatty acid degradation | 6 | 86 | 0.176552189 |
| ko01220 | Degradation of aromatic compounds | 2 | 17 | 0.182052155 |
| ko00250 | Alanine, aspartate and glutamate metabolism | 7 | 106 | 0.182505882 |
| ko00627 | Aminobenzoate degradation | 2 | 18 | 0.197096647 |
| ko00650 | Butanoate metabolism | 3 | 36 | 0.214286369 |
| ko00780 | Biotin metabolism | 3 | 36 | 0.214286369 |
| ko04540 | Gap junction | 9 | 153 | 0.222067002 |
| ko04080 | Neuroactive ligand-receptor interaction | 1 | 5 | 0.222229349 |
| ko04146 | Peroxisome | 10 | 174 | 0.225091479 |
| ko00230 | Purine metabolism | 15 | 284 | 0.248329828 |
| ko00660 | C5-Branched dibasic acid metabolism | 1 | 6 | 0.25413799 |
| ko00720 | Carbon fixation pathways in prokaryotes | 4 | 60 | 0.267658252 |
| ko04750 | Inflammatory mediator regulation of TRP channels | 3 | 41 | 0.269493902 |
| ko00643 | Styrene degradation | 1 | 7 | 0.284738334 |
| ko00401 | Novobiocin biosynthesis | 1 | 7 | 0.284738334 |
| ko00591 | Linoleic acid metabolism | 3 | 43 | 0.292030986 |
| ko00190 | Oxidative phosphorylation | 15 | 297 | 0.301353146 |
| ko05164 | Influenza A | 13 | 254 | 0.303241023 |
| ko04064 | NF-kappa B signaling pathway | 5 | 85 | 0.309821306 |
| ko04910 | Insulin signaling pathway | 13 | 256 | 0.312409752 |
| ko00253 | Tetracycline biosynthesis | 1 | 8 | 0.31408399 |
| ko04070 | Phosphatidylinositol signaling system | 7 | 129 | 0.32479897 |
| ko00400 | Phenylalanine, tyrosine and tryptophan biosynthesis | 8 | 151 | 0.328815158 |
| ko05162 | Measles | 8 | 151 | 0.328815158 |
| ko03070 | Bacterial secretion system | 3 | 47 | 0.33737308 |
| ko00240 | Pyrimidine metabolism | 12 | 240 | 0.340186602 |
| ko00450 | Selenocompound metabolism | 2 | 28 | 0.350134309 |
| ko05133 | Pertussis | 9 | 178 | 0.360472627 |
| ko00760 | Nicotinate and nicotinamide metabolism | 2 | 29 | 0.365123438 |
| ko05412 | Arrhythmogenic right ventricular cardiomyopathy (ARVC) | 2 | 29 | 0.365123438 |
| ko00100 | Steroid biosynthesis | 3 | 50 | 0.371293599 |
| ko01210 | 2-Oxocarboxylic acid metabolism | 5 | 93 | 0.37510157 |
| ko04612 | Antigen processing and presentation | 5 | 94 | 0.383293712 |
| ko05410 | Hypertrophic cardiomyopathy (HCM) | 3 | 52 | 0.393730761 |
| ko05414 | Dilated cardiomyopathy | 2 | 31 | 0.394681338 |
| ko00750 | Vitamin B6 metabolism | 1 | 11 | 0.395096539 |
| ko04015 | Rap1 signaling pathway | 8 | 167 | 0.428436852 |
| ko05146 | Amoebiasis | 2 | 34 | 0.437775872 |
| ko05034 | Alcoholism | 10 | 216 | 0.448992826 |
| ko00562 | Inositol phosphate metabolism | 8 | 174 | 0.471882816 |
| ko00670 | One carbon pool by folate | 3 | 60 | 0.480796331 |
| ko05206 | MicroRNAs in cancer | 9 | 199 | 0.483007223 |
| ko00626 | Naphthalene degradation | 1 | 15 | 0.488436422 |
| ko05214 | Glioma | 6 | 132 | 0.500924828 |
| ko04915 | Estrogen signaling pathway | 8 | 179 | 0.502454752 |
| ko02010 | ABC transporters | 5 | 109 | 0.503790226 |
| ko04974 | Protein digestion and absorption | 2 | 40 | 0.518580719 |
| ko04670 | Leukocyte transendothelial migration | 2 | 40 | 0.518580719 |
| ko04130 | SNARE interactions in vesicular transport | 5 | 111 | 0.519229558 |
| ko00561 | Glycerolipid metabolism | 6 | 135 | 0.521945381 |
| ko00440 | Phosphonate and phosphinate metabolism | 1 | 18 | 0.548866782 |
| ko04011 | MAPK signaling pathway - yeast | 1 | 18 | 0.548866782 |
| ko05145 | Toxoplasmosis | 9 | 212 | 0.556233124 |
| ko05169 | Epstein-Barr virus infection | 11 | 261 | 0.562075265 |
| ko04975 | Fat digestion and absorption | 1 | 19 | 0.567381 |
| ko04144 | Endocytosis | 9 | 217 | 0.583266745 |
| ko00300 | Lysine biosynthesis | 1 | 20 | 0.585135858 |
| ko00830 | Retinol metabolism | 1 | 20 | 0.585135858 |
| ko00904 | Diterpenoid biosynthesis | 1 | 20 | 0.585135858 |
| ko04973 | Carbohydrate digestion and absorption | 1 | 20 | 0.585135858 |
| ko05416 | Viral myocarditis | 2 | 46 | 0.591258357 |
| ko00020 | Citrate cycle (TCA cycle) | 4 | 98 | 0.606527593 |
| ko00340 | Histidine metabolism | 2 | 49 | 0.624405282 |
| ko03060 | Protein export | 4 | 104 | 0.651287383 |
| ko05142 | Chagas disease (American trypanosomiasis) | 7 | 182 | 0.660807649 |
| ko04620 | Toll-like receptor signaling pathway | 7 | 183 | 0.666173195 |
| ko04066 | HIF-1 signaling pathway | 7 | 183 | 0.666173195 |
| ko04713 | Circadian entrainment | 4 | 107 | 0.672349352 |
| ko04530 | Tight junction | 3 | 83 | 0.690388025 |
| ko05140 | Leishmaniasis | 6 | 163 | 0.696374909 |
| ko04916 | Melanogenesis | 5 | 138 | 0.702516778 |
| ko00730 | Thiamine metabolism | 1 | 28 | 0.703317912 |
| ko00900 | Terpenoid backbone biosynthesis | 4 | 114 | 0.718001956 |
| ko00410 | beta-Alanine metabolism | 5 | 142 | 0.725054639 |
| ko04728 | Dopaminergic synapse | 3 | 89 | 0.733243145 |
| ko04920 | Adipocytokine signaling pathway | 2 | 61 | 0.736245411 |
| ko00603 | Glycosphingolipid biosynthesis - globo series | 1 | 32 | 0.749116169 |
| ko04270 | Vascular smooth muscle contraction | 5 | 150 | 0.766376963 |
| ko00330 | Arginine and proline metabolism | 6 | 178 | 0.770100723 |
| ko04014 | Ras signaling pathway | 6 | 179 | 0.77450085 |
| ko02020 | Two-component system | 1 | 35 | 0.778765064 |
| ko05222 | Small cell lung cancer | 1 | 35 | 0.778765064 |
| ko05100 | Bacterial invasion of epithelial cells | 2 | 67 | 0.780667986 |
| ko05132 | Salmonella infection | 5 | 154 | 0.78519093 |
| ko00983 | Drug metabolism - other enzymes | 1 | 36 | 0.787848338 |
| ko04710 | Circadian rhythm | 1 | 36 | 0.787848338 |
| ko04912 | GnRH signaling pathway | 5 | 155 | 0.789706094 |
| ko05110 | Vibrio cholerae infection | 3 | 100 | 0.799475864 |
| ko04727 | GABAergic synapse | 2 | 71 | 0.806509352 |
| ko04068 | FoxO signaling pathway | 7 | 216 | 0.812774506 |
| ko04151 | PI3K-Akt signaling pathway | 9 | 277 | 0.833115704 |
| ko04623 | Cytosolic DNA-sensing pathway | 1 | 42 | 0.835034598 |
| ko05203 | Viral carcinogenesis | 9 | 278 | 0.836017644 |
| ko04919 | Thyroid hormone signaling pathway | 6 | 196 | 0.839874843 |
| ko00280 | Valine, leucine and isoleucine degradation | 6 | 196 | 0.839874843 |
| ko00310 | Lysine degradation | 2 | 77 | 0.840192255 |
| ko05230 | Central carbon metabolism in cancer | 6 | 197 | 0.843189521 |
| ko04140 | Regulation of autophagy | 2 | 79 | 0.850181251 |
| ko04668 | TNF signaling pathway | 3 | 112 | 0.855421823 |
| ko04810 | Regulation of actin cytoskeleton | 7 | 234 | 0.868587895 |
| ko05218 | Melanoma | 3 | 116 | 0.870775119 |
| ko04921 | Oxytocin signaling pathway | 10 | 321 | 0.875297833 |
| ko05152 | Tuberculosis | 15 | 459 | 0.878340113 |
| ko05134 | Legionellosis | 5 | 181 | 0.88284846 |
| ko04115 | p53 signaling pathway | 4 | 153 | 0.889498457 |
| ko05161 | Hepatitis B | 4 | 153 | 0.889498457 |
| ko04210 | Apoptosis | 5 | 187 | 0.898477448 |
| ko05213 | Endometrial cancer | 3 | 125 | 0.900150847 |
| ko04962 | Vasopressin-regulated water reabsorption | 1 | 54 | 0.900268 |
| ko00790 | Folate biosynthesis | 1 | 55 | 0.904364694 |
| ko00564 | Glycerophospholipid metabolism | 6 | 221 | 0.907296573 |
| ko05231 | Choline metabolism in cancer | 5 | 191 | 0.907863001 |
| ko04721 | Synaptic vesicle cycle | 2 | 94 | 0.908679678 |
| ko05215 | Prostate cancer | 4 | 161 | 0.910404055 |
| ko00511 | Other glycan degradation | 1 | 57 | 0.912060443 |
| ko04260 | Cardiac muscle contraction | 1 | 58 | 0.915673015 |
| ko03018 | RNA degradation | 10 | 345 | 0.9200139 |
| ko04940 | Type I diabetes mellitus | 2 | 99 | 0.922863717 |
| ko04020 | Calcium signaling pathway | 4 | 170 | 0.929651922 |
| ko05131 | Shigellosis | 4 | 174 | 0.936940544 |
| ko04013 | MAPK signaling pathway - fly | 2 | 105 | 0.937143468 |
| ko04621 | NOD-like receptor signaling pathway | 2 | 107 | 0.941318417 |
| ko04320 | Dorso-ventral axis formation | 2 | 107 | 0.941318417 |
| ko04150 | mTOR signaling pathway | 3 | 143 | 0.941533716 |
| ko04261 | Adrenergic signaling in cardiomyocytes | 4 | 177 | 0.941949182 |
| ko04520 | Adherens junction | 3 | 145 | 0.944987799 |
| ko04725 | Cholinergic synapse | 2 | 109 | 0.945228807 |
| ko04611 | Platelet activation | 3 | 146 | 0.946642824 |
| ko05216 | Thyroid cancer | 2 | 110 | 0.947089675 |
| ko05221 | Acute myeloid leukemia | 2 | 112 | 0.950632091 |
| ko04024 | cAMP signaling pathway | 5 | 218 | 0.953520503 |
| ko04726 | Serotonergic synapse | 2 | 115 | 0.955523984 |
| ko04917 | Prolactin signaling pathway | 2 | 115 | 0.955523984 |
| ko05014 | Amyotrophic lateral sclerosis (ALS) | 3 | 153 | 0.956991342 |
| ko05223 | Non-small cell lung cancer | 2 | 116 | 0.957048915 |
| ko05219 | Bladder cancer | 2 | 116 | 0.957048915 |
| ko05031 | Amphetamine addiction | 3 | 154 | 0.958306138 |
| ko04730 | Long-term depression | 2 | 117 | 0.95852368 |
| ko04550 | Signaling pathways regulating pluripotency of stem cells | 2 | 117 | 0.95852368 |
| ko05220 | Chronic myeloid leukemia | 2 | 118 | 0.959949828 |
| ko04664 | Fc epsilon RI signaling pathway | 2 | 118 | 0.959949828 |
| ko04510 | Focal adhesion | 5 | 227 | 0.963380757 |
| ko05205 | Proteoglycans in cancer | 5 | 227 | 0.963380757 |
| ko00600 | Sphingolipid metabolism | 1 | 79 | 0.965063031 |
| ko04012 | ErbB signaling pathway | 2 | 122 | 0.965197696 |
| ko05202 | Transcriptional misregulation in cancer | 1 | 81 | 0.967876086 |
| ko04114 | Oocyte meiosis | 9 | 363 | 0.970406219 |
| ko04666 | Fc gamma R-mediated phagocytosis | 4 | 201 | 0.970666756 |
| ko04712 | Circadian rhythm - plant | 2 | 127 | 0.970832189 |
| ko05020 | Prion diseases | 2 | 127 | 0.970832189 |
| ko04062 | Chemokine signaling pathway | 2 | 129 | 0.972830218 |
| ko00565 | Ether lipid metabolism | 1 | 85 | 0.972841284 |
| ko05211 | Renal cell carcinoma | 2 | 131 | 0.974695737 |
| ko05212 | Pancreatic cancer | 2 | 131 | 0.974695737 |
| ko03050 | Proteasome | 1 | 88 | 0.976055001 |
| ko03008 | Ribosome biogenesis in eukaryotes | 3 | 175 | 0.978556108 |
| ko04110 | Cell cycle | 4 | 214 | 0.980008128 |
| ko04142 | Lysosome | 3 | 180 | 0.981756706 |
| ko03440 | Homologous recombination | 1 | 97 | 0.9835901 |
| ko04960 | Aldosterone-regulated sodium reabsorption | 1 | 98 | 0.98426491 |
| ko04071 | Sphingolipid signaling pathway | 4 | 223 | 0.984744759 |
| ko04720 | Long-term potentiation | 5 | 264 | 0.986876625 |
| ko04723 | Retrograde endocannabinoid signaling | 1 | 107 | 0.989217548 |
| ko05010 | Alzheimer's disease | 9 | 407 | 0.989422352 |
| ko04930 | Type II diabetes mellitus | 1 | 110 | 0.990494132 |
| ko05168 | Herpes simplex infection | 3 | 200 | 0.990549036 |
| ko04141 | Protein processing in endoplasmic reticulum | 10 | 447 | 0.991196551 |
| ko03022 | Basal transcription factors | 1 | 116 | 0.992611992 |
| ko04932 | Non-alcoholic fatty liver disease (NAFLD) | 2 | 171 | 0.994083577 |
| ko04022 | cGMP-PKG signaling pathway | 5 | 293 | 0.994381582 |
| ko05210 | Colorectal cancer | 2 | 175 | 0.994897659 |
| ko05016 | Huntington's disease | 4 | 259 | 0.995007639 |
| ko04914 | Progesterone-mediated oocyte maturation | 2 | 178 | 0.995435079 |
| ko04350 | TGF-beta signaling pathway | 1 | 128 | 0.99553782 |
| ko04010 | MAPK signaling pathway | 4 | 264 | 0.995742097 |
| ko05160 | Hepatitis C | 1 | 131 | 0.99606639 |
| ko03013 | RNA transport | 5 | 305 | 0.996082882 |
| ko03410 | Base excision repair | 1 | 133 | 0.996383528 |
| ko05200 | Pathways in cancer | 4 | 271 | 0.996597459 |
| ko00563 | Glycosylphosphatidylinositol(GPI)-anchor biosynthesis | 1 | 138 | 0.997069031 |
| ko00640 | Propanoate metabolism | 1 | 140 | 0.997305375 |
| ko04120 | Ubiquitin mediated proteolysis | 4 | 289 | 0.99810263 |
| ko04724 | Glutamatergic synapse | 3 | 248 | 0.998163031 |
| ko05012 | Parkinson's disease | 2 | 210 | 0.998625325 |
| ko00510 | N-Glycan biosynthesis | 1 | 159 | 0.998787922 |
| ko04111 | Cell cycle - yeast | 1 | 168 | 0.999169925 |
| ko03420 | Nucleotide excision repair | 1 | 171 | 0.999268348 |
| ko04650 | Natural killer cell mediated cytotoxicity | 2 | 228 | 0.999306418 |
| ko04380 | Osteoclast differentiation | 2 | 228 | 0.999306418 |
| ko04660 | T cell receptor signaling pathway | 2 | 230 | 0.999357408 |
| ko04662 | B cell receptor signaling pathway | 2 | 235 | 0.999469231 |
| ko04370 | VEGF signaling pathway | 2 | 237 | 0.999508362 |
| ko00970 | Aminoacyl-tRNA biosynthesis | 1 | 188 | 0.99964222 |
| ko03040 | Spliceosome | 7 | 496 | 0.99987988 |
| ko03015 | mRNA surveillance pathway | 1 | 215 | 0.999885214 |
| ko05166 | HTLV-I infection | 2 | 281 | 0.999910122 |
| ko04360 | Axon guidance | 1 | 230 | 0.999938984 |
| ko04113 | Meiosis - yeast | 1 | 301 | 0.999996945 |
