# Supplementary material for: De novo Sequencing and Transcriptome Analysis Reveal Key Genes Regulating Steroid Metabolism in Leaves, Roots, Adventitious Roots and Calli of Periploca sepium Bunge
Source: Front Plant Sci. 2017 Apr 21;8:594. doi: 10.3389/fpls.2017.00594 (PMC5399629; doi:10.3389/fpls.2017.00594)
Supplement: Supplementary file 13 [file Table13.DOC]

**Table S13. RPKM values of the genes involved in the IPP biosynthesis from the MVA and MEP pathway.**

| **Enzymes name** | **Abbreviation** | **RPKM value** | | | |
| --- | --- | --- | --- | --- | --- |
|  |  | **AR** | **C** | **R** | **L** |
| acetyl-CoA acetyltransferase | ACAT | 452.8* | 206* | 111.1 | 74 |
| HMG-CoA synthase | HMGS | 211.9* | 63.6 | 45 | 45.1 |
| HMG-CoA reductase | HMGR | 318.9 | 159.7 | 53.1** | 273.4 |
| mevalonate kinase | MK | 61.7 | 73.4 | 23.4** | 61.5 |
| phosphomevalonate kinase | PMK | 17.3 | 16.3 | 13.3 | 9.3 |
| mevalonate diphosphate decarboxylase | MDD | 141.5* | 146.3* | 33.4 | 37.1 |
| isopentenylpyrophosphate isomerase | IPPI | 64.4* | 16.4 | 7.8 | 13.7 |
| farnesyl pyrophosphate synthase | FPS | 80.9 | 166.7* | 61.9 | 46.3 |
| DXP synthase | DXS | 47 | 20.7** | 106 | 86.2 |
| DXP reductoisomerase | DXR | 34.7 | 29.9 | 40.2 | 54.7 |
| MEP cytidylytransferase | MCT | 3.9** | 2.5** | 4 | 12.2 |
| CDP-ME kinase | CMK | 4.7** | 6.9 | 7.8 | 13.7 |
| MEC synthase | MCS | 6.2** | 6.1** | 10.8 | 22.5 |
| HMBPP synthase | HDS | 0** | 0** | 6.14 | 16.4 |

*Note*: The genes encoding AATC, HMGS, HMGR, MK, PMR,MDD, IPPI and FPS are involved in MVA pathway. And, the genes encoding DXS, DXR, MCT, CMK, MCS and HDS are involved in MEP pathway. The genes of RPKM value marked ‘*’ meant up-regulated genes, and the genes of RPKM value marked ‘**’ meant down-regulated genes.
