# Supplementary material for: De novo Sequencing and Transcriptome Analysis Reveal Key Genes Regulating Steroid Metabolism in Leaves, Roots, Adventitious Roots and Calli of Periploca sepium Bunge
Source: Front Plant Sci. 2017 Apr 21;8:594. doi: 10.3389/fpls.2017.00594 (PMC5399629; doi:10.3389/fpls.2017.00594)
Supplement: Supplementary file 14 [file Table14.DOC]

**Table S14. RPKM values of the genes involved in the midstream and downstream biosynthesis of the steroids pathway.**

| **Enzymes name** | **Abbreviation** | AR | C | R | L |
| --- | --- | --- | --- | --- | --- |
| squalene synthase | SS | 334.5* | 133.1 | 130.2 | 112.9 |
| squalene epoxidase | SE | 21.4* | 32.8* | 6.3 | 5.1 |
| cycloartenol synthase | CAS | 64.4 | 52** | 58.1 | 106.2 |
| sterol C-24 methyltransferase | SMT1 | 441.8* | 213.9* | 339.8* | 83.8 |
| sterol-4alpha-methyl oxidase 1 | SMO1 | 226* | 147.2 | 137.6 | 92.4 |
| sterol 14alpha-demethylase | CYP51/  14-SDM | 186.7* | 154.6* | 66.5 | 68.2 |
| delta(14)-sterol reductase | FK/14SR | 67.3 | 50.3 | 50.2 | 36.8 |
| C-8,7 sterol isomerase | HYD1/EBP | 35.6* | 37.7* | 15.3 | 14.4 |
| sterol-4alpha-methyl oxidase 2 | SMO2 | 98.2* | 88.3* | 18.7 | 25.2 |
| delta(7)-sterol-C5(6)-desaturase | STE1/SC5DL | 39.7 | 34.1 | 27.5 | 31.4 |
| 7-dehydrocholesterol reductase | DWF5/  DHCR7 | 205.8* | 214.6* | 110.9 | 68.6 |
| [delta (24)-sterol reductase](http://www.so.com/link?url=http%3A%2F%2Fwww.ncbi.nlm.nih.gov%2Fgene%2F821519&q=DWF1+campesterol&ts=1474893219&t=8d0e9ba903ece9615739e5e3a4e8fe2&src=haosou) | DWF1/  DHCR24 | 138.5** | 175.8 | 336.8 | 280.4 |
| sterol C-22 desaturase | CYP710A | 14.9* | 0 | 1.1 | 1.8 |
| 3beta-hydroxysteroid-dehydrogenase | 3β-HSD | 31.9* | 49.4* | 31.1* | 13.8 |
| progesterone 5beta-reductase | 5β-POR | 7.3 | 7.6 | 18.8 | 15.4 |

*Note*: The genes of RPKM value marked ‘*’ meant up-regulated genes, and the genes of RPKM value marked ‘**’ meant down-regulated genes.
