# Supplementary material for: De novo Sequencing and Transcriptome Analysis Reveal Key Genes Regulating Steroid Metabolism in Leaves, Roots, Adventitious Roots and Calli of Periploca sepium Bunge
Source: Front Plant Sci. 2017 Apr 21;8:594. doi: 10.3389/fpls.2017.00594 (PMC5399629; doi:10.3389/fpls.2017.00594)
Supplement: Supplementary file 15 [file Presentation1.PDF]

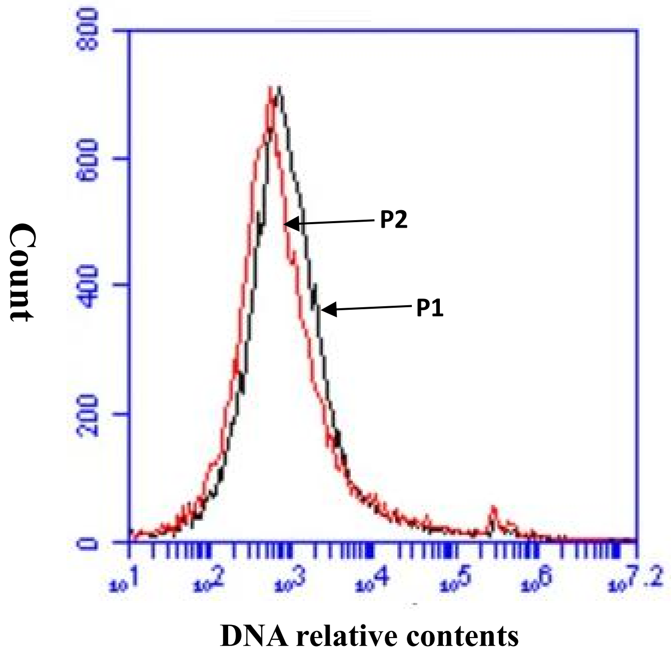

Figure S1. Results for *Periploca sepium* and *Arabidopsis* young leaves mixed samples from the flow cytometry analysis. P1: Peak of 2 C of *P. sepium*, peak value is 790; P2: Peak of 2 C of Arabidopsis, peak value is 567
