# Supplementary material for: De novo Sequencing and Transcriptome Analysis Reveal Key Genes Regulating Steroid Metabolism in Leaves, Roots, Adventitious Roots and Calli of Periploca sepium Bunge
Source: Front Plant Sci. 2017 Apr 21;8:594. doi: 10.3389/fpls.2017.00594 (PMC5399629; doi:10.3389/fpls.2017.00594)
Supplement: Supplementary file 16 [file Presentation2.PDF]

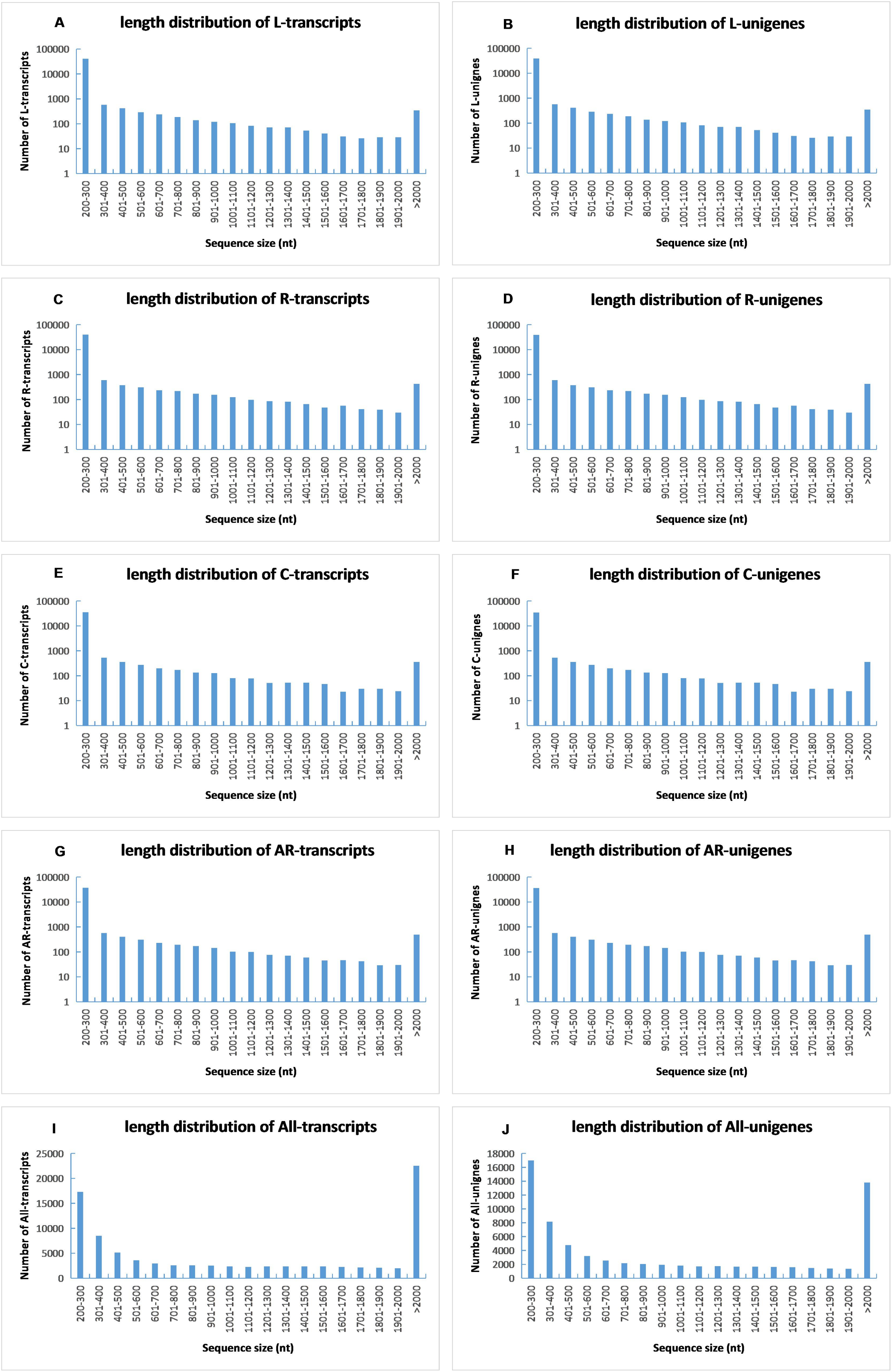

Figure S2. Length distributions of the transcripts and unigenes. The length distribution of L-transcripts (A), L-unigenes (B), R-transcripts (C), R-unigenes (D), C-transcripts (E), C-unigenes (F), AR-transcripts (G), AR-unigenes (H), All-transcripts (I), and All-unigenes (J).
