# Supplementary material for: De novo Sequencing and Transcriptome Analysis Reveal Key Genes Regulating Steroid Metabolism in Leaves, Roots, Adventitious Roots and Calli of Periploca sepium Bunge
Source: Front Plant Sci. 2017 Apr 21;8:594. doi: 10.3389/fpls.2017.00594 (PMC5399629; doi:10.3389/fpls.2017.00594)
Supplement: Supplementary file 17 [file Presentation3.PDF]

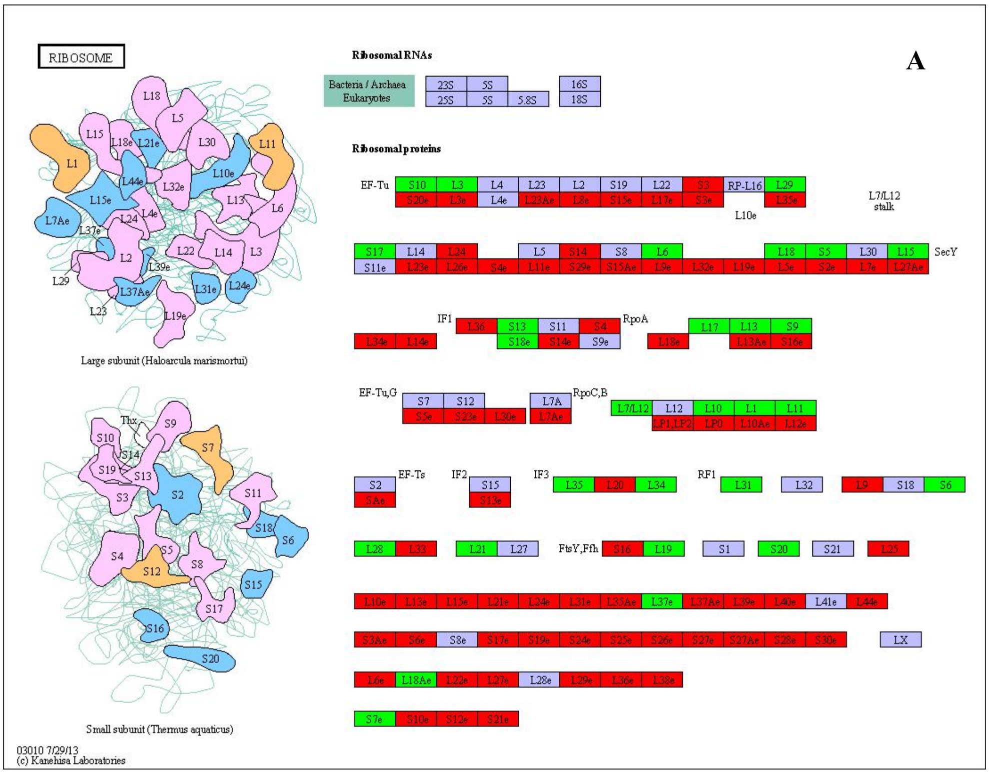
A
RIBOSOME
Ribosomal RNAs
Bacteria / Archaea
Eukaryotes
23S
5S
16S
25S
5S
5.8S
18S
Ribosomal proteins
EF-Tu
RP-L16
L10e
L7/L12 stalk
SecY
IF1
RpoA
EF-Tu,G
RpoC,B
EF-Ts
IF2
IF3
RF1
FtsY,Ffh
Large subunit (Haloarcula marismortui)
Small subunit (Thermus aquaticus)
03010 7/29/13
(c) Kanehisa Laboratories

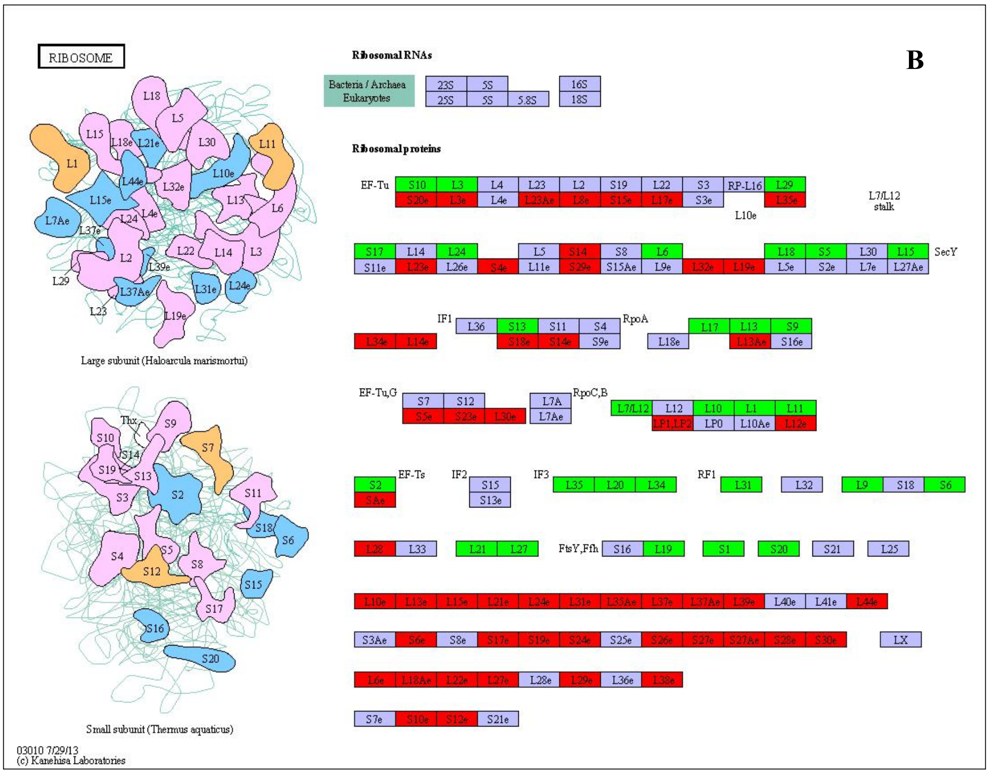
B
RIBOSOME
Ribosomal RNAs
Bacteria / Archaea
Eukaryotes
23S
5S
16S
25S
5S
5.8S
18S
Ribosomal proteins
EF-Tu
RP-L16
L10e
L7/L12 stalk
SecY
IF1
RpoA
EF-Tu,G
RpoC,B
EF-Ts
IF2
IF3
RF1
FtsY,Ffh
Large subunit (Haloarcula marismortui)
Small subunit (Thermus aquaticus)
03010 7/29/13
(c) Kanehisa Laboratories

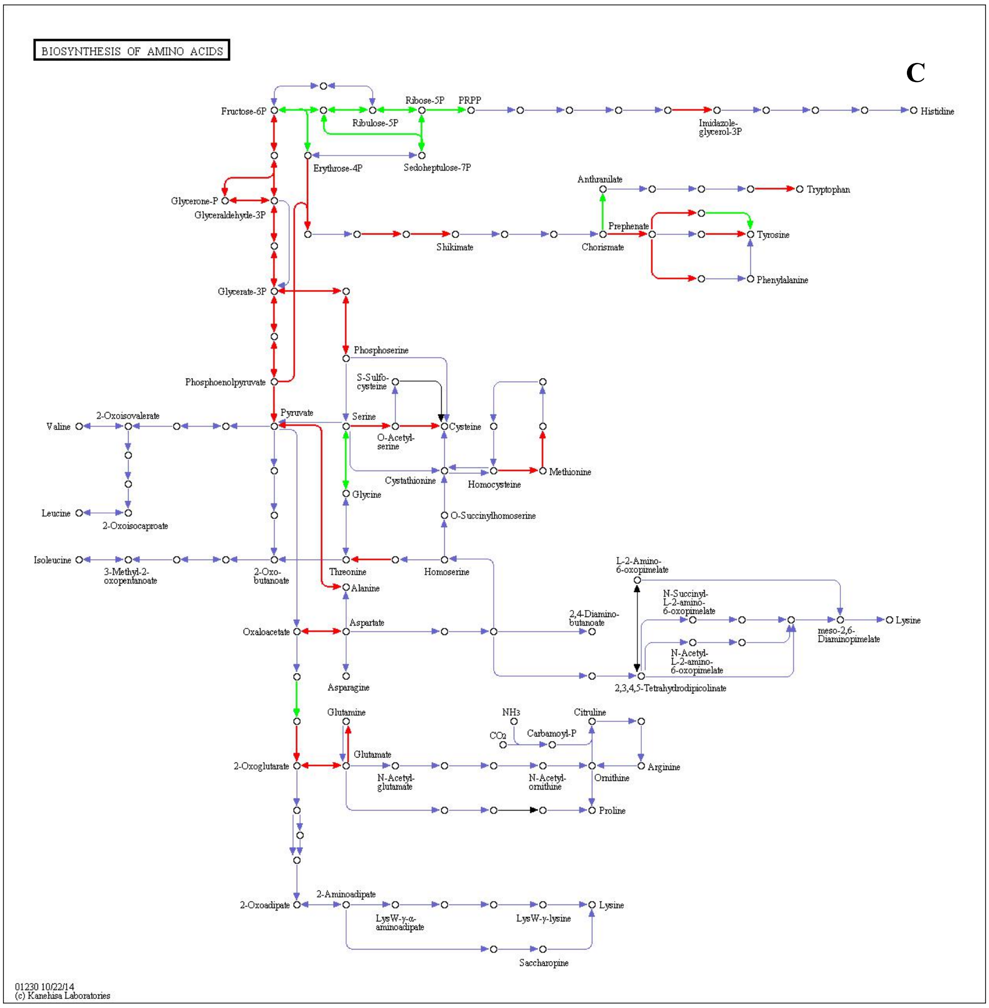
BIOSYNTHESIS OF AMINO ACIDS
C
Fructose-6P
Ribose-5P
PRPP
Ribulose-5P
Imidazole-glycerol-3P
Histidine
Erythrose-4P
Sedoheptulose-7P
Anthranilate
Tryptophan
Glycerone-P
Glyceraldehyde-3P
Prephenate
Shikimate
Chorismate
Tyrosine
Phenylalanine
Glycerate-3P
Phosphoserine
Phosphoenolpyruvate
S-Sulfo-cysteine
2-Oxoisovalerate
Pyruvate
Serine
Valine
Cysteine
O-Acetyl-serine
Methionine
Cystathionine
Homocysteine
Glycine
Leucine
2-Oxoisocaproate
O-Succinylhomoserine
Isoleucine
3-Methyl-2-oxopentanoate
2-Oxo-butanoate
Threonine
Homoserine
L-2-Amino-6-oxopimelate
Alanine
N-Succinyl-L-2-amino-6-oxopimelate
2,4-Diamino-butanoate
Aspartate
Lysine
Oxaloacetate
meso-2,6-Diaminopimelate
N-Acetyl-L-2-amino-6-oxopimelate
Asparagine
2,3,4,5-Tetrahydrodipicolinate
Glutamine
NH3
Citruline
CO2
Carbamoyl-P
Glutamate
2-Oxoglutarate
Arginine
N-Acetyl-glutamate
N-Acetyl-ornithine
Ornithine
Proline
2-Aminoadipate
2-Oxoadipate
Lysine
LysW-γ-α-aminoadipate
LysW-γ-lysine
Saccharopine
01230 10/22/14
(c) Kanehisa Laboratories

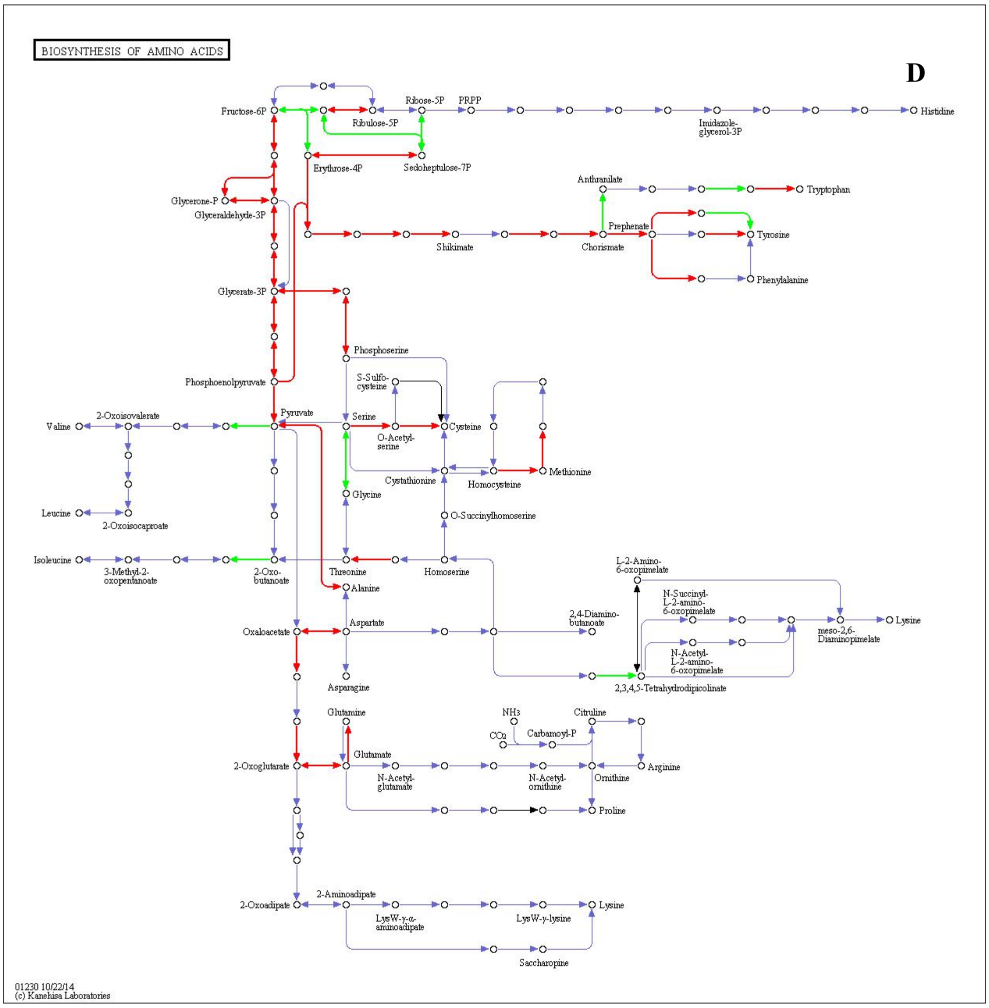

Figure S3. The KEGG pathway of ribosome and biosynthesis of amino acids. The terms of the ribosome pathway in C vs. L (A), AR vs. L (B) and the terms of the biosynthesis of amino acids pathway in C vs. L (C), AR vs. L (D).
