# Supplementary material for: De novo Sequencing and Transcriptome Analysis Reveal Key Genes Regulating Steroid Metabolism in Leaves, Roots, Adventitious Roots and Calli of Periploca sepium Bunge
Source: Front Plant Sci. 2017 Apr 21;8:594. doi: 10.3389/fpls.2017.00594 (PMC5399629; doi:10.3389/fpls.2017.00594)
Supplement: Supplementary file 18 [file Presentation4.PDF]

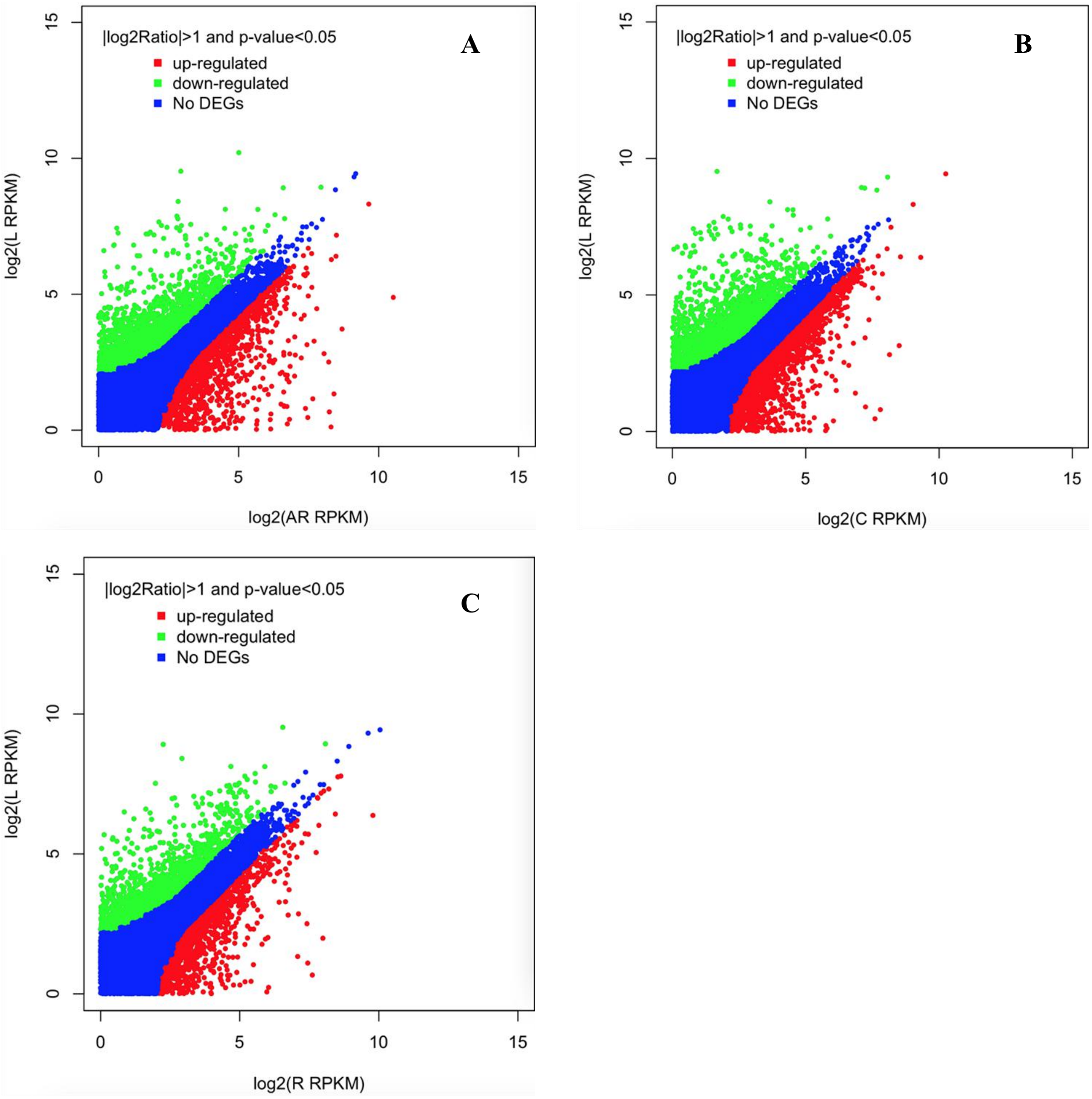

Figure S4. Distribution of the unigene changes in the three samples compared with leaves. Red represents up-regulation, green represents down-regulation, and the blue represents no significant difference detected. (A) The expression levels of the unigenes in AR compared with L; (B) The expression levels of the unigenes in C compared with L; and (C) The expression levels of the unigenes in R compared with L.
