# Supplementary material for: De novo Sequencing and Transcriptome Analysis Reveal Key Genes Regulating Steroid Metabolism in Leaves, Roots, Adventitious Roots and Calli of Periploca sepium Bunge
Source: Front Plant Sci. 2017 Apr 21;8:594. doi: 10.3389/fpls.2017.00594 (PMC5399629; doi:10.3389/fpls.2017.00594)
Supplement: Supplementary file 19 [file Presentation5.PDF]

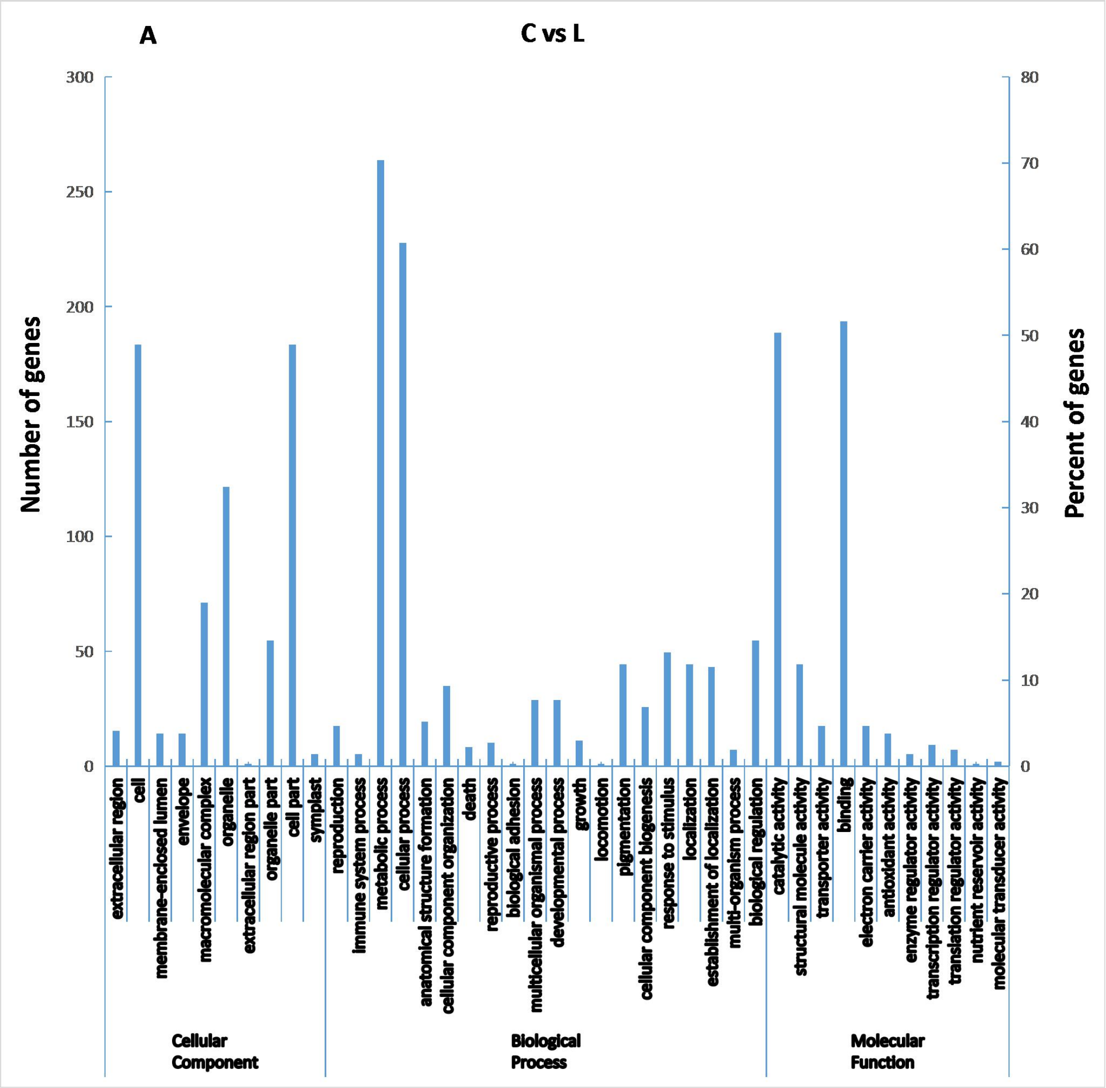

A
C vs L
Number of genes
Percent of genes
300
250
200
150
100
50
0
80
70
60
50
40
30
20
10
0
extracellular region
cell
membrane-enclosed lumen
envelope
macromolecular complex
organelle
extracellular region part
organelle part
cell part
symplast
reproduction
immune system process
metabolic process
cellular process
anatomical structure formation
cellular component organization
death
reproductive process
biological adhesion
multicellular organismal process
developmental process
growth
locomotion
pigmentation
cellular component biogenesis
response to stimulus
localization
establishment of localization
multi-organism process
biological regulation
catalytic activity
structural molecule activity
transporter activity
binding
electron carrier activity
antioxidant activity
enzyme regulator activity
transcription regulator activity
translation regulator activity
nutrient reservoir activity
molecular transducer activity
Cellular Component
Biological Process
Molecular Function

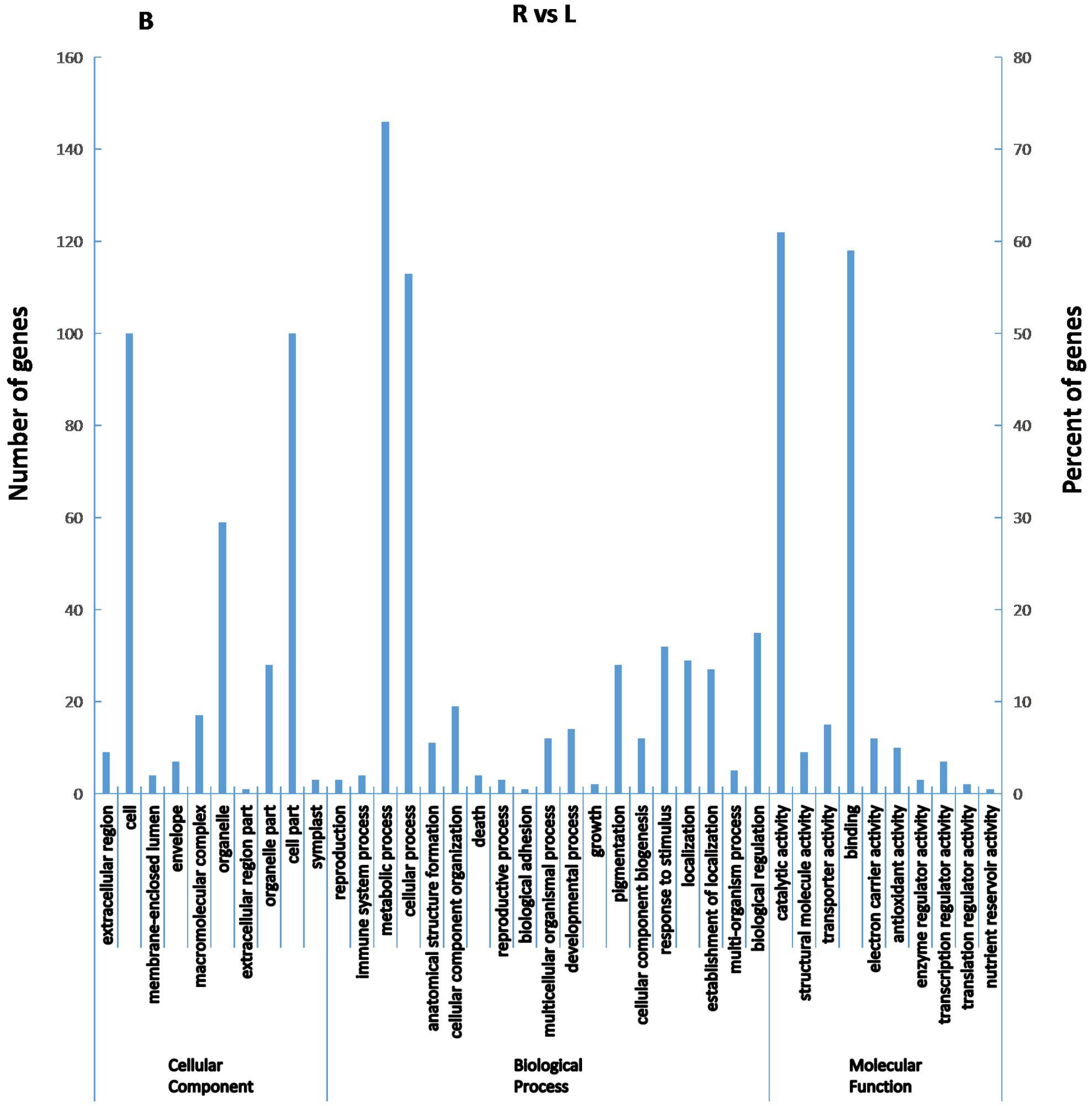
B
R vs L
Number of genes
Percent of genes
160
140
120
100
80
60
40
20
0
80
70
60
50
40
30
20
10
0
extracellular region
cell
membrane-enclosed lumen
envelope
macromolecular complex
organelle
extracellular region part
organelle part
cell part
symplast
reproduction
immune system process
metabolic process
cellular process
anatomical structure formation
cellular component organization
death
reproductive process
biological adhesion
multicellular organismal process
developmental process
growth
pigmentation
cellular component biogenesis
response to stimulus
localization
establishment of localization
multi-organism process
biological regulation
catalytic activity
structural molecule activity
transporter activity
binding
electron carrier activity
antioxidant activity
enzyme regulator activity
transcription regulator activity
translation regulator activity
nutrient reservoir activity
Cellular Component
Biological Process
Molecular Function

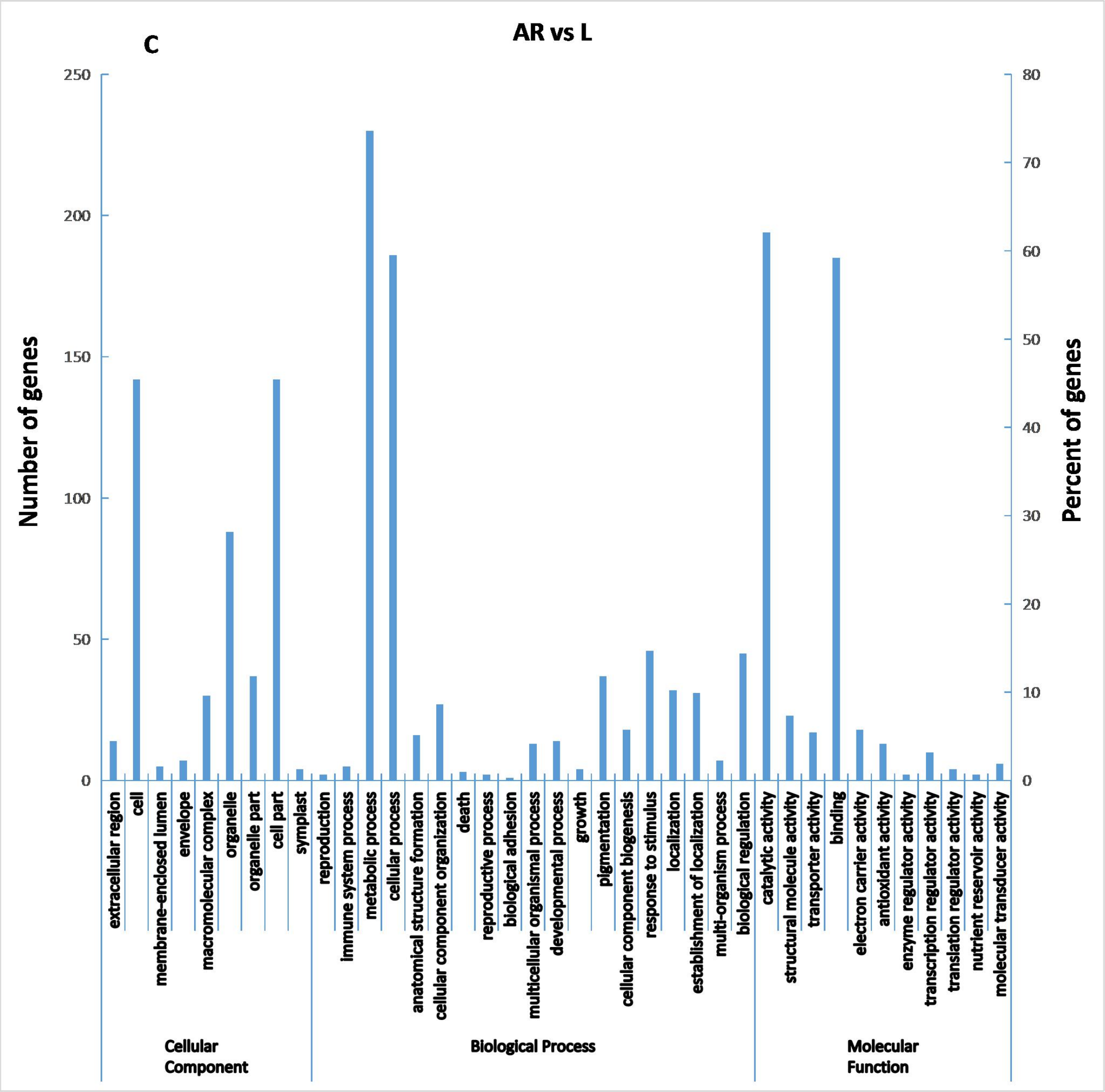

Figure S5. Histograms of the GO classification of DEGs. The DEGs in C compared with L (A), R compared with L (B), AR compared with L (C) are summarized in three main categories: cellular component, molecular function, and biological process. The y-axis on the left side is the number of genes; the y-axis on the right side is the percentage of genes in a given category.
